# Room-Temperature Nucleophilic Aromatic Substitution of 2‑Halopyridinium Ketene Hemiaminals with Sulfur Nucleophiles

**DOI:** 10.1021/acsomega.5c07504

**Published:** 2025-11-17

**Authors:** Jordan C. Merklin, Beau A. Sinardo, Claire Y. Cooper, Sudchananya Udomphan, Madeleine H. Boger, Max M. Majireck

**Affiliations:** Chemistry Department, 2576 Hamilton College, 198 College Hill Rd, Clinton, New York 13323, United States

## Abstract

2-Thiopyridines and their derivatives are a valuable class of bioactive compounds for drug discovery. However, many synthetic approaches toward these compounds rely on metal catalysis, high-boiling solvents, and/or elevated reaction temperatures. Herein we report a simple mix-and-stir protocol for the synthesis of novel 2-thiopyridiniums, leveraging the recently developed reagent 2-chloro-1-(1-ethoxyvinyl)­pyridinium triflate and readily accessible thiol or thiolate nucleophiles.

## Graphical abstract

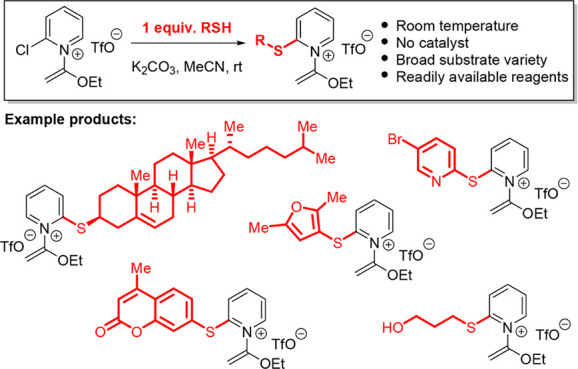

## 1 Introduction

2-Thiopyridines are a versatile compound class for drug discovery, as they are found in a broad range of bioactive substances, including antibacterial,^1^ antiviral,^2^ and antitumor agents.^3^ Consequently, numerous synthetic strategies toward this construct have been developed. In recent years, a broad range of metal-catalyzed cross-couplings have been designed using a large variety of coupling partners, catalysts, and ligands (Scheme 1A).^4^ Despite the wealth of options within this approach, metal contamination remains a significant issue when screening for bioactivity,^5^ particularly with higher catalyst loadings and/or highly polar products. Frequently, conventional substitution-based approaches are adopted due to their simplicity. One classic approach leverages 2-mercaptopyridine nucleophiles in bimolecular nucleophilic substitutions with a suitable electrophile (Scheme 1B).^6^ Though typically straightforward, this protocol is generally incompatible with sterically hindered electrophiles, and the competitive *N*-alkylation pathway is often favored over *S*-alkylation.^3,7^ Nucleophilic aromatic substitution (S_N_Ar) of electrophilic pyridines (e.g., 2-halopyridines) provides a complementary strategy in which a broad range of available thiol and thiolate nucleophiles can be employed (Scheme 1C).^8^ However, these methods often require elevated temperatures, strong bases, highly polar solvents, and/or strategically positioned electron-withdrawing groups on the pyridine ring, imposing a number of practical limitations on this otherwise general approach to 2-thiopyridines. Nevertheless, continual improvements in S_N_Ar reaction design for aryl and heteroaryl thioethers remain an active area of research^9^ due to the centrality of this process in the synthesis of bioactive thioether-containing drugs.^10^

**Scheme 1:**
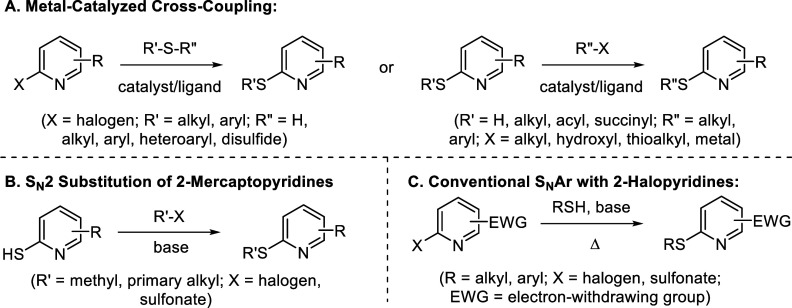
Common Approaches to the Synthesis of 2-Thiopyridines

Recently, we discovered that 2-halopyridinium ketene hemiaminals are exceptionally reactive toward nucleophilic aromatic substitutions with alkyl and aryl amines, various carbon nucleophiles (e.g., malonates and indoles), and, in one preliminary example, a thiol.^11^ Owing to the exceptional nucleophilicity of thiols and thiolates in nucleophilic aromatic substitutions and the enhanced electrophilicity of 2-halopyridinium ketene hemiaminals in the same reaction modality, we hypothesized that these features might act synergistically to promote S_N_Ar reactions at room temperature.

## 2 Results and Discussion

Given the range of potential thiol nucleophiles available for nucleophilic aromatic substitutions with **1**, we chose to run parallel optimization experiments with three distinct substrates: primary alkylthiol **2** and heteroarylthiols **3** and **4** (Table 1). All reactions were run on a 0.3 mmol scale and purified by column chromatography. 1-Octanethiol (**2**) reacted well with 2-chloropyridinium **1a** in a range of nonpolar and polar solvents (entries 1–7), and parallel reactions with 5-bromopyridine-2-thiol (**3**) displayed similar trends (entries 8–11). Contrastingly, the benzoxazole-containing nucleophile **4** was overall less compatible, yielding complex mixtures in most cases (entries 16, 17, and 19) except for limited formation of **7** when using acetonitrile (entry 18). Using thiol **3** as the nucleophile, we also examined the substitution of the significantly less electrophilic bromo- and iodo-analogues **1b**–**c**, both of which led to product **6** in only somewhat lower yield (entries 14 and 15). Since the 24-h stirring period was set as a matter of convenience, we also performed shorter reactions using electrophile **1a** and nucleophile **3**. Interestingly, the reaction still proceeded at 0 °C (entry 13), and a higher yield was obtained at room temperature when halting the reaction at 1 h (entry 12), an effect that may be related to competitive hydrolysis of reagent **1a** and/or product **6** over the course of the reaction.

**Table 1:**
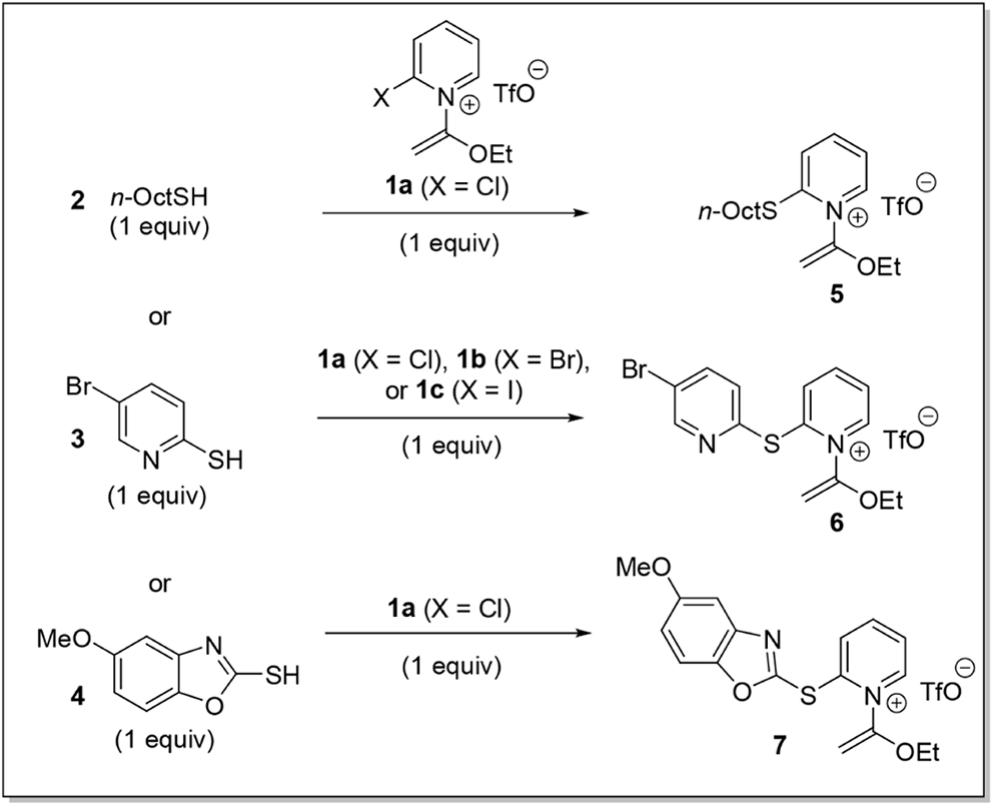
Parallel Reaction Optimization

Entry	X	RSH	Solvent	Time	Temp	Isolated Yield^a^
1	Cl	2	CH_2_Cl_2_	24 h	18 °C	5, 63%
2	Cl	2	PhH	24 h	18 °C	5, 39%
3	Cl	2	PhMe	24 h	18 °C	5, 89%
4	Cl	2	THF	24 h	18 °C	5, 86%
5	Cl	2	MeCN	24 h	18 °C	5, 92%
6	Cl	2	iPrOH	24 h	18 °C	5, 94%
7	Cl	2	H_2_O	24 h	18 °C	5, 29%
8	Cl	3	PhMe	24 h	18 °C	6, 84%
9	Cl	3	THF	24 h	18 °C	6, 83%
10	Cl	3	MeCN	24 h	18 °C	6, 85%
11	Cl	3	iPrOH	24 h	18 °C	6, 70%
12	Cl	3	MeCN	1 h	18 °C	6, 94%
13	Cl	3	MeCN	1 h	0 °C	6, 69%
14	Br	3	MeCN	24 h	18 °C	6, 70%
15	I	3	MeCN	24 h	18 °C	6, 81%
16	Cl	4	PhMe	24 h	18 °C	complex mix
17	Cl	4	THF	24 h	18 °C	complex mix
18	Cl	4	MeCN	24 h	18 °C	7, 10%
19	Cl	4	iPrOH	24 h	18 °C	complex mix

a Purified by silica gel column chromatography.

To evaluate the scope of participating nucleophiles, we began by screening a representative group of primary, secondary, and tertiary alkyl thiols using acetonitrile, the optimal solvent, and a 24-h reaction time for the practical convenience of our undergraduate research laboratory (Scheme 2). Overall, we were pleased that a majority of substrates delivered their corresponding S_N_Ar products (**5**, **8**–**12**) in high yield. Similarly, benzyl and aryl thiols bearing a variety of functional groups with diverse electronic and steric factors generated compounds **13**, **18**–**23**, and **25–27**, with only the hindered and electron-poor nitroaryl analogue **24** failing to be produced. In a few cases, we tested whether the corresponding sodium thiolate could be used as the nucleophile. Conveniently, using either iPrSNa or PhSNa, with omission of the potassium carbonate base, led to even higher yields when compared to our standard procedure (cf. **10** and **18**).

**Scheme 2:**
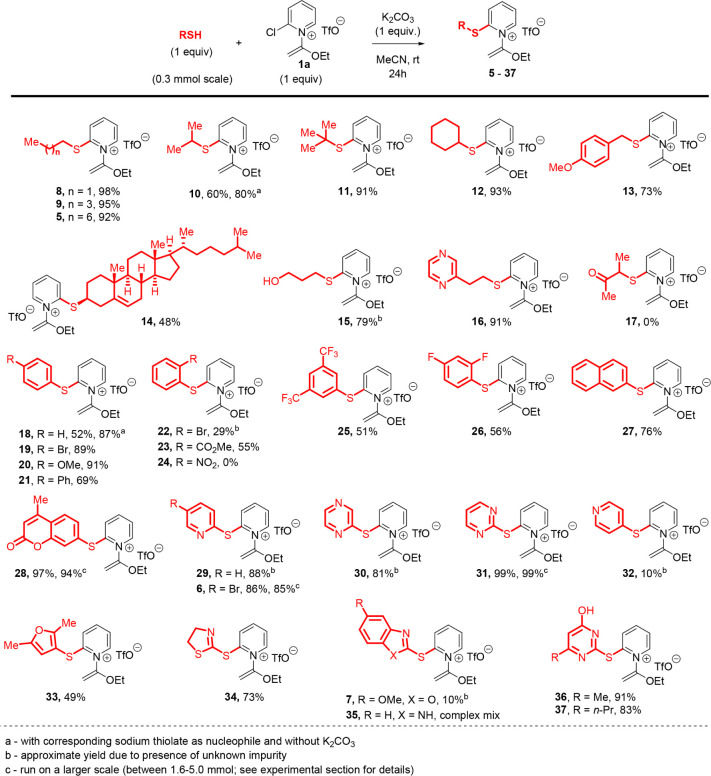
Scope of Thiol Nucleophiles

To further evaluate the functional group tolerance of this method, we successfully generated more complex 2-thiopyridinium salts derived from alkyl and aryl thiols, including those bearing complex ring systems, hydroxyl groups, and basic nitrogen atoms (**14**, **15**, and **16**, respectively; Scheme 2). Conversely, the ketone-containing thiol 3-mercapto-2-butanone was found to be incompatible with this procedure, failing to deliver **17**. A range of heteroaryl thiols commonly used in medicinal chemistry were examined, leading to 2-thiopyridinium salts appended to a coumarin (**28**), furan (**33**), pyridines (**6** and **29**), diazines (**30** and **31**), thiazoline (**34**), and thiouracils (**36** and **37**) in moderate to good yield. Three products (**6**, **28**, and **31**) were synthesized by gram-scale procedures, delivering these compounds in essentially the same yields when compared with the standard 0.3 mmol (milligram) scale. Contrastingly, the 4-mercaptopyridine analogue **32**, benzoxazole **7**, and benzimidazole **35** delivered their products in lower yield among more complex mixtures.

Several examples involving other 2-chloropyridinium electrophiles were also examined (Scheme 3). The 2,3-dichloropyridinium salt analogue **38** provided product **39** in a low yield when using 5-bromo-2-mercaptopyridine as the nucleophile. Presumably, the extra chlorine atom of **38** will enhance the electrophilicity toward substitution but also competitive hydrolysis under these conditions. Bulkier 2-chloropyridinium salts **40**, available as an *E*/*Z* mixture, were amenable to these conditions, delivering products **41** and **42** as *E*/*Z* mixtures, the latter of which was separable by silica gel column chromatography.

**Scheme 3:**
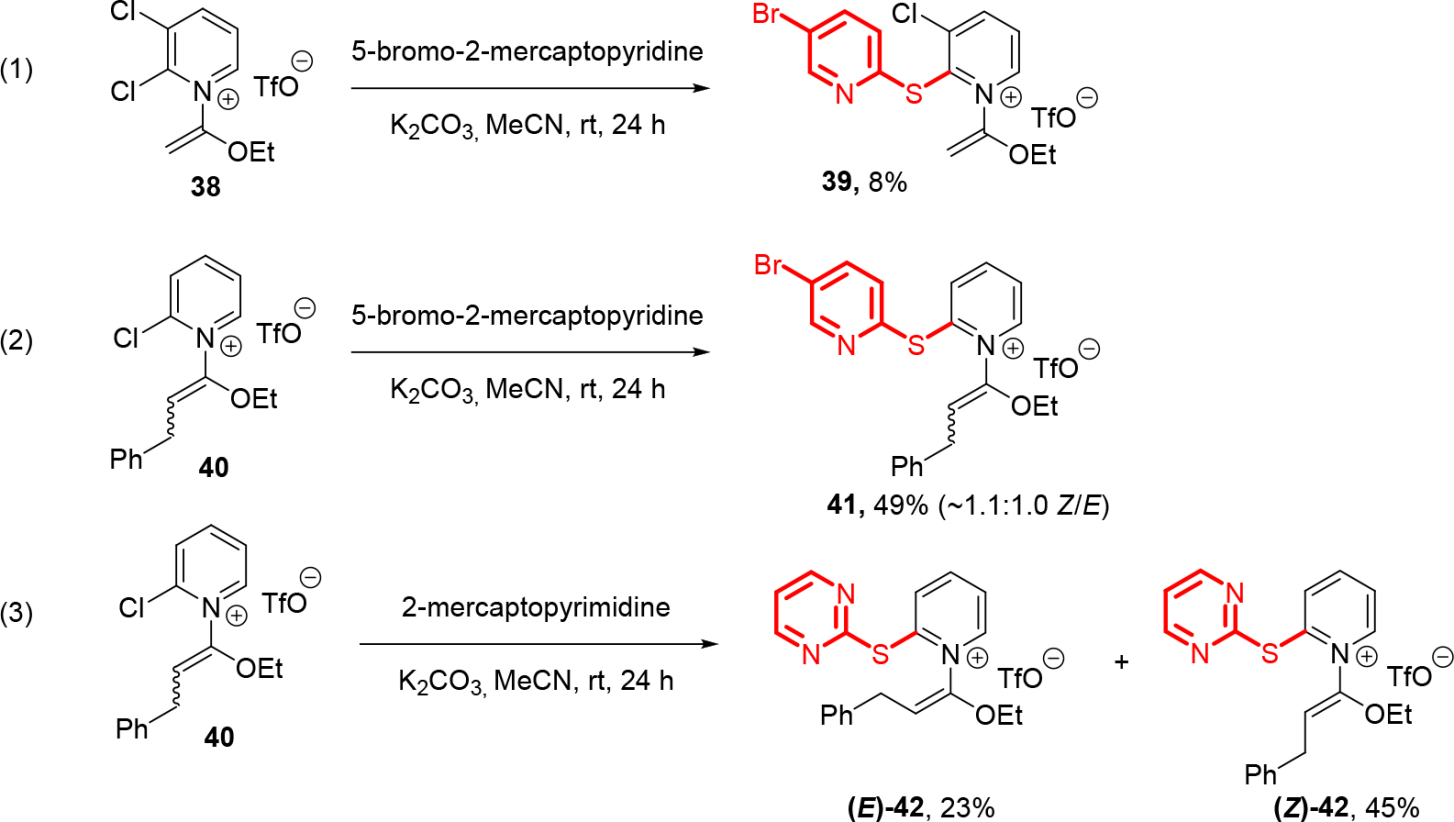
Brief Evaluation of Alternative Electrophiles

Overall, column chromatography was the most general method for the purification of the 2-thiopyridinium S_N_Ar products noted above. Once isolated, the majority of these products were found to be stable for over 12 months when stored neat at room temperature. However, it is important to note that in some cases, minor decomposition was observed during chromatographic purification on silica gel. The highly polar and ionic nature of 2-thiopyridinium ketene hemiaminals made it difficult to completely eliminate minor impurities by this method. At present, we have yet to develop a more general purification method but will continue to actively explore alternative procedures such as recrystallization, trituration, ion-exchange resins, and reversed-phase chromatography.

Since the *N*-(1-ethoxyvinyl) group is maintained following nucleophilic aromatic substitution, we briefly examined procedures for cleaving this moiety on a representative collection of S_N_Ar products (Scheme 4). We found that simply dissolving several S_N_Ar products in a 4 M HCl 1,4-dioxane solution and warming to 50 °C overnight delivered their corresponding hydrochloride or triflate salts in good to moderate yields, usually without the need for further purification or anion exchange.

**Scheme 4:**
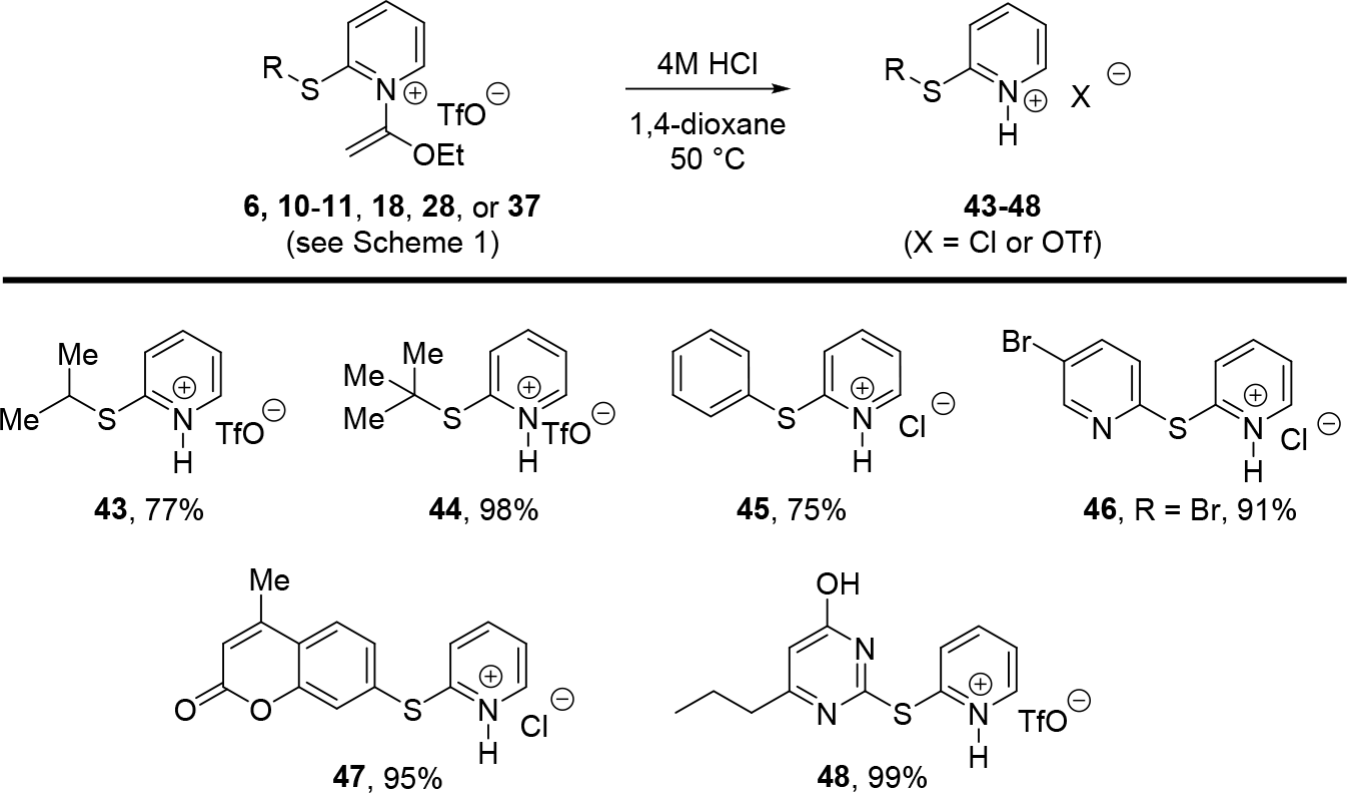
Acidic Removal of *N*-(1-Ethoxyvinyl) Group

Finally, given the prevalence of related 2-alkoxypyridines in medicinal chemistry,^12^ we hoped to establish a protocol for analogous *O-*nucleophiles. As expected, however, these weaker nucleophiles were more challenging to implement. To make up for the weak nucleophile strength, we first examined the use of alkoxides via direct addition (e.g., NaOEt) or initial deprotonation of an alcohol (e.g., with NaH); however, these reactions generally favored rapid hydrolysis of the 2-halopyridinium electrophile (e.g., **1a**) and/or the S_N_Ar product. We thus examined the use of the highly reactive 2-fluoropyridinium salt **1d**, which was formed in situ to avoid hydrolysis with atmospheric moisture (Scheme 5, entries 1–4). Most base additives favored the hydrolytic formation of α-pyridone **51** (e.g., entry 1). Triethylamine enabled the formation of the desired product **49**, but significant quantities of triethylammonium impurities contaminated the product (entry 2). Omission of the base altogether provided S_N_Ar product **49** at low yield when conducted at room temperature (entry 3), while warming the reaction mixture provided the *N*-H pyridinium in moderate yield (entry 4). We also briefly examined the use of the bench-stable reagent **1a** (entries 5–8). Adaptation of the conditions used for thiol nucleophiles, or a previously examined alcohol nucleophile,^11^ led primarily to hydrolysis (entries 5 and 6). Heating **1a** in a solution of n-butanol led to decomposition (entry 7); however, sonication of this solution, with concomitant heating, for a shorter period of time gave a combination of **49** and **50** as the major and minor products, respectively (entry 8).

**Scheme 5:**
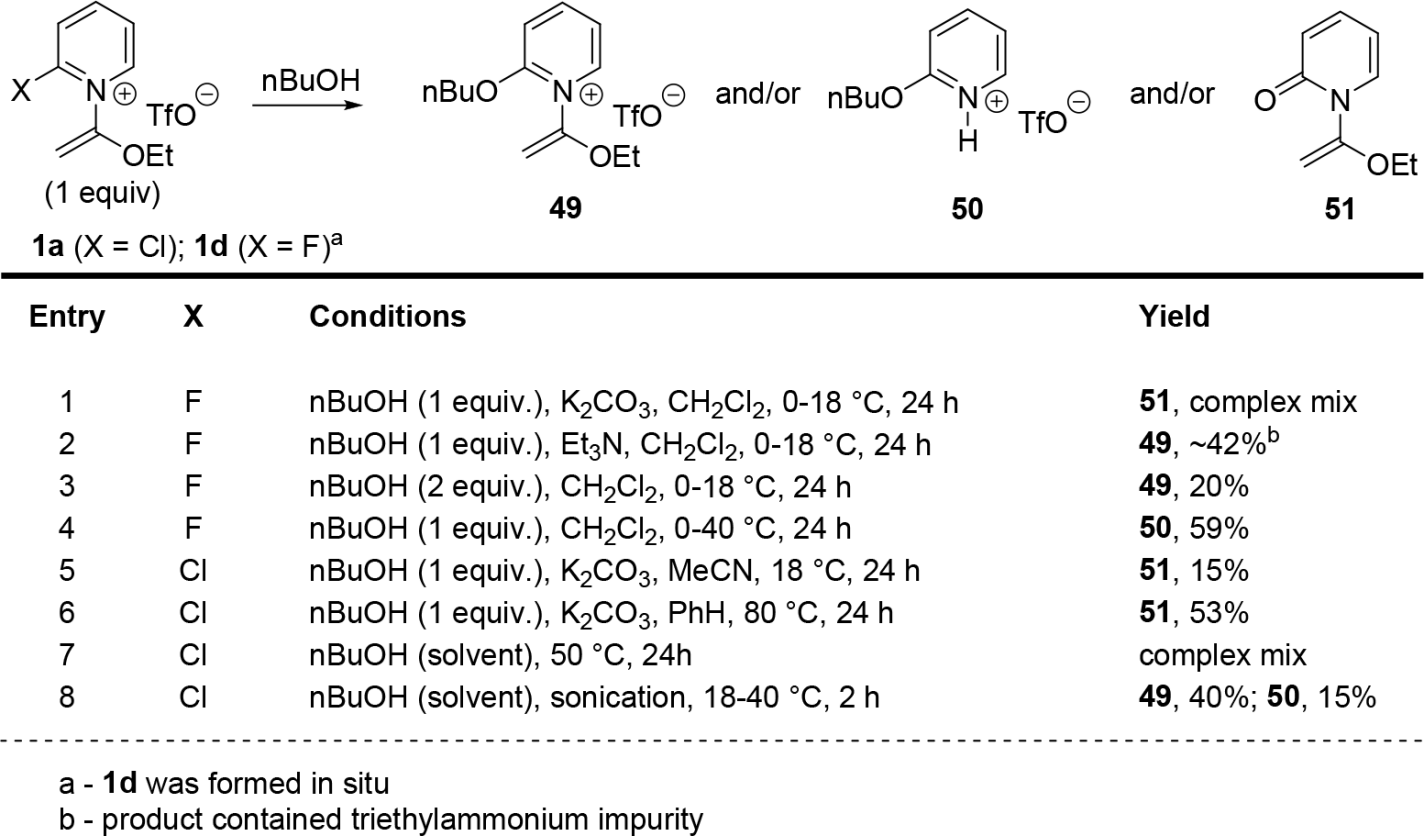
Brief Evaluation of Alcohol Nucleophiles

## 3 Conclusions

In summary, a broad range of thiols and thiolates participate in the nucleophilic aromatic substitution of 2-chloro-1-(1-ethoxyvinyl)­pyridinium triflate (**1a**), enabling rapid access to valuable 2-thiopyridines. Notably, this methodology is compatible with a variety of drug-like heterocyclic ring systems (e.g., pyridine, pyrimidine, thiazoline, and coumarin) as well as known bioactives (e.g., thiocholesterol derivative **14**, hymecromone thiol bioisostere derivative **28**, and compound **37**, an analogue of the antithyroid drug propylthiouracil). These straightforward and mild conditions complement existing procedures while circumventing common issues such as highly polar solvents, expensive catalysts, and/or elevated temperatures. We expect that the use of these methods will enable the synthesis and evaluation of novel bioactive 2-thiopyridines for future drug discovery efforts.

## 4 Experimental Section

### 4.1 General Information

Reagent **1a** was purchased from Oakwood Chemical or synthesized according to our *Organic Syntheses* protocol.^13^ Reagents **1b**–**d** were synthesized according to known procedures.^14^ All of the other chemicals purchased from commercial vendors were used without further purification. Conventional heating was conducted using a silicone oil bath on an IKA REA heating-stirring plate with an oil bath temperature probe. Sonication was performed in a Fisher Scientific ultrasonic water bath with an ∼50–60 Hz operating frequency.

Column chromatography was performed using new RediSep Rf Gold normal phase silica columns (20–40 μm) with a Teledyne Isco CombiFlash Rf200 purification system. We found automated chromatography to be the most convenient, but not essential. Standard column chromatography may be employed, but high-grade silica is recommended (e.g., fine spherical silica, 20–40 μM) in order to avoid contamination of broken-off silica in the product while using polar alcohol solvents (e.g., methanol or isopropanol). Multiple control experiments involving extensive washing of 12 g RediSep Rf Gold normal phase silica columns with pure methanol or isopropanol showed that little (<1 mg) to no silica was eluted. For compounds containing impurities or present as known mixtures, ^1^H NMR integrations were used to calculate more accurate yields of the desired product(s) or side product(s).

NMR spectra were obtained on a Bruker Avance 500 MHz spectrometer. For compounds containing impurities or present as mixtures, ^1^H NMR integrations were used to calculate accurate yields of the desired product(s) or side product(s). Both nominal and high-resolution mass spectra were obtained on a Waters Micromass 70-VSE.

### 4.2 General S_N_Ar Procedure for the Synthesis of 2-Thiopyridinium Salts

A 5 mL round-bottom flask was charged with 1-(1-ethoxyvinyl)-2-chloropyridinium triflate **1a** (100 mg, 0.300 mmol), 1 mL of anhydrous acetonitrile, thiol nucleophile (0.30 mmol), and then anhydrous potassium carbonate (42 mg, 0.30 mmol). The resulting mixture was stirred at room temperature for 24 h, then concentrated in vacuo to yield a residue that was purified by automated column chromatography with a Teledyne ISCO CombiFlash system (between 0 and 100% chloroform/isopropanol gradient; 4 g RediSep Gold prepacked silica gel cartridge) to yield the corresponding 2-thiopyridinium triflate S_N_Ar product.

### 4.3 General Procedure for Hydrolysis of *N*-(1-Ethoxyvinyl) Group

In a 0.5–2.0 mL Biotage microwave vial, *N*-(1-ethoxyvinyl)­pyridinium salt (50–100 μmol) was dissolved in a 4 M solution of HCl in 1,4-dioxane (1 mL), then capped and sealed. The resulting solution was placed in a preheated 50 °C oil bath and stirred for 24 h. The septum was carefully punctured with a needle to release pressure before removing the cap, then the solution was concentrated in vacuo to yield the product, generally without the need for further purification. Characterization Notes: The highly exchangeable N–H proton was often not observed by ^1^H NMR, presumably due to the high water, HDO, and D_2_O content in the acetone-d_6_. However, ^1^H and ^13^C chemical shifts are in accordance with N protonation of the pyridinium ring. The identity of the anion was assigned based on the presence or absence of the characteristic triflate *C*F_3_ quartet in the ^13^C NMR spectrum. The absence of this signal was presumed to be due to anion exchange of triflate with chloride; however, triflate signal intensities below the threshold of detection and/or anionic mixtures cannot be ruled out.

### 4.4 Synthetic Protocols and Characterization Data

#### 4.4.1 1-(1-Ethoxyvinyl)-2-(octylthio)­pyridin-1-ium Trifluoromethanesulfonate (**5**)

General S_N_Ar procedure was followed using 1-octanethiol (50.0 μL, 0.308 mmol) as the nucleophile. Purification by automated column chromatography with a Teledyne ISCO CombiFlash system (0–70% chloroform/isopropyl alcohol gradient) yielded product **5** as a pale yellow amorphous solid (125 mg, 92%). Spectral data of product **5** matched those previously reported.^11^

#### 4.4.2 2-((5-Bromopyridin-2-yl)­thio)-1-(1-ethoxyvinyl)­pyridin-1-ium Trifluoromethanesulfonate (**6**)

##### 4.4.2.1 Method 1

General S_N_Ar procedure was followed using 5-bromo-2-mercaptopyridine (59.4 mg, 0.313 mmol) as the nucleophile. Purification by automated column chromatography with a Teledyne ISCO CombiFlash system (0–70% chloroform/isopropyl alcohol gradient) yielded product **6** as a yellow-brown amorphous solid (132 mg, 86%). ^1^H NMR (500 MHz, Acetone-d_6_) δ 9.08 (ddd, *J* = 6.1, 1.7, 0.7 Hz, 1H), 8.61 (dd, *J* = 2.4, 0.8 Hz, 1H), 8.56–8.47 (m, 1H), 8.08 (dd, *J* = 8.4, 2.5 Hz, 1H), 8.04–7.99 (m, 2H), 7.73 (dd, *J* = 8.4, 0.7 Hz, 1H), 4.96 (d, *J* = 5.0 Hz, 1H), 4.91 (d, *J* = 5.0 Hz, 1H), 4.16 (q, *J* = 7.0 Hz, 2H), 1.28 (t, *J* = 7.0 Hz, 3H); ^13^C NMR (126 MHz, Acetone-d_6_) δ 156.3, 153.6, 152.4, 149.7, 147.3, 146.8, 141.8, 130.8, 130.2, 125.7, 122.0, 121.3 (q, *J* = 322 Hz, *C*F_3_), 87.5, 67.4, 13.3; LRMS-ES+ *m*/*z* (relative intensity) 337.0 (C_14_H_14_BrN_2_OS M+, 98 (Br-79 isotope)), 339.0 (C_14_H_14_BrN_2_OS M+, 98 (Br-81 isotope)); HRMS-ES+ (C_14_H_14_BrN_2_OS) calcd 337.0010 (M+), found 337.0014.

##### 4.4.2.2 Method 2 (Larger Scale)

A 25 mL round-bottom flask was charged with 1-(1-ethoxyvinyl)-2-chloropyridinium triflate **1a** (562 mg, 1.60 mmol), 5-bromo-2-mercaptopyridine (320 mg, 1.60 mmol), and anhydrous potassium carbonate (221 mg, 1.60 mmol). The dry powders were thoroughly blended together using a spatula; a stir bar was added, and the flask was evacuated and backfilled with argon. Acetonitrile (5 mL) was added, and the resulting mixture was stirred for 18 h at room temperature. To remove the potassium carbonate, the reaction mixture was filtered through a medium-porosity glass fritted funnel, washing with acetone (50 mL). The filtrate was concentrated to produce a reddish-brown oil that was purified by automated column chromatography with a Teledyne ISCO CombiFlash system (between 0 and 100% chloroform/isopropanol gradient; 24 g RediSep Gold prepacked silica gel cartridge) to yield the corresponding 2-thiopyridinium triflate S_N_Ar product **6** as a light-yellow oil (663 mg, 85%).

#### 4.4.3 1-(1-Ethoxyvinyl)-2-((5-methoxybenzo­[*d*]­oxazol-2-yl)­thio)­pyridin-1-ium Trifluoromethanesulfonate (**7**)

General S_N_Ar procedure was followed using 5-methoxybenzoxazole-2-thiol (56 mg, 0.30 mmol) as the nucleophile. Purification by automated column chromatography with a Teledyne ISCO CombiFlash system (0–100% chloroform/isopropyl alcohol gradient) yielded product **7** as a clear residue (14 mg, 10%). ^1^H NMR (500 MHz, Acetone-d_6_) δ 9.28 (dd, *J* = 6.2, 1.6 Hz, 1H), 8.71 (ddd, *J* = 8.4, 7.8, 1.6 Hz, 1H), 8.37 (dd, *J* = 8.3, 1.3 Hz, 1H), 8.22 (ddd, *J* = 7.6, 6.2, 1.3 Hz, 1H), 7.50 (d, *J* = 9.0 Hz, 1H), 7.20 (d, *J* = 2.6 Hz, 1H), 7.00 (dd, *J* = 9.0, 2.6 Hz, 1H), 5.09 (d, *J* = 5.1 Hz, 1H), 5.01 (d, *J* = 5.2 Hz, 1H), 4.22 (q, *J* = 7.0 Hz, 2H), 3.76 (s, 3H), 1.29 (t, *J* = 7.0 Hz, 3H); ^13^C NMR (126 MHz, Acetone-d_6_) δ 158.3, 154.6, 153.4, 148.5, 147.7, 147.2, 142.4, 140.9, 131.7, 127.2, 121.3 (app q, *J* = 321.4 Hz, *C*F_3_), 115.9, 111.4, 102.8, 87.9, 67.7, 55.6, 13.2; LRMS-ES+ *m*/*z* (relative intensity) 329.1 (C_17_H_17_N_2_O_3_S M+, 100); HRMS-ES+ (C_17_H_17_N_2_O_3_S) calcd 329.0960 (M+), found 329.0959.

#### 4.4.4 1-(1-Ethoxyvinyl)-2-(propylthio)­pyridin-1-ium Trifluoromethanesulfonate (**8**)

General S_N_Ar procedure was followed using 1-propanethiol (28 μL, 0.30 mmol) as the nucleophile. Purification by automated column chromatography with a Teledyne ISCO CombiFlash system (0–70% chloroform/isopropyl alcohol gradient) yielded product **8** as an off-white amorphous solid (109 mg, 98%). ^1^H NMR (500 MHz, Acetone-d_6_) δ 8.86 (dd, *J* = 6.3, 1.6 Hz, 1H), 8.47 (ddd, *J* = 9.0, 7.5, 1.6 Hz, 1H), 8.19 (d, *J* = 8.3 Hz, 1H), 7.81 (ddd, *J* = 7.5, 6.2, 1.2 Hz, 1H), 4.85 (ABq, *J* = 10.0, 4.9 Hz, 2H), 4.12 (q, *J* = 7.0 Hz, 2H), 3.34 (t, *J* = 7.3 Hz, 2H), 1.72 (h, *J* = 7.3 Hz, 2H), 1.28 (t, *J* = 7.0 Hz, 3H), 0.96 (t, *J* = 7.4 Hz, 3H); ^13^C NMR (126 MHz, Acetone-d_6_) δ 161.2, 153.3, 146.1 (presumed to be two overlapping peaks; cf. compound **9**), 126.2, 122.7, 121.4 (q, *J* = 321.7 Hz, *C*F_3_), 87.2, 67.1, 34.6, 21.3, 13.3, 12.4; LRMS-ES+ *m*/*z* (relative intensity) 224.1 (C_12_H_18_NOS M+, 100); HRMS-ES+ (C_12_H_18_NOS) calcd 224.1109 (M+), found 224.1110.

#### 4.4.5 1-(1-Ethoxyvinyl)-2-(pentylthio)­pyridin-1-ium Trifluoromethanesulfonate (**9**)

General S_N_Ar procedure was followed using 1-pentanethiol (38 μL, 0.30 mmol) as the nucleophile. Purification by automated column chromatography with a Teledyne ISCO CombiFlash system (0–70% chloroform/isopropyl alcohol gradient) yielded product **9** as a pale yellow amorphous solid (114 mg, 95%). ^1^H NMR (500 MHz, Acetone-d_6_) δ 8.85 (dd, *J* = 6.3, 1.6 Hz, 1H), 8.47 (ddd, *J* = 9.0, 7.5, 1.6 Hz, 1H), 8.19 (d, *J* = 8.7 Hz, 1H), 7.81 (ddd, *J* = 7.5, 6.3, 1.2 Hz, 1H), 4.85 (ABq, *J* = 11.6, 4.9 Hz, 2H), 4.12 (q, *J* = 7.0 Hz, 2H), 3.36 (t, *J* = 7.4 Hz, 2H), 1.70 (p, *J* = 7.5 Hz, 2H), 1.41–1.34 (m, 2H), 1.31–1.23 (m, 5H), 0.77 (t, *J* = 7.3 Hz, 3H); ^13^C NMR (126 MHz, Acetone-d_6_) δ 161.2, 153.3, 146.1, 146.0, 126.2, 122.6, 121.4 (q, *J* = 321.7 Hz, *C*F_3_), 87.2, 67.1, 32.9, 30.5, 27.4, 21.8, 13.28, 13.26; LRMS-ES+ *m*/*z* (relative intensity) 252.1 (C_14_H_22_NOS M+, 100); HRMS-ES+ (C_14_H_22_NOS) calcd 252.1422 (M+), found 252.1420.

#### 4.4.6 1-(1-Ethoxyvinyl)-2-(isopropylthio)­pyridin-1-ium Trifluoromethanesulfonate (**10**)

##### 4.4.6.1 Method 1

General S_N_Ar procedure was followed using 2-propanethiol (29 μL, 0.30 mmol) as the nucleophile. Purification by automated column chromatography with a Teledyne ISCO CombiFlash system (0–70% chloroform/isopropyl alcohol gradient) yielded product **10** as a light brown amorphous solid (67 mg, 60%). ^1^H NMR (500 MHz, CDCl_3_) δ 8.72 (ddd, *J* = 6.4, 1.7, 0.6 Hz, 1H), 8.53 (ddd, *J* = 9.1, 7.6, 1.6 Hz, 1H), 8.21–8.14 (m, 1H), 7.82 (ddd, *J* = 7.6, 6.3, 1.2 Hz, 1H), 4.72 (q, *J* = 5.2 Hz, 2H), 4.14 (q, *J* = 7.0 Hz, 2H), 4.04 (hept, *J* = 6.6 Hz, 1H), 1.51 (d, *J* = 6.7 Hz, 6H), 1.43 (t, *J* = 7.0 Hz, 3H); ^13^C NMR (126 MHz, CDCl_3_) δ 160.8, 152.9, 146.2, 145.7, 126.7, 122.7, 120.8 (q, *J* = 321 Hz, *C*F_3_), 87.3, 67.2, 39.5, 22.4, 13.8; LRMS-ES+ *m*/*z* (relative intensity) 224.1 (C_12_H_18_NOS M+, 100); HRMS-ES+ (C_12_H_18_NOS) calcd 294.1109 (M+), found 294.1111.

##### 4.4.6.2 Method 2

General S_N_Ar procedure was modified to use sodium 2-propanethiolate (34 mg, 90% purity, 0.30 mmol) as a nucleophile instead of 2-propanethiol while also omitting the addition of potassium carbonate. Purification by automated column chromatography with a Teledyne ISCO CombiFlash system (0–70% chloroform/isopropyl alcohol gradient) yielded product **10** as an off-white amorphous solid (90 mg, 80%).

#### 4.4.7 2-(Tert-butylthio)-1-(1-ethoxyvinyl)­pyridin-1-ium Trifluoromethanesulfonate (**11**)

General S_N_Ar procedure was followed using 2-methylpropane-2-thiol (33 μL, 0.30 mmol) as the nucleophile. Purification by automated column chromatography with a Teledyne ISCO CombiFlash system (0–70% chloroform/isopropanol gradient) yielded product **11** as a tan amorphous solid (106 mg, 91%). ^1^H NMR (500 MHz, Acetone-d_6_) δ 9.04 (dd, *J* = 6.2, 1.7 Hz, 1H), 8.63 (td, *J* = 8.0, 1.7 Hz, 1H), 8.42 (dd, *J* = 8.4, 1.3 Hz, 1H), 8.02 (ddd, *J* = 7.6, 6.2, 1.3 Hz, 1H), 4.82 (ABq, *J* = 9.9, 4.8 Hz, 2H), 4.12 (q, *J* = 7.0 Hz, 2H), 1.49 (s, 9H), 1.28 (t, *J* = 7.0 Hz, 3H); ^13^C NMR (126 MHz, Acetone-d_6_) δ 156.1, 154.1, 147.8, 147.1, 132.5, 125.5, 121.4 (q, *J* = 321.8 Hz, *C*F_3_), 87.1, 67.0, 53.5, 30.5, 13.4; LRMS-ES+ *m*/*z* (relative intensity) 238.1 (C_13_H_20_NOS M+, 45); HRMS-ES+ (C_13_H_20_NOS) calcd 238.1266 (M+), found 238.1262.

#### 4.4.8 2-(Cyclohexylthio)-1-(1-ethoxyvinyl)­pyridin-1-ium Trifluoromethanesulfonate (**12**)

General S_N_Ar procedure was followed using cyclohexanethiol (38 μL, 0.30 mmol) as the nucleophile. Purification by automated column chromatography with a Teledyne ISCO CombiFlash system (0–70% chloroform/isopropanol gradient) yielded product **12** as a pale yellow, amorphous solid (115 mg, 93%). ^1^H NMR (500 MHz, Acetone-d_6_) δ 8.86 (dd, *J* = 6.2, 1.5 Hz, 1H), 8.48 (ddd, *J* = 9.1, 7.7, 1.6 Hz, 1H), 8.27 (d, *J* = 8.8 Hz, 1H), 7.81 (ddd, *J* = 7.6, 6.3, 1.2 Hz, 1H), 4.83 (ABq, *J* = 9.6, 4.9 Hz, 2H), 4.11 (q, *J* = 7.0 Hz, 2H), 3.92 (dp, *J* = 10.0, 3.8 Hz, 1H), 2.07–2.01 (m, 2H), 1.69–1.62 (m, 2H), 1.56–1.38 (m, 6H), 1.28 (t, *J* = 7.0 Hz, 3H); ^13^C NMR (126 MHz, Acetone-d_6_) δ 160.0, 153.4, 146.3, 146.2, 127.1, 122.9, 121.4 (q, *J* = 321.7 Hz, *C*F_3_), 87.2, 67.08, 46.6, 32.2, 25.0, 24.9, 13.3; LRMS-ES+ *m*/*z* (relative intensity) 264.1 (C_15_H_22_NOS M+, 100); HRMS-ES+ (C_15_H_22_NOS) calcd 264.1442 (M+), found 264.1442.

#### 4.4.9 1-(1-Ethoxyvinyl)-2-((4-methoxybenzyl)­thio)­pyridin-1-ium Trifluoromethanesulfonate (**13**)

General S_N_Ar procedure was followed using 4-methoxybenzyl mercaptan (44.8 mg, 0.285 mmol) as the nucleophile. Purification by automated column chromatography with a Teledyne ISCO CombiFlash system (0–70% chloroform/isopropanol gradient) yielded product **13** as a light brown amorphous solid (94 mg, 73%). ^1^H NMR (500 MHz, Acetone-d_6_) δ 8.86 (dd, *J* = 6.3, 1.6 Hz, 1H), 8.48 (ddd, *J* = 8.9, 7.5, 1.6 Hz, 1H), 8.25 (dt, *J* = 8.6, 0.9 Hz, 1H), 7.82 (ddd, *J* = 7.6, 6.2, 1.2 Hz, 1H), 7.35 (d, *J* = 8.7 Hz, 2H), 6.82 (d, *J* = 8.7 Hz, 2H), 4.82 (s, 2H), 4.62 (s, 2H), 4.08 (q, *J* = 7.0 Hz, 2H), 3.66 (s, 3H), 1.25 (t, *J* = 7.0 Hz, 3H); ^13^C NMR (126 MHz, Acetone-d_6_) δ 160.4, 160.0, 153.1, 146.1, 145.9, 130.8, 126.7, 125.0, 123.0, 121.5 (q, *J* = 321.9 Hz, *C*F_3_), 114.4, 87.2, 67.1, 54.8, 37.1, 13.3; LRMS-ES+ *m*/*z* (relative intensity) 302.1 (C_17_H_20_NO_2_S M+, 100); HRMS-ES+ (C_17_H_20_NO_2_S) calcd 302.1215 (M+), found 302.1218.

#### 4.4.10 2-(((3*S*,8*S*,9*S*,10*R*,13*R*,17*R*)-10,13-Dimethyl-17-((*R*)-6-methylheptan-2-yl)-2,3,4,7,8,9,10,11,12,13,14,15,16,17-tetradecahydro-1*H*-cyclopenta­[*a*]­phenanthren-3-yl)­thio)-1-(1-ethoxyvinyl)­pyridin-1-ium trifluoromethanesulfonate (**14**)

General S_N_Ar procedure was followed using thiocholesterol (121 mg, 0.300 mmol) as the nucleophile. Purification by automated column chromatography with a Teledyne ISCO CombiFlash system (0–70% chloroform/isopropanol gradient) yielded product **14** as an off-white amorphous solid (102 mg, 48%). ^1^H NMR (500 MHz, Acetone-d_6_) δ 8.89 (dd, *J* = 6.3, 1.6 Hz, 1H), 8.50 (ddd, *J* = 9.0, 7.6, 1.7 Hz, 1H), 8.22 (dd, *J* = 8.6, 1.1 Hz, 1H), 7.84 (ddd, *J* = 7.6, 6.2, 1.2 Hz, 1H), 5.35 (dt, *J* = 5.4, 2.0 Hz, 1H), 4.85 (ABq, *J* = 7.7, 4.9 Hz, 2H), 4.12 (q, *J* = 7.0 Hz, 2H), 3.69 (dddd, *J* = 15.6, 12.1, 7.1, 4.0 Hz, 1H), 2.48–2.36 (m, 2H), 2.04–1.98 (m, 1H), 1.95–1.85 (m, 3H), 1.78–1.67 (m, 2H), 1.53–1.34 (m, 6H), 1.31–1.25 (m, 6H), 1.24–1.14 (m, 2H), 1.12–0.97 (m, 6H), 0.96 (s, 3H), 0.94–0.88 (m, 3H), 0.83 (d, *J* = 6.5 Hz, 3H), 0.75 (dd, *J* = 6.6, 1.8 Hz, 6H), 0.60 (s, 3H); ^13^C NMR (126 MHz, Acetone-d_6_) δ 159.7, 153.4, 146.3, 140.0, 127.1, 123.1, 122.6, 121.4 (q, *J* = 321.7 Hz, *C*F_3_), 87.2, 67.1, 56.7, 56.2, 50.2, 47.6, 42.2, 39.7, 39.4, 39.0, 38.2, 36.6, 36.1, 35.7, 31.72, 31.69, 28.7, 28.1, 27.8, 24.0, 23.7, 22.3, 22.0, 20.7, 18.8, 18.3, 13.3, 11.4; LRMS-ES+ *m*/*z* (relative intensity) 550.4 (C_36_H_56_NOS M+, 100); HRMS-ES+ (C_36_H_56_NOS) calcd 550.4083 (M+), found 550.4079.

#### 4.4.11 1-(1-Ethoxyvinyl)-2-((3-hydroxypropyl)­thio)­pyridin-1-ium Trifluoromethanesulfonate (**15**)

General S_N_Ar procedure was followed using 3-mercapto-1-propanol (27 μL, 0.30 mmol) as the nucleophile. Purification by automated column chromatography with a Teledyne ISCO CombiFlash system (0–70% chloroform/isopropanol gradient) yielded product **15** as a pale yellow, amorphous solid (94 mg, 79%). ^1^H NMR (500 MHz, Acetone-d_6_) δ 8.85 (dd, *J* = 6.3, 1.6 Hz, 1H), 8.47 (ddd, *J* = 9.0, 7.5, 1.6 Hz, 1H), 8.20 (d, *J* = 8.5 Hz, 1H), 7.81 (ddd, *J* = 7.5, 6.1, 1.2 Hz, 1H), 4.85 (ABq, *J* = 8.4, 4.9 Hz, 2H), 4.12 (q, *J* = 7.0 Hz, 2H), 3.90 (t, *J* = 5.3 Hz, 1H), 3.58 (q, *J* = 5.6 Hz, 2H), 3.43 (t, *J* = 7.3 Hz, 2H), 1.88 (tt, *J* = 7.4, 5.9 Hz, 2H), 1.28 (t, *J* = 7.0 Hz, 3H); ^13^C NMR (126 MHz, Acetone-d_6_) δ 161.3, 153.3, 146.0, 146.0, 126.0, 122.6, 121.3 (q, *J* = 321.4 Hz, *C*F_3_), 87.2, 67.1, 59.4, 30.9, 29.8, 13.3; LRMS-ES+ *m*/*z* (relative intensity) 240.1 (C_12_H_18_NO_2_S M+, 100); HRMS-ES+ (C_12_H_18_NO_2_S) calcd 240.1058 (M+), found 240.1057.

#### 4.4.12 1-(1-Ethoxyvinyl)-2-((2-(pyrazin-2-yl)­ethyl)­thio)­pyridin-1-ium Trifluoromethanesulfonate (**16**)

General S_N_Ar procedure was followed using 2-(pyrazin-2-yl)­ethane-1-thiol (37 μL, 0.29 mmol) as the nucleophile. Purification by automated column chromatography with a Teledyne ISCO CombiFlash system (0–100% chloroform/isopropanol gradient) yielded product **16** as an off-white amorphous solid (119 mg, 91%). ^1^H NMR (500 MHz, Acetone-d_6_) δ 8.87 (dd, *J* = 6.3, 1.6 Hz, 1H), 8.53–8.48 (m, 2H), 8.44 (dd, *J* = 2.6, 1.5 Hz, 1H), 8.37 (d, *J* = 2.6 Hz, 1H), 8.30 (dd, *J* = 8.8, 1.2 Hz, 1H), 7.84 (ddd, *J* = 7.6, 6.3, 1.2 Hz, 1H), 4.81 (ABq, *J* = 6.8, 4.9 Hz, 2H), 4.06 (q, *J* = 7.0 Hz, 2H), 3.82 (t, *J* = 7.0 Hz, 2H), 3.27 (t, *J* = 7.0 Hz, 2H), 1.22 (t, *J* = 7.0 Hz, 3H); ^13^C NMR (126 MHz, Acetone-d_6_) δ 160.5, 154.0, 153.3, 146.2, 146.2, 145.2, 144.2, 143.3, 126.4, 123.0, 121.4 (q, *J* = 321.7 Hz, *C*F_3_), 87.3, 67.1, 32.9, 31.6, 13.2; LRMS-ES+ *m*/*z* (relative intensity) 288.1 (C_15_H_18_N_3_OS M+, 100); HRMS-ES+ (C_15_H_18_N_3_OS) calcd 288.1171 (M+), found 288.1169.

#### 4.4.13 1-(1-Ethoxyvinyl)-2-(phenylthio)­pyridin-1-ium Trifluoromethanesulfonate (**18**)

##### 4.4.13.1 Method 1

General S_N_Ar procedure was followed using thiophenol (42.4 mg, 0.307 mmol) as the nucleophile. Purification by automated column chromatography with a Teledyne ISCO CombiFlash system (0–70% chloroform/isopropanol gradient) yielded product **18** as an off-white amorphous solid (65 mg, 52%). ^1^H NMR (500 MHz, Acetone-d_6_) δ 9.08 (ddd, *J* = 6.3, 1.6, 0.7 Hz, 1H), 8.49 (ddd, *J* = 8.9, 7.6, 1.6 Hz, 1H), 7.97 (ddd, *J* = 7.6, 6.2, 1.3 Hz, 1H), 7.86–7.79 (m, 2H), 7.77–7.72 (m, 1H), 7.72–7.66 (m, 2H), 7.45 (dt, *J* = 8.6, 0.9 Hz, 1H), 5.15 (d, *J* = 4.9 Hz, 1H), 5.10 (d, *J* = 4.9 Hz, 1H), 4.34 (q, *J* = 7.0 Hz, 2H), 1.49 (t, *J* = 7.0 Hz, 3H); ^13^C NMR (126 MHz, Acetone-d_6_) δ 161.7, 153.2, 146.5, 145.8, 135.9, 132.1, 131.3, 126.7, 125.2, 123.6, 121.3 (q, *J* = 322 Hz, *C*F_3_), 87.56, 67.33, 13.4; LRMS-ES+ *m*/*z* (relative intensity) 258.1 (C_15_H_16_NOS M+, 100); HRMS-ES+ (C_15_H_16_NOS) calcd 258.0953 (M+), found 258.0954.

##### 4.4.13.2 Method 2

General S_N_Ar procedure was modified to use sodium thiophenolate (40 mg, 0.30 mmol) as a nucleophile instead of thiophenol while omitting the addition of potassium carbonate. Purification by automated column chromatography with a Teledyne ISCO CombiFlash system (0–70% chloroform/isopropyl alcohol gradient) yielded product **18** as an off-white amorphous solid (108 mg, 87%).

#### 4.4.14 2-((4-Bromophenyl)­thio)-1-(1-ethoxyvinyl)­pyridin-1-ium Trifluoromethanesulfonate (**19**)

General S_N_Ar procedure was followed using 4-bromothiophenol (64 mg, 0.32 mmol) as the nucleophile. Purification by automated column chromatography with a Teledyne ISCO CombiFlash system (0–70% chloroform/isopropyl alcohol gradient) yielded product **19** as a white amorphous solid (130 mg, 89%). ^1^H NMR (500 MHz, Acetone-d_6_) δ 8.96 (dd, *J* = 6.2, 1.6 Hz, 1H), 8.38 (ddd, *J* = 9.0, 7.6, 1.6 Hz, 1H), 7.87 (ddd, *J* = 7.6, 6.3, 1.3 Hz, 1H), 7.75–7.70 (m, 2H), 7.66–7.62 (m, 2H), 7.50–7.44 (m, 1H), 5.01 (d, *J* = 4.9 Hz, 1H), 4.97 (d, *J* = 4.8 Hz, 1H), 4.21 (q, *J* = 7.0 Hz, 2H), 1.35 (t, *J* = 7.0 Hz, 3H); ^13^C NMR (126 MHz, Acetone-d_6_) δ 161.0, 153.3, 146.7, 145.9, 137.7, 134.3, 127.2, 126.4, 125.3, 123.8, 121.5 (q, *J* = 322.1 Hz, *C*F_3_), 87.6, 67.4, 13.30; LRMS-ES+ *m*/*z* (relative intensity) 336.0 (C_15_H_15_BrNOS M+, 73 (Br-79 isotope)), 338.0 (C_15_H_15_BrNOS M+, 75 (Br-81 isotope)); HRMS-ES+ (C_15_H_15_BrNOS) calcd 336.0058 (M+), found 336.0051.

#### 4.4.15 1-(1-Ethoxyvinyl)-2-((4-methoxyphenyl)­thio)­pyridin-1-ium Trifluoromethanesulfonate (**20**)

General S_N_Ar procedure was followed using 4-methoxythiophenol (38 μL, 0.50 mmol) as the nucleophile. Purification by automated column chromatography with a Teledyne ISCO CombiFlash system (0–70% chloroform/isopropyl alcohol gradient) yielded product **20** as a white amorphous solid (122 mg, 91%). ^1^H NMR (500 MHz, Acetone-d_6_) δ 8.88 (dd, *J* = 6.3, 1.6 Hz, 1H), 8.32 (ddd, *J* = 8.9, 7.5, 1.6 Hz, 1H), 7.78 (ddd, *J* = 7.5, 6.3, 1.2 Hz, 1H), 7.57 (d, *J* = 8.8 Hz, 2H), 7.27 (dd, *J* = 8.7, 1.2 Hz, 1H), 7.08 (d, *J* = 8.8 Hz, 2H), 4.97 (d, *J* = 4.9 Hz, 1H), 4.94 (d, *J* = 5.0 Hz, 1H), 4.18 (q, *J* = 7.0 Hz, 2H), 3.78 (s, 3H), 1.34 (t, *J* = 7.0 Hz, 3H); ^13^C NMR (126 MHz, Acetone-d_6_) δ 162.9, 162.8, 153.1, 146.2, 145.6, 137.9, 126.3, 123.2, 121.4 (q, *J* = 321.8 Hz, *C*F_3_), 116.8, 115.2, 87.5, 67.3, 55.4, 13.4; LRMS-ES+ *m*/*z* (relative intensity) 288.1 (C_16_H_18_NO_2_S M+, 100); HRMS-ES+ (C_16_H_18_NO_2_S) calcd 288.1058 (M+), found 288.1053.

#### 4.4.16 2-([1,1′-Biphenyl]-4-ylthio)-1-(1-ethoxyvinyl)­pyridin-1-ium Trifluoromethanesulfonate (**21**)

General S_N_Ar procedure was followed using biphenyl-4-thiol (58 μL, 0.30 mmol) as the nucleophile. Purification by automated column chromatography with a Teledyne ISCO CombiFlash system (0–70% chloroform/isopropyl alcohol gradient) yielded product **21** as a light tan amorphous solid (100 mg, 69%). ^1^H NMR (500 MHz, Acetone-d_6_) δ 9.08 (dd, *J* = 6.3, 1.5 Hz, 1H), 8.49 (ddd, *J* = 8.9, 7.5, 1.6 Hz, 1H), 8.01–7.96 (m, 3H), 7.92–7.87 (m, 2H), 7.81–7.76 (m, 2H), 7.60–7.51 (m, 3H), 7.50–7.43 (m, 1H), 5.17 (d, *J* = 4.9 Hz, 1H), 5.11 (d, *J* = 4.8 Hz, 1H), 4.36 (q, *J* = 7.0 Hz, 2H), 1.51 (t, *J* = 7.0 Hz, 3H); ^13^C NMR (126 MHz, Acetone-d_6_) δ 161.7, 153.2, 146.5, 145.8, 144.5, 139.0, 136.5, 129.4, 129.2, 128.6, 127.1, 126.9, 124.4, 123.6, 121.3 (q, *J* = 321.5 Hz, *C*F_3_), 87.6, 67.4, 13.4; LRMS-ES+ *m*/*z* (relative intensity) 334.1 (C_21_H_20_NOS M+, 100); HRMS-ES+ (C_21_H_20_NOS) calcd 334.1266 (M+), found 334.1261.

#### 4.4.17 2-((2-Bromophenyl)­thio)-1-(1-ethoxyvinyl)­pyridin-1-ium Trifluoromethanesulfonate (**22**)

General S_N_Ar procedure was followed using 2-bromothiophenol (37 μL, 0.30 mmol) as the nucleophile. Purification by automated column chromatography with a Teledyne ISCO CombiFlash system (0–70% chloroform/isopropyl alcohol gradient) yielded product **22** as a white amorphous solid (43 mg, 29%). ^1^H NMR (500 MHz, Acetone-d_6_) δ 9.03 (dd, *J* = 6.2, 1.5 Hz, 1H), 8.42 (ddd, *J* = 9.0, 7.6, 1.6 Hz, 1H), 7.93 (ddd, *J* = 7.5, 6.2, 1.3 Hz, 1H), 7.90–7.86 (m, 1H), 7.86–7.83 (m, 1H), 7.55 (pd, *J* = 7.5, 1.8 Hz, 2H), 7.37 (dt, *J* = 8.5, 0.9 Hz, 1H), 5.05 (d, *J* = 5.0 Hz, 1H), 4.99 (d, *J* = 5.0 Hz, 1H), 4.23 (q, *J* = 7.0 Hz, 2H), 1.36 (t, *J* = 7.0 Hz, 3H); ^13^C NMR (126 MHz, Acetone-d_6_) δ 159.5, 153.3, 147.0, 146.2, 138.6, 135.1, 134.1, 130.2, 129.8, 127.3, 127.0, 124.2, 121.2 (q, *J* = 321.2 Hz, *C*F_3_), 87.5, 67.5, 13.3; LRMS-ES+ *m*/*z* (relative intensity) 336.0 (C_15_H_15_BrNOS M+, 98 (Br-79 isotope)), 338.0 (C_15_H_15_BrNOS M+, 100 (Br-79 isotope)); HRMS-ES+ (C_15_H_15_BrNOS) calcd 336.0058 (M+), found 336.0057.

#### 4.4.18 1-(1-Ethoxyvinyl)-2-((2-(methoxycarbonyl)­phenyl)­thio)­pyridin-1-ium Trifluoromethanesulfonate (**23**)

General S_N_Ar procedure was followed using methyl thiosalicylate (42 μL, 0.30 mmol) as the nucleophile. Purification by automated column chromatography with a Teledyne ISCO CombiFlash system (0–70% chloroform/isopropyl alcohol gradient) yielded product **23** as a clear residue (76 mg, 55%). ^1^H NMR (500 MHz, Acetone-d_6_) δ 9.00 (ddd, *J* = 6.2, 1.5, 0.6 Hz, 1H), 8.39 (ddd, *J* = 8.7, 7.6, 1.6 Hz, 1H), 8.02–7.97 (m, 1H), 7.92 (ddd, *J* = 7.6, 6.2, 1.3 Hz, 1H), 7.79–7.73 (m, 1H), 7.70–7.65 (m, 2H), 7.54 (ddd, *J* = 8.5, 1.3, 0.6 Hz, 1H), 4.99 (d, *J* = 4.9 Hz, 1H), 4.95 (d, *J* = 4.9 Hz, 1H), 4.20 (q, *J* = 7.0 Hz, 2H), 3.71 (s, 3H), 1.33 (t, *J* = 7.0 Hz, 3H); ^13^C NMR (126 MHz, Acetone-d_6_) δ 165.8, 160.8, 153.5, 146.5, 145.8, 137.2, 134.5, 133.9, 132.1, 131.8, 128.8, 127.3, 124.2, 121.5 (app q, *J* = 322.1 Hz, *C*F_3_), 87.4, 67.4, 52.3, 13.3; LRMS-ES+ *m*/*z* (relative intensity) 316.1 (C_17_H_18_NO_3_S M+, 100); HRMS-ES+ (C_17_H_18_NO_3_S) calcd 316.1007 (M+), found 316.1007.

#### 4.4.19 2-((3,5-Bis­(trifluoromethyl)­phenyl)­thio)-1-(1-ethoxyvinyl)­pyridin-1-ium Tifluoromethanesulfonate (**25**)

General S_N_Ar procedure was followed using 3,5-bis­(trifluoromethyl)­benzenethiol (52 μL, 0.30 mmol) as the nucleophile. Purification by automated column chromatography with a Teledyne ISCO CombiFlash system (0–70% chloroform/isopropanol gradient) yielded product **25** as an off-white amorphous solid (83 mg, 51%) ^1^H NMR (500 MHz, Acetone-d_6_) δ 9.03 (ddd, *J* = 6.3, 1.6, 0.6 Hz, 1H), 8.43–8.37 (m, 3H), 8.21 (dt, *J* = 1.5, 0.8 Hz, 1H), 7.92 (ddd, *J* = 7.6, 6.2, 1.2 Hz, 1H), 7.73–7.68 (m, 1H), 5.04 (d, *J* = 5.0 Hz, 1H), 4.99 (d, *J* = 5.0 Hz, 1H), 4.21 (q, *J* = 7.0 Hz, 2H), 1.35 (t, *J* = 7.0 Hz, 3H); ^13^C NMR (126 MHz, Acetone-d_6_) δ 159.2, 153.3, 147.1, 146.3, 130.5, 136.3 (q, *J* = 3.9 Hz, ^3^ *C*F on benzene ring), 133.4 (q, *J* = 34.1 Hz, ^2^ *C*F on benzene ring), 125.5 (app sept, *J* = 3.8 Hz, ^3^ *C*F on benzene ring), 128.3, 124.7, 122.9 (q, *J* = 272.6 Hz, benzyl *C*F_3_), 121.2 (q, *J* = 321.3 Hz, triflate *C*F_3_), 87.8, 67.5, 13.3; LRMS-ES+ *m*/*z* (relative intensity) 394.1 (C_17_H_14_F_6_NOS M+, 100); HRMS-ES+ (C_17_H_14_F_6_NOS) calcd 394.0700 (M+), found 394.0694.

#### 4.4.20 2-((2,4-Difluorophenyl)­thio)-1-(1-ethoxyvinyl)­pyridin-1-ium Trifluoromethanesulfonate (**26**)

General S_N_Ar procedure was followed using 3,4-difluorothiophenol (45 mg, 0.30 mmol) as the nucleophile. Purification by automated column chromatography with a Teledyne ISCO CombiFlash system (0–70% chloroform/isopropyl alcohol gradient) yielded product **26** as a white amorphous solid (74 mg, 56%). ^1^H NMR (500 MHz, Acetone-d_6_) δ 9.14 (ddd, *J* = 6.3, 1.7, 0.6 Hz, 1H), 8.56 (ddd, *J* = 8.6, 7.6, 1.6 Hz, 1H), 8.05 (ddd, *J* = 7.6, 6.2, 1.2 Hz, 1H), 8.00 (td, *J* = 8.5, 6.2 Hz, 1H), 7.65 (dq, *J* = 8.6, 0.9 Hz, 1H), 7.45 (td, *J* = 9.1, 2.7 Hz, 1H), 7.36 (dddd, *J* = 9.0, 8.2, 2.7, 1.2 Hz, 1H), 5.19 (d, *J* = 5.0 Hz, 1H), 5.14 (d, *J* = 5.1 Hz, 1H), 4.36 (q, *J* = 7.0 Hz, 2H), 1.49 (t, *J* = 7.0 Hz, 3H); ^13^C NMR (126 MHz, Acetone-d_6_) δ 166.0 (dd, *J* = 254.7, 11.8 Hz), 163.4 (dd, *J* = 252.9, 13.4 Hz), 159.2, 153.1, 147.1, 146.3, 139.8 (d, *J* = 10.6 Hz), 126.7, 124.2, 121.2 (q, *J* = 321.1 Hz, *C*F_3_) 114.4 (dd, *J* = 22.4, 3.9 Hz), 108.9 (dd, *J* = 18.4, 4.1 Hz), 106.2 (t, *J* = 26.6 Hz), 87.8, 67.5, 13.3; LRMS-ES+ *m*/*z* (relative intensity) 294.1 (C_15_H_14_F_2_NOS M+, 100); HRMS-ES+ (C_15_H_14_F_2_NOS) calcd 294.0764 (M+), found 294.0762.

#### 4.4.21 1-(1-Ethoxyvinyl)-2-(naphthalen-2-ylthio)­pyridin-1-ium Trifluoromethanesulfonate (**27**)

General S_N_Ar procedure was followed using 2-naphthalenethiol (51 mg, 0.30 mmol) as the nucleophile. Purification by automated column chromatography with a Teledyne ISCO CombiFlash system (0–70% chloroform/isopropyl alcohol gradient) yielded product **27** as a pale yellow amorphous solid (106 mg, 76%). ^1^H NMR (500 MHz, Acetone-d_6_) δ 9.10 (ddd, *J* = 6.3, 1.7, 0.6 Hz, 1H), 8.49 (d, *J* = 1.9 Hz, 1H), 8.44 (ddd, *J* = 8.9, 7.6, 1.6 Hz, 1H), 8.19 (d, *J* = 8.6 Hz, 1H), 8.12–8.07 (m, 2H), 7.97 (ddd, *J* = 7.6, 6.3, 1.3 Hz, 1H), 7.77–7.68 (m, 3H), 7.52 (dt, *J* = 8.4, 1.1 Hz, 1H), 5.20 (d, *J* = 4.9 Hz, 1H), 5.13 (d, *J* = 4.9 Hz, 1H), 4.37 (q, *J* = 7.0 Hz, 2H), 1.52 (t, *J* = 7.0 Hz, 3H); ^13^C NMR (126 MHz, Acetone-d_6_) δ 161.6, 153.3, 146.4, 145.8, 137.0, 134.3, 134.2, 131.2, 130.6, 128.8, 128.4, 128.1, 127.7, 127.1, 123.7, 122.8, 121.4 (app q, *J* = 321.5 Hz, *C*F_3_), 87.6, 67.4, 13.4; LRMS-ES+ *m*/*z* (relative intensity) 308.1 (C_19_H_18_NOS M+, 100); HRMS-ES+ (C_19_H_18_NOS) calcd 308.1109 (M+), found 308.1104.

#### 4.4.22 1-(1-Ethoxyvinyl)-2-((4-methyl-2-oxo-2*H*-chromen-7-yl)­thio)­pyridin-1-ium Trifluoromethanesulfonate (**28**)

##### 4.4.22.1 Method 1

General S_N_Ar procedure was followed using 7-mercapto-4-methylcoumarin (64 mg, 0.32 mmol) as the nucleophile. Purification by automated column chromatography with a Teledyne ISCO CombiFlash system (0–70% chloroform/isopropyl alcohol gradient) yielded product **28** as a tan amorphous solid (146 mg, 97%). ^1^H NMR (500 MHz, Acetone-d_6_) δ 8.98 (dd, *J* = 6.2, 1.6 Hz, 1H), 8.36 (ddd, *J* = 8.8, 7.6, 1.6 Hz, 1H), 7.93–7.84 (m, 2H), 7.64–7.60 (m, 2H), 7.57 (d, *J* = 8.8 Hz, 1H), 6.33 (q, *J* = 1.4 Hz, 1H), 5.04 (d, *J* = 4.9 Hz, 1H), 4.97 (d, *J* = 4.9 Hz, 1H), 4.21 (q, *J* = 7.0 Hz, 2H), 2.40 (d, *J* = 1.3 Hz, 3H), 1.35 (t, *J* = 7.0 Hz, 3H); ^13^C NMR (126 MHz, Acetone-d_6_) δ 160.2, 158.8, 154.2, 153.3, 152.3, 146.8, 146.1, 130.7, 129.7, 127.9, 127.8, 124.2, 123.3, 122.6, 121.2 (app q, *J* = 320.9 Hz, *C*F_3_), 116.6, 87.7, 67.4, 17.7, 13.4; LRMS-ES+ *m*/*z* (relative intensity) 340.1 (C_19_H_18_NO_3_S M+, 100); HRMS-ES+ (C_19_H_18_NO_3_S) calcd 340.1007 (M+), found 340.1000.

##### 4.4.22.2 Method 2 (Larger Scale)

A 50 mL round-bottomed flask was charged with 1-(1-ethoxyvinyl)-2-chloropyridinium triflate **1a** (1.745 g, 4.968 mmol), 7-mercapto-4-methylcoumarin (985 mg, 4.97 mmol), and anhydrous potassium carbonate (687 mg, 4.97 mmol). The dry powders were thoroughly blended together using a spatula, a stir bar was added, and the flask was evacuated and backfilled with argon. Acetonitrile (17 mL) was added, and the resulting mixture was stirred for 18 h at room temperature. To remove the potassium carbonate, the reaction mixture was filtered through a medium-porosity glass fritted funnel, washing with acetone (50 mL). The filtrate was concentrated to produce 2.63 g of a brown amorphous solid that was recrystallized by dissolving in 1,2-dimethoxyethane (5 mL) in a 25 mL beaker, heating to boil, then gradually cooling to room temperature to produce a solid. The recrystallization mixture was put in a 2–5 °C refrigerator for an additional 45 min, then the product was collected by vacuum filtration using a Buchner funnel and filter paper to yield a tan amorphous solid (2.286 g, 94%).

#### 4.4.23 1-(1-Ethoxyvinyl)-2-(pyridin-2-ylthio)­pyridin-1-ium Trifluoromethanesulfonate (**29**)

General S_N_Ar procedure was followed using 2-mercaptopyridine (37 mg, 0.33 mmol) as the nucleophile. Purification by automated column chromatography with a Teledyne ISCO CombiFlash system (0–100% chloroform/isopropyl alcohol gradient) yielded product **29** as an off-white amorphous solid (108 mg, 88%). ^1^H NMR (500 MHz, Acetone-d_6_) δ 9.06 (dd, *J* = 6.3, 1.6 Hz, 1H), 8.54 (dd, *J* = 5.1, 1.9 Hz, 1H), 8.52–8.46 (m, 1H), 7.98 (ddd, *J* = 7.6, 6.1, 1.3 Hz, 1H), 7.93–7.86 (m, 2H), 7.74 (d, *J* = 7.9 Hz, 1H), 7.45 (ddd, *J* = 7.5, 4.9, 1.1 Hz, 1H), 4.96 (d, *J* = 4.9 Hz, 1H), 4.91 (d, *J* = 5.0 Hz, 1H), 4.16 (q, *J* = 7.0 Hz, 2H), 1.28 (t, *J* = 7.0 Hz, 3H); ^13^C NMR (126 MHz, Acetone-d_6_) δ 157.3, 153.6, 151.7, 150.6, 147.0, 146.6, 139.3, 130.4, 128.9, 125.3, 125.2, 121.4 (q, *J* = 321.9 Hz, *C*F_3_), 87.4, 67.4, 13.3; LRMS-ES+ *m*/*z* (relative intensity) 259.1 (C_14_H_15_N_2_OS M+, 100); HRMS-ES+ (C_14_H_15_N_2_OS) calcd 259.0905 (M+), found 259.0909.

#### 4.4.24 1-(1-Ethoxyvinyl)-2-(pyrazin-2-ylthio)­pyridin-1-ium Trifluoromethanesulfonate (**30**)

General S_N_Ar procedure was followed using 2-mercaptopyrazine (55 mg, 0.30 mmol) as the nucleophile. Purification by automated column chromatography with a Teledyne ISCO CombiFlash system (0–100% chloroform/isopropyl alcohol gradient) yielded product **30** as a clear residue (102 mg, 81%). ^1^H NMR (500 MHz, Acetone-d_6_) δ 9.14 (dd, *J* = 6.5, 1.6 Hz, 1H), 8.88 (d, *J* = 1.5 Hz, 1H), 8.64 (d, *J* = 2.4 Hz, 1H), 8.59 (dd, *J* = 2.5, 1.5 Hz, 1H), 8.58–8.52 (m, 1H), 8.06 (ddq, *J* = 6.5, 2.8, 1.3 Hz, 2H), 4.99 (d, *J* = 5.0 Hz, 1H), 4.93 (d, *J* = 5.0 Hz, 1H), 4.18 (q, *J* = 7.0 Hz, 2H), 1.28 (t, *J* = 7.0 Hz, 3H); ^13^C NMR (126 MHz, Acetone-d_6_) δ 155.4, 153.6, 148.9, 148.2, 147.5, 147.1, 146.3, 145.6, 131.2, 126.0, 121.3 (q, *J* = 321.7 Hz, *C*F_3_), 87.6, 67.4, 13.3; LRMS-ES+ *m*/*z* (relative intensity) 260.1 (C_13_H_14_N_3_OS M+, 95); HRMS-ES+ (C_13_H_14_N_3_OS) calcd 260.0858 (M+), found 260.0856.

#### 4.4.25 1-(1-Ethoxyvinyl)-2-(pyrimidin-2-ylthio)­pyridin-1-ium Trifluoromethanesulfonate (**31**)

##### 4.4.25.1 Method 1

General S_N_Ar procedure was followed using 2-mercaptopyrimidine (34 mg, 0.30 mmol) as the nucleophile. Purification by automated column chromatography with a Teledyne ISCO CombiFlash system (0–100% chloroform/isopropanol gradient) yielded product **31** as a light brown amorphous solid (122 mg, 99%). ^1^H NMR (500 MHz, Acetone-d_6_) δ 9.28 (dd, *J* = 6.1, 1.6 Hz, 1H), 8.79 (td, *J* = 8.0, 1.6 Hz, 1H), 8.65 (dd, *J* = 8.1, 1.5 Hz, 1H), 8.60 (d, *J* = 4.9 Hz, 2H), 8.26 (ddd, *J* = 7.6, 6.2, 1.4 Hz, 1H), 7.36 (t, *J* = 4.9 Hz, 1H), 4.86 (d, *J* = 4.9 Hz, 1H), 4.83 (d, *J* = 5.0 Hz, 1H), 4.10 (q, *J* = 7.0 Hz, 2H), 1.14 (t, *J* = 7.0 Hz, 3H); ^13^C NMR (126 MHz, Acetone-d_6_) δ 167.1, 159.1, 154.2, 150.2, 148.2, 147.9, 136.5, 128.1, 121.3 (q, *J* = 321.7 Hz, *C*F_3_), 120.4, 87.1, 67.3, 13.2; LRMS-ES+ *m*/*z* (relative intensity) 260.1 (C_13_H_14_N_3_OS M+, 100); HRMS-ES+ (C_13_H_14_N_3_OS) calcd 260.0858 (M+), found 260.0858.

##### 4.4.25.2 Method 2 (Larger Scale)

A 50 mL round-bottomed flask was charged with 1-(1-ethoxyvinyl)-2-chloropyridinium triflate **1a** (1.00 g, 2.85 mmol), 2-mercaptopyrimidine (326 mg, 2.85 mmol), and anhydrous potassium carbonate (393 mg, 2.85 mmol). The dry powders were thoroughly blended together using a spatula, a stir bar was added, and the flask was evacuated and backfilled with argon. Acetonitrile (10 mL) was added, and the resulting mixture was stirred for 18 h at room temperature. To remove the potassium carbonate, the reaction mixture was filtered through a medium-porosity glass fritted funnel, washing with acetone (50 mL). The filtrate was concentrated to produce a reddish-brown oil that was purified by automated column chromatography with a Teledyne ISCO CombiFlash system (between 0 and 100% chloroform/isopropanol gradient; 24 g RediSep Gold prepacked silica gel cartridge) to yield the corresponding 2-thiopyridinium triflate S_N_Ar product as a brown oil (1.15 g, 99%).

#### 4.4.26 1-(1-Ethoxyvinyl)-2-(pyridin-4-ylthio)­pyridin-1-ium Trifluoromethanesulfonate (**32**)

General S_N_Ar procedure was followed using pyridine-4-thiol (33 mg, 0.30 mmol) as the nucleophile. Purification by automated column chromatography with a Teledyne ISCO CombiFlash system (0–70% chloroform/isopropanol gradient) yielded product **32** as a clear residue (12 mg, 10%). ^1^H NMR (500 MHz, Acetone-d_6_) δ 9.08 (dd, *J* = 6.3, 1.6 Hz, 1H), 8.68–8.64 (m, 2H), 8.49 (ddd, *J* = 8.3, 7.6, 1.6 Hz, 1H), 8.00 (ddd, *J* = 7.5, 6.2, 1.2 Hz, 1H), 7.80–7.75 (m, 1H), 7.66–7.62 (m, 2H), 5.03 (d, *J* = 4.9 Hz, 1H), 4.97 (d, *J* = 5.0 Hz, 1H), 4.21 (q, *J* = 7.0 Hz, 2H), 1.33 (t, *J* = 7.0 Hz, 3H); ^13^C NMR (126 MHz, Acetone-d_6_) δ 158.0, 153.5, 151.7, 147.3, 146.6, 138.1, 129.2, 128.2, 125.4, 125.1, 121.5 (app q, *J* = 321.7 Hz, *C*F_3_), 87.6, 67.4, 13.3; LRMS-ES+ *m*/*z* (relative intensity) 259.1 (C_14_H_15_N_2_OS M+, 100); HRMS-ES+ (C_14_H_15_N_2_OS) calcd 259.0905 (M+), found 259.0902.

#### 4.4.27 2-((2,5-Dimethylfuran-3-yl)­thio)-1-(1-ethoxyvinyl)­pyridin-1-ium Trifluoromethanesulfonate (**33**)

General S_N_Ar procedure was followed using 2,5-dimethylfuran-3-thiol (41 mg, 0.30 mmol) as the nucleophile. Purification by automated column chromatography with a Teledyne ISCO CombiFlash system (0–70% chloroform/isopropanol gradient) yielded product **33** as a pale yellow, amorphous solid (63 mg, 49%). ^1^H NMR (500 MHz, CDCl_3_) δ 8.80 (dd, *J* = 6.3, 1.5 Hz, 1H), 8.38 (ddd, *J* = 8.8, 7.6, 1.6 Hz, 1H), 7.87 (ddd, *J* = 7.6, 6.3, 1.3 Hz, 1H), 7.54–7.45 (m, 1H), 6.03 (d, *J* = 1.3 Hz, 1H), 4.95 (d, *J* = 5.2 Hz, 1H), 4.82 (d, *J* = 5.2 Hz, 1H), 4.20 (q, *J* = 7.0 Hz, 2H), 2.35 (s, 3H), 2.33 (d, *J* = 1.2 Hz, 3H), 1.48 (t, *J* = 7.0 Hz, 3H); ^13^C NMR (126 MHz, CDCl_3_) δ 161.4, 158.5, 153.7, 152.6, 146.1, 145.7, 125.6, 123.6, 120.8 (q, *J* = 321 Hz, *C*F_3_), 109.3, 101.0, 87.8, 67.3, 13.9, 13.6, 11.9; LRMS-ES+ *m*/*z* (relative intensity) 276.1 (C_15_H_18_NO_2_S M+, 100); HRMS-ES+ (C_15_H_18_NO_2_S) calcd 276.1058 (M+), found 276.1062.

#### 4.4.28 2-((4,5-Dihydrothiazol-2-yl)­thio)-1-(1-ethoxyvinyl)­pyridin-1-ium Trifluoromethanesulfonate (**34**)

General S_N_Ar procedure was followed using 2-thiazoline-2-thiol (36 mg, 0.30 mmol) as the nucleophile. Purification by automated column chromatography with a Teledyne ISCO CombiFlash system (0–70% chloroform/isopropanol gradient) yielded product **34** as a white, amorphous solid (91 mg, 73%). ^1^H NMR (500 MHz, Acetone-d_6_) δ 9.23 (dd, *J* = 6.1, 1.7 Hz, 1H), 8.94 (td, *J* = 8.0, 1.7 Hz, 1H), 8.42 (dd, *J* = 8.1, 1.3 Hz, 1H), 8.30 (ddd, *J* = 7.8, 6.1, 1.4 Hz, 1H), 4.90 (d, *J* = 5.1 Hz, 1H), 4.84 (d, *J* = 5.1 Hz, 1H), 4.57 (t, *J* = 7.5 Hz, 2H), 4.20–4.11 (m, 2H), 3.73 (t, *J* = 7.5 Hz, 2H), 1.29 (t, *J* = 7.0 Hz, 3H); ^13^C NMR (126 MHz, Acetone-d_6_) δ 152.9, 151.2, 148.4, 147.4, 130.6, 128.6, 121.3 (q, *J* = 322 Hz, *C*F_3_), 86.3, 67.7, 59.2, 31.6, 13.4; LRMS-ES+ *m*/*z* (relative intensity) 267.1 (C_12_H_15_N_2_OS_2_ M+, 100); HRMS-ES+ (C_12_H_15_N_2_OS_2_) calcd 267.0626 (M+), found 267.0631.

#### 4.4.29 1-(1-Ethoxyvinyl)-2-((4-hydroxy-6-methylpyrimidin-2-yl)­thio)­pyridin-1-ium Trifluoromethanesulfonate (**36**)

General S_N_Ar procedure was followed using 6-methyl-2-thiouracil (43 mg, 0.30 mmol) as the nucleophile. Purification by automated column chromatography with a Teledyne ISCO CombiFlash system (0–70% chloroform/isopropanol gradient) yielded product **36** as a yellow, amorphous solid (120 mg, 91%). ^1^H NMR (500 MHz, CD_3_CN) δ 8.83 (ddd, *J* = 6.1, 1.6, 0.5 Hz, 1H), 8.67 (ddd, *J* = 8.2, 7.7, 1.7 Hz, 1H), 8.14 (ddd, *J* = 8.3, 1.4, 0.6 Hz, 1H), 8.09 (ddd, *J* = 7.7, 6.2, 1.4 Hz, 1H), 5.84 (q, *J* = 0.8 Hz, 1H), 4.96 (d, *J* = 4.9 Hz, 1H), 4.62 (d, *J* = 4.9 Hz, 1H), 4.07–3.91 (m, 2H), 2.20 (d, *J* = 0.8 Hz, 3H), 1.29 (t, *J* = 7.0 Hz, 3H); ^13^C NMR (126 MHz, CD_3_CN) δ 178.1, 163.4, 163.2, 152.9, 150.1, 149.7, 146.0, 132.4, 128.0, 121.8 (app q, *J* = 320.7 Hz, *C*F_3_), 102.6, 86.6, 68.1, 22.3, 13.7; LRMS-ES+ *m*/*z* (relative intensity) 318.1 (C_14_H_16_N_3_O_2_S M+, 100); HRMS-ES+ (C_14_H_16_N_3_O_2_S) calcd 290.0963 (M+), found 290.0965.

#### 4.4.30 1-(1-Ethoxyvinyl)-2-((4-hydroxy-6-propylpyrimidin-2-yl)­thio)­pyridin-1-ium Trifluoromethanesulfonate (**37**)

General S_N_Ar procedure was followed using 6-propyl-2-thiouracil (42 mg, 0.30 mmol) as the nucleophile. Purification by automated column chromatography with a Teledyne ISCO CombiFlash system (0–70% chloroform/isopropanol gradient) yielded product **37** as a light brown, amorphous solid (122 mg, 83%). ^1^H NMR (500 MHz, Acetone-d_6_) δ 11.9 (br s, 1H), 9.26 (dd, *J* = 6.2, 1.7 Hz, 1H), 8.94 (td, *J* = 8.0, 1.7 Hz, 1H), 8.38 (dd, *J* = 8.1, 1.4 Hz, 1H), 8.35 (ddd, *J* = 7.6, 6.2, 1.4 Hz, 1H), 5.91 (s, 1H), 4.78 (d, *J* = 5.1 Hz, 1H), 4.71 (d, *J* = 5.1 Hz, 1H), 3.96 (dddd, *J* = 16.8, 9.9, 7.0, 2.8 Hz, 2H), 2.47 (td, *J* = 7.3, 2.8 Hz, 2H), 1.61 (sx, *J* = 7.5 Hz, 2H), 1.18 (t, *J* = 7.0 Hz, 3H), 0.87 (t, *J* = 7.4 Hz, 3H); ^13^C NMR (126 MHz, Acetone) δ 177.3, 160.1, 158.8, 152.5, 150.9, 147.2, 146.8, 131.7, 129.0, 120.3 (q, *J* = 321.3 Hz, *C*F_3_), 102.2, 86.7, 67.5, 34.1, 20.9, 13.1, 12.7; LRMS-ES+ *m*/*z* (relative intensity) 318.1 (C_16_H_20_N_3_O_2_S M+, 100); HRMS-ES+ (C_16_H_20_N_3_O_2_S) calcd 318.1276 (M+), found 318.1278.

#### 4.4.31 2-((5-Bromopyridin-2-yl)­thio)-3-chloro-1-(1-ethoxyvinyl)­pyridin-1-ium Trifluoromethanesulfonate (**39**)

General S_N_Ar procedure was followed using 5-bromo-2-mercaptopyridine (51 mg, 0.27 mmol) as the nucleophile and freshly prepared 2,3-dichloro-1-(1-ethoxyvinyl)­pyridin-1-ium trifluoromethanesulfonate **38** ^14^ (100 mg, 0.245 mmol) as the electrophile. Purification by automated column chromatography with a Teledyne ISCO CombiFlash system (0–70% chloroform/isopropanol gradient) yielded product **39** as a brown, amorphous solid (10 mg, 8%). ^1^H NMR (500 MHz, Acetone-d_6_) δ 9.39 (dd, *J* = 6.0, 1.5 Hz, 1H), 8.96 (dd, *J* = 8.5, 1.4 Hz, 1H), 8.40–8.35 (m, 2H), 7.97 (dd, *J* = 8.5, 2.5 Hz, 1H), 7.54 (dd, *J* = 8.6, 0.7 Hz, 1H), 4.88 (d, *J* = 5.0 Hz, 1H), 4.81 (d, *J* = 5.0 Hz, 1H), 4.09 (q, *J* = 7.0 Hz, 2H), 1.11 (t, *J* = 7.0 Hz, 3H); ^13^C NMR (126 MHz, Acetone-d_6_) δ 155.1, 152.7, 151.2, 150.4, 149.2, 147.9, 143.2, 141.0, 129.3, 124.9, 121.3 (q, *J* = 321.4 Hz, *C*F_3_), 119.3, 86.8, 67.4, 13.2; LRMS-ES+ *m*/*z* (relative intensity) 371.0 (C_14_H_13_BrClN_2_OS M+, 70); HRMS-ES+ (C_14_H_13_BrClN_2_OS) calcd 370.9620 (M+), found 370.9618.

#### 4.4.32 2-((5-Bromopyridin-2-yl)­thio)-1-(1-ethoxy-3-phenylprop-1-en-1-yl)­pyridin-1-ium Trifluoromethanesulfonate (**41**)

General S_N_Ar procedure was followed using 5-bromo-2-mercaptopyridine (49 mg, 0.24 mmol) as the nucleophile, and freshly prepared 2-chloro-1-(1-ethoxy-3-phenylprop-1-en-1-yl)­pyridin-1-ium trifluoromethanesulfonate **40** ^14^ (102 mg, 0.228 mmol, ∼1:1 *E*/*Z* mixture) as the electrophile. Purification by automated column chromatography with a Teledyne ISCO CombiFlash system (0–70% chloroform/isopropanol gradient) yielded product **41** as a clear residue (65 mg, 49%, ∼1.1:1 *E*/*Z* mixture). ^1^H NMR (500 MHz, Acetone-d_6_; ∼1:1 *E*/*Z* mixture, all product peaks reported) δ 9.17–9.07 (m, 2H), 8.62 (ddd, *J* = 13.1, 2.4, 0.7 Hz, 2H), 8.56 (ddd, *J* = 8.4, 7.7, 1.6 Hz, 1H), 8.48 (ddd, *J* = 8.3, 7.7, 1.6 Hz, 1H), 8.12–8.03 (m, 4H), 8.02–7.95 (m, 2H), 7.72 (ddd, *J* = 8.4, 4.4, 0.7 Hz, 2H), 7.26–7.16 (m, 4H), 7.14–7.04 (m, 6H), 5.83 (t, *J* = 7.7 Hz, 1H), 5.51 (t, *J* = 7.7 Hz, 1H), 4.21–4.07 (m, 2H), 3.91 (q, *J* = 7.0 Hz, 2H), 3.62 (d, *J* = 7.8 Hz, 2H), 3.17 (dd, *J* = 7.7, 5.4 Hz, 2H), 1.24 (dt, *J* = 12.5, 7.0 Hz, 6H); ^13^C NMR (126 MHz, Acetone-d_6_, ∼1:1 *E*/*Z* mixture, all product peaks reported, only one signal for the triflate *C*F_3_ was observed) δ 157.30, 156.82, 152.58, 152.43, 149.82, 148.96, 147.93, 147.50, 147.26, 147.18, 146.94, 146.81, 141.78, 141.71, 138.49, 138.45, 130.90, 130.88, 130.37, 130.19, 128.66, 128.60, 128.52, 128.37, 126.56, 125.98, 125.57, 122.23, 122.03, 121.3 (q, *J* = 321.3 Hz, *C*F_3_), 116.40, 101.56, 68.46, 66.97, 31.37, 31.09, 14.22, 13.52; LRMS-ES+ *m*/*z* (relative intensity) 427.00 (C_21_H_20_BrN_2_OS M+, 95 (Br-79 isotope)), 429.0 (C_21_H_20_BrN_2_OS M+, 100 (Br-79 isotope)); HRMS-ES+ (C_21_H_20_BrN_2_OS) calcd 427.0480 (M+), found 427.0471.

#### 4.4.33 (*E*)-1-(1-Ethoxy-3-phenylprop-1-en-1-yl)-2-(pyrimidin-2-ylthio)­pyridin-1-ium Trifluoromethanesulfonate ((*E*)-**42**) and (*Z*)-1-(1-Ethoxy-3-phenylprop-1-en-1-yl)-2-(pyrimidin-2-ylthio)­pyridin-1-ium Trifluoromethanesulfonate ((*Z*)-**42**)

General S_N_Ar procedure was followed using 2-mercaptopyrimidine (28 mg, 0.25 mmol) as the nucleophile and freshly prepared 2-chloro-1-(1-ethoxy-3-phenylprop-1-en-1-yl)­pyridin-1-ium trifluoromethanesulfonate **40** ^14^ (105 mg, 0.249 mmol, ∼1:1 *E*/*Z* mixture) as the electrophile. Purification by automated column chromatography with a Teledyne ISCO CombiFlash system (0–70% chloroform/isopropanol gradient) yielded products **(** *E* **)-42** (28 mg, 23%) and **(** *Z* **)-42** (56 mg, 45%) as separable isomers and both as clear residues.

##### 4.4.33.1 Spectral Data for Product (**E**)-**42**

^1^H NMR (500 MHz, Acetone-d_6_) δ 9.37 (dd, *J* = 6.2, 1.6 Hz, 1H), 8.78 (td, *J* = 7.9, 1.6 Hz, 1H), 8.66 (dd, *J* = 8.2, 1.4 Hz, 1H), 8.57 (d, *J* = 4.8 Hz, 2H), 8.28 (ddd, *J* = 7.7, 6.1, 1.4 Hz, 1H), 7.30 (t, *J* = 4.9 Hz, 1H), 7.18–7.10 (m, 4H), 7.10–7.05 (m, 1H), 5.74 (t, *J* = 7.8 Hz, 1H), 3.86 (q, *J* = 7.0 Hz, 2H), 3.57 (d, *J* = 7.8 Hz, 2H), 1.13 (t, *J* = 7.0 Hz, 3H); ^13^C NMR (126 MHz, Acetone-d_6_) δ 167.4, 159.0, 150.5, 148.5, 148.1, 147.9, 138.4, 136.9, 128.6, 128.4, 128.1, 126.5, 121.4 (q, *J* = 321.4 Hz, *C*F_3_), 120.1, 116.3, 68.6, 31.0, 14.1; LRMS-ES+ *m*/*z* (relative intensity) 350.1 (C_20_H_20_N_3_OS M+, 12); HRMS-ES+ (C_20_H_20_N_3_OS) calcd 350.1327 (M+), found 350.1330.

##### 4.4.33.2 Spectral Data for Product (**Z**)**-42**

^1^H NMR (500 MHz, Acetone-d_6_) δ 9.30 (ddd, *J* = 6.1, 1.6, 0.6 Hz, 1H), 8.90–8.83 (m, 1H), 8.77 (dd, *J* = 8.4, 1.4 Hz, 1H), 8.58 (d, *J* = 4.9 Hz, 2H), 8.34 (ddd, *J* = 7.6, 6.1, 1.4 Hz, 1H), 7.32 (t, *J* = 4.9 Hz, 1H), 7.08–6.99 (m, 3H), 6.99–6.94 (m, 2H), 5.50 (dd, *J* = 8.4, 7.1 Hz, 1H), 4.13 (ddq, *J* = 33.3, 9.5, 7.0 Hz, 2H), 3.18 (dd, *J* = 16.3, 7.1 Hz, 1H), 3.04 (dd, *J* = 16.2, 8.3 Hz, 1H), 1.16 (t, *J* = 7.0 Hz, 3H); ^13^C NMR (126 MHz, Acetone-d_6_) δ 166.8, 159.1, 151.4, 148.8, 148.3, 148.0, 138.5, 137.0, 128.5, 128.5, 128.0, 126.5, 121.4 (q, *J* = 321.5 Hz, *C*F_3_), 120.4, 101.0, 66.9, 31.2, 13.5; LRMS-ES+ *m*/*z* (relative intensity) 350.1 (C_20_H_20_N_3_OS M+, 100); HRMS-ES+ (C_20_H_20_N_3_OS) calcd 350.1327 (M+), found 350.1328.

#### 4.4.34 2-(Isopropylthio)­pyridin-1-ium Chloride (**43**)

General procedure for the hydrolysis of the *N*-(1-ethoxyvinyl) group was followed using compound **10** (29 mg, 79 μmol) to yield product **43** as a clear residue (18 mg, 77%). ^1^H NMR (500 MHz, Acetone-d_6_) δ 8.70 (d, *J* = 5.8 Hz, 1H), 8.47 (t, *J* = 7.9 Hz, 1H), 8.08 (d, *J* = 8.3 Hz, 1H), 7.82 (t, *J* = 6.7 Hz, 1H), 4.07 (sept, *J* = 6.7 Hz, 1H), 1.37 (d, *J* = 6.5 Hz, 6H); ^13^C NMR (126 MHz, Acetone-d_6_) δ 156.3, 146.2, 142.7, 127.1, 123.2, 121.1 (app q, *J* = 320.3 Hz, *C*F_3_), 38.3, 22.0; LRMS-ES+ *m*/*z* (relative intensity) 154.1 (C_8_H_12_NS M+, 100); HRMS-ES+ (C_8_H_12_NS) calcd 154.0690 (M+), found 154.0691.

#### 4.4.35 2-(Tert*-*butylthio)­pyridin-1-ium Trifluoromethanesulfonate (**44**)

General procedure for the hydrolysis of the *N*-(1-ethoxyvinyl) group was followed using compound **11** (31 mg, 79 μmol) to yield product **44** as a clear residue (24.5 mg, 98%). ^1^H NMR (500 MHz, Acetone-d_6_) δ 9.08 (dd, *J* = 5.8, 1.6 Hz, 1H), 8.78 (td, *J* = 7.9, 1.6 Hz, 1H), 8.42 (d, *J* = 8.0 Hz, 1H), 8.28 (app t, *J* = 7.0 Hz, 1H), 1.53 (s, 9H); ^13^C NMR (126 MHz, Acetone-d_6_) δ 150.1, 147.1, 144.5, 135.1, 127.0, 121.2 (app q, *J* = 320.7 Hz, *C*F_3_), 52.1, 30.4; LRMS-ES+ *m*/*z* (relative intensity) 168.1 (C_9_H_14_NS M+, 30); HRMS-ES+ (C_9_H_14_NS) calcd 168.0847 (M+), found 168.0849.

#### 4.4.36 2-(Phenylthio)­pyridin-1-ium Chloride (**45**)

General procedure for the hydrolysis of the *N*-(1-ethoxyvinyl) group was followed using compound **18** (30 mg, 79 μmol) to yield product **45** as a clear residue (12 mg, 75%). ^1^H NMR (500 MHz, Acetone-d_6_) δ 8.69 (d, *J* = 5.6 Hz, 1H), 8.32 (t, *J* = 7.9 Hz, 1H), 7.75 (t, *J* = 6.7 Hz, 1H), 7.70–7.65 (m, 2H), 7.60–7.51 (m, 3H), 7.42 (d, *J* = 8.3 Hz, 1H); ^13^C NMR (126 MHz, Acetone-d_6_) δ 158.3, 146.0, 142.9, 135.9, 131.8, 131.1, 125.6, 125.2, 123.2; LRMS-ES+ *m*/*z* (relative intensity) 188.05 (C_11_H_10_NS M+, 100); HRMS-ES+ (C_11_H_10_NS) calcd 188.0534 (M+), found 188.0535.

#### 4.4.37 2-((5-Bromopyridin-2-yl)­thio)­pyridin-1-ium Chloride (**46**)

General procedure for the hydrolysis of the *N*-(1-ethoxyvinyl) group was followed using compound **6** (36 mg, 75 μmol) to yield product **46** as a light yellow residue (20.5 mg, 91%). ^1^H NMR (500 MHz, Acetone-d_6_) δ 8.96 (dd, *J* = 6.0, 1.6 Hz, 1H), 8.69 (d, *J* = 2.4 Hz, 1H), 8.55 (td, *J* = 8.0, 1.6 Hz, 1H), 8.14 (d, *J* = 8.3 Hz, 1H), 8.10 (dd, *J* = 8.6, 2.4 Hz, 1H), 8.00 (ddd, *J* = 7.4, 5.9, 1.1 Hz, 1H), 7.65 (d, *J* = 8.5 Hz, 1H); ^13^C NMR (126 MHz, Acetone-d_6_) δ 151.7, 150.8, 146.2, 143.5, 142.8, 141.8, 128.9, 126.6, 125.4, 120.2; LRMS-ES+ *m*/*z* (relative intensity) 267.0 (C_10_H_8_BrN_2_S M+, 98 (Br-79 isotope)), 269.0 (C_10_H_8_BrN_2_S M+, 100 (Br-81 isotope)); HRMS-ES+ (C_10_H_8_BrN_2_S) calcd 266.9592 (M+), found 266.9589.

#### 4.4.38 2-((4-Methyl-2-oxo-2*H*-chromen-7-yl)­thio)­pyridin-1-ium Chloride (**47**)

General procedure for the hydrolysis of the *N*-(1-ethoxyvinyl) group was followed using compound **28** (29 mg, 60 μmol) to yield product **47** as a clear residue (17 mg, 95%). ^1^H NMR (500 MHz, Acetone) δ 8.76–8.71 (m, 1H), 8.43–8.37 (m, 1H), 7.89–7.82 (m, 2H), 7.73 (d, *J* = 8.3 Hz, 1H), 7.64 (d, *J* = 1.7 Hz, 1H), 7.61 (dd, *J* = 8.2, 1.8 Hz, 1H), 6.33 (q, *J* = 1.3 Hz, 1H), 2.41 (d, *J* = 1.3 Hz, 3H); ^13^C NMR (126 MHz, Acetone-d_6_) δ 158.8, 156.0, 154.3, 152.1, 146.6, 142.9, 130.2, 129.2, 127.6, 127.0, 124.1, 122.9, 122.4, 116.5, 17.7; LRMS-ES+ *m*/*z* (relative intensity) 270.1 (C_15_H_12_NO_2_S M+, 100); HRMS-ES+ (C_15_H_12_NO_2_S) calcd 270.0589 (M+), found 270.0583.

#### 4.4.39 2-((4-Hydroxy-6-propylpyrimidin-2-yl)­thio)­pyridin-1-ium Trifluoromethanesulfonate (**48**)

General procedure for the hydrolysis of the *N*-(1-ethoxyvinyl) group was followed using compound **37** (26 mg, 55 μmol) to yield product **48** as a clear residue (22 mg, 99%). ^1^H NMR (500 MHz, Acetone-d_6_) δ 9.26–9.21 (m, 1H), 9.01 (td, *J* = 8.0, 1.6 Hz, 1H), 8.44–8.37 (m, 2H), 6.06 (s, 1H), 2.62 (t, *J* = 7.6 Hz, 2H), 1.77 (h, *J* = 7.4 Hz, 2H), 1.04 (t, *J* = 7.4 Hz, 3H); ^13^C NMR (126 MHz, Acetone-d_6_) δ 177.5, 160.2, 157.6, 149.4, 146.3, 143.3, 129.6, 128.0, 121.1 (app q, *J* = 320.5 Hz, *C*F_3_), 102.6, 34.0, 20.8, 12.7; LRMS-ES+ *m*/*z* (relative intensity) 248.1 (C_12_H_14_N_3_OS M+, 100); HRMS-ES+ (C_12_H_14_N_3_OS) calcd 248.0852 (M+), found 248.0853.

#### 4.4.40 1-(1-Ethoxyvinyl)-2-butoxypyridin-1-ium Trifluoromethanesulfonate (**49**) and 2-Butoxypyridin-1-ium Trifluoromethanesulfonate (**50**)

In a 0.5–2.0 mL Biotage microwave vial, 2-chloropyridinium salt **1a** (100.5 mg, 0.301 mmol) was dissolved in n-butanol (0.5 mL). The vial was capped and placed into a sonicator for 2 h, during which the water bath temperature warmed from 22 to 40 °C. Following this, the reaction mixture was concentrated in vacuo to produce a residue that was purified by automated column chromatography with a Teledyne ISCO CombiFlash system (0–70% chloroform/isopropanol gradient) to yield product **49** (44 mg, 40%) as a clear residue and product **50** (13 mg, 15%) as a pale yellow residue.

##### 4.4.40.1 Spectral Data for Product **49**

^1^H NMR (500 MHz, Acetone-d_6_) δ 8.74 (ddd, *J* = 9.2, 7.5, 1.9 Hz, 1H), 8.70 (dd, *J* = 6.4, 1.7 Hz, 1H), 7.95 (dt, *J* = 8.9, 0.9 Hz, 1H), 7.73 (ddd, *J* = 7.5, 6.3, 1.1 Hz, 1H), 4.88–4.85 (m, 2H), 4.76 (t, *J* = 6.2 Hz, 2H), 4.21 (q, *J* = 7.0 Hz, 2H), 1.96–1.90 (m, 2H), 1.56 (dq, *J* = 14.7, 7.4 Hz, 2H), 1.40 (t, *J* = 7.0 Hz, 3H), 0.99 (t, *J* = 7.4 Hz, 3H); ^13^C NMR (126 MHz, Acetone-d_6_) δ 159.9, 151.8, 150.7, 142.5, 121.5 (q, *J* = 323.6 Hz, *C*F_3_), 118.7, 112.6, 87.0, 73.4, 66.8, 30.2, 18.6, 13.3, 12.9; LRMS-ES+ *m*/*z* (relative intensity) 222.1 (C_13_H_20_NO_2_ M+, 100); HRMS-ES+ (C_13_H_20_NO_2_) calcd 222.1494 (M+), found 222.1497.

##### 4.4.40.2 Spectral Data for Product **50**

^1^H NMR (500 MHz, Acetone-d_6_) δ 8.58 (ddd, *J* = 9.1, 7.4, 1.9 Hz, 1H), 8.54–8.48 (m, 1H), 7.72 (dd, *J* = 8.9, 0.8 Hz, 1H), 7.61 (ddd, *J* = 7.2, 6.1, 1.0 Hz, 1H), 4.60 (t, *J* = 6.5 Hz, 2H), 1.88 (ddt, *J* = 9.0, 7.8, 6.4 Hz, 2H), 1.57–1.49 (m, 2H), 0.97 (t, *J* = 7.4 Hz, 3H); ^13^C NMR (126 MHz, Acetone-d_6_) δ 160.8, 148.6, 139.5, 120.1 (q, *J* = 320.9 Hz, *C*F_3_), 118.5, 111.9, 71.4, 30.3, 18.5, 13.0; LRMS-ES+ *m*/*z* (relative intensity) 152.1 (C_9_H_14_NO M+, 25); HRMS-ES+ (C_9_H_14_NO) calcd 152.1075 (M+), found 152.1068.

## Supplementary Material



## Notes

Images of ^1^H and ^13^C­{^1^H} NMR spectra of synthesized products (PDF)
