# Supplementary material for: Room-Temperature Nucleophilic Aromatic Substitution of 2‑Halopyridinium Ketene Hemiaminals with Sulfur Nucleophiles
Source: ACS Omega. 2025 Nov 17;10(47):57335–47. doi: 10.1021/acsomega.5c07504 (PMC12676317; doi:10.1021/acsomega.5c07504)

Supporting Information

Room-Temperature Nucleophilic Aromatic Substitution of 2-Halopyridinium Ketene Hemiaminals with Sulfur Nucleophiles

Jordan C. Merklin, Beau A. Sinardo, Claire Y. Cooper, Sudchananya Udomphan, Madeleine H. Boger, and Max M. Majireck

Chemistry Department, Hamilton College, 198 College Hill Road, Clinton, NY, 13323, USA

*Correspondence: mmajirec@hamilton.edu; Tel.: +001-315-859-4742

Contents:

Title page……………………………………………………………………………….........................……….S1
^{1}H and $^{13}C\{^{1}H\}$ NMR spectra of synthesized products in numerical order…….. S2- S42

Figure S1. ^{1}H NMR Spectrum of **5** (500 MHz, $CDCl_3$)

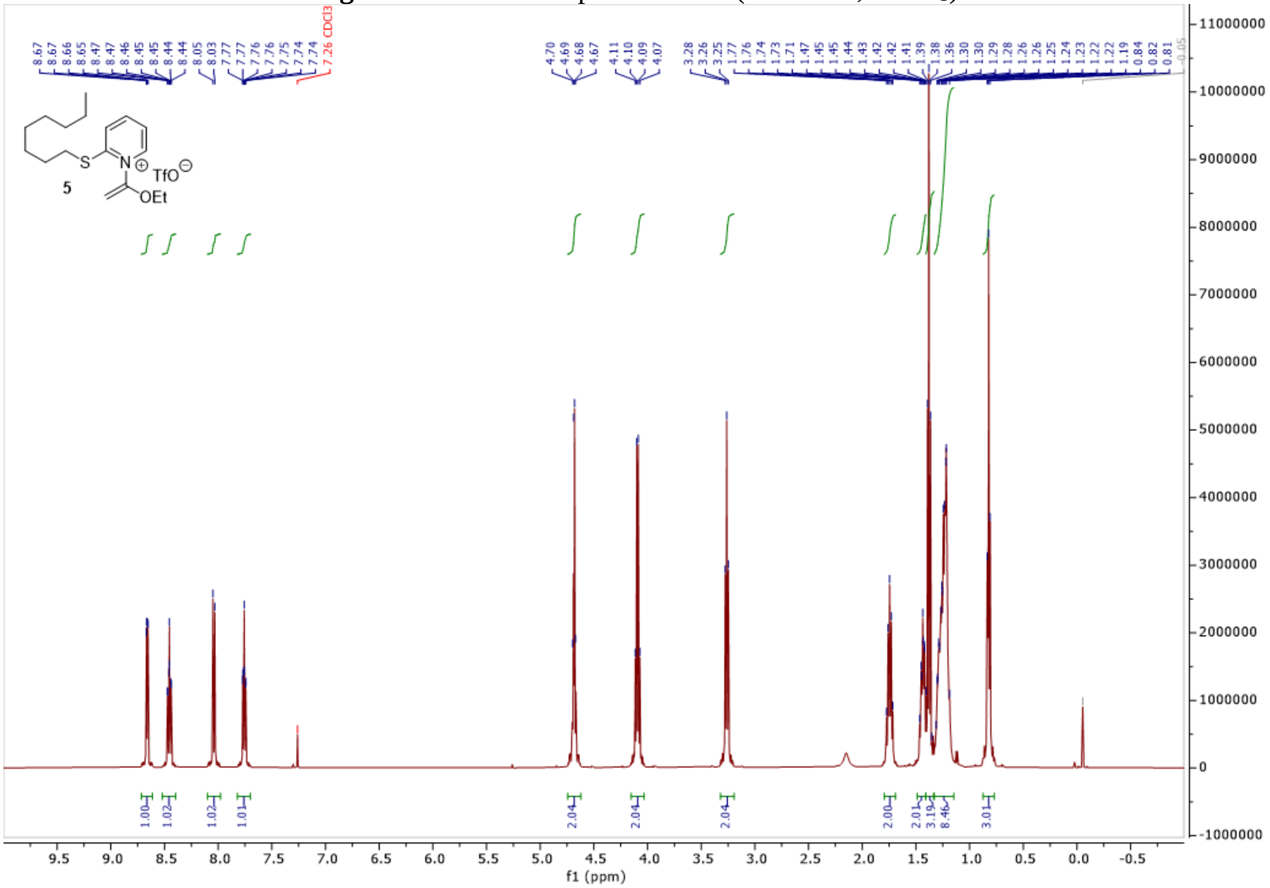

Figure S2. $^{13}C\{^{1}H\}$ NMR Spectrum of **5** (126 MHz, $CDCl_3$)

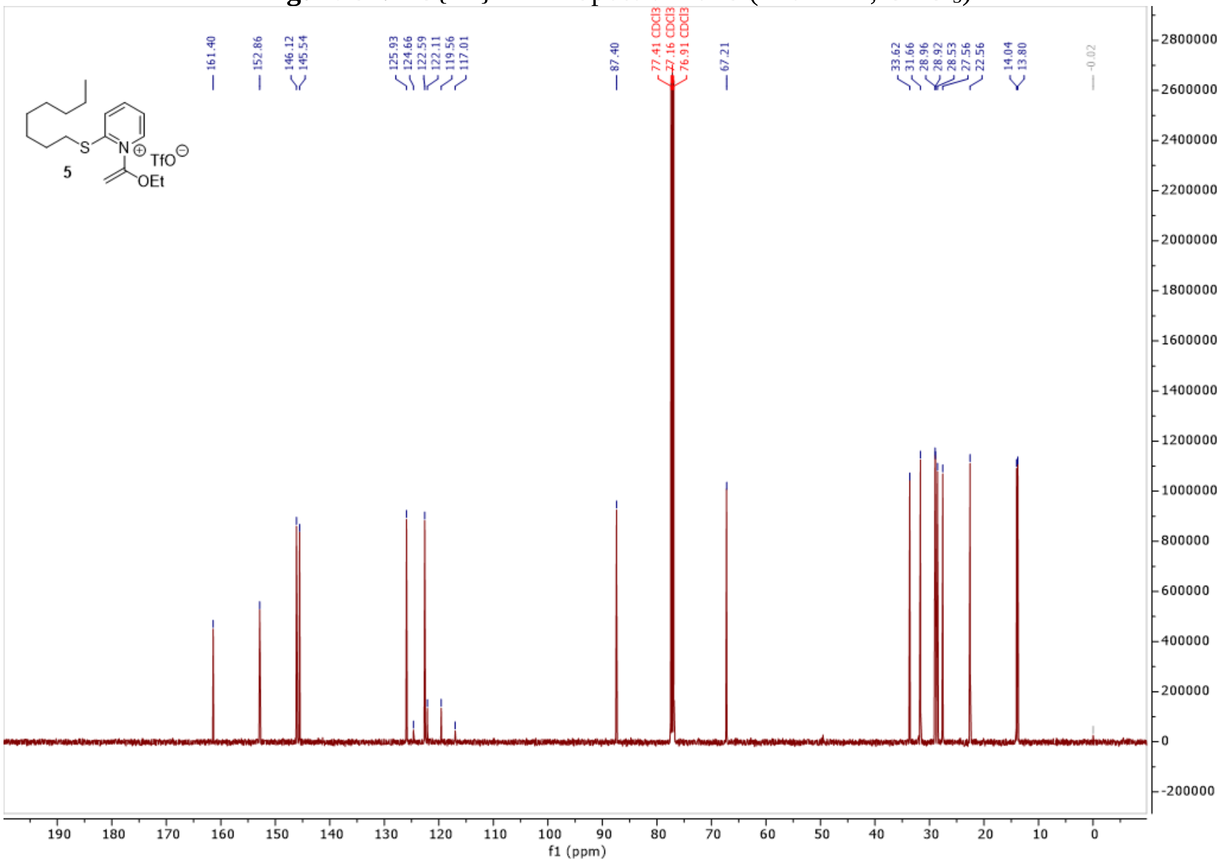

Figure S3. ^{1}H NMR Spectrum of **6** (500 MHz, Acetone-d_6)

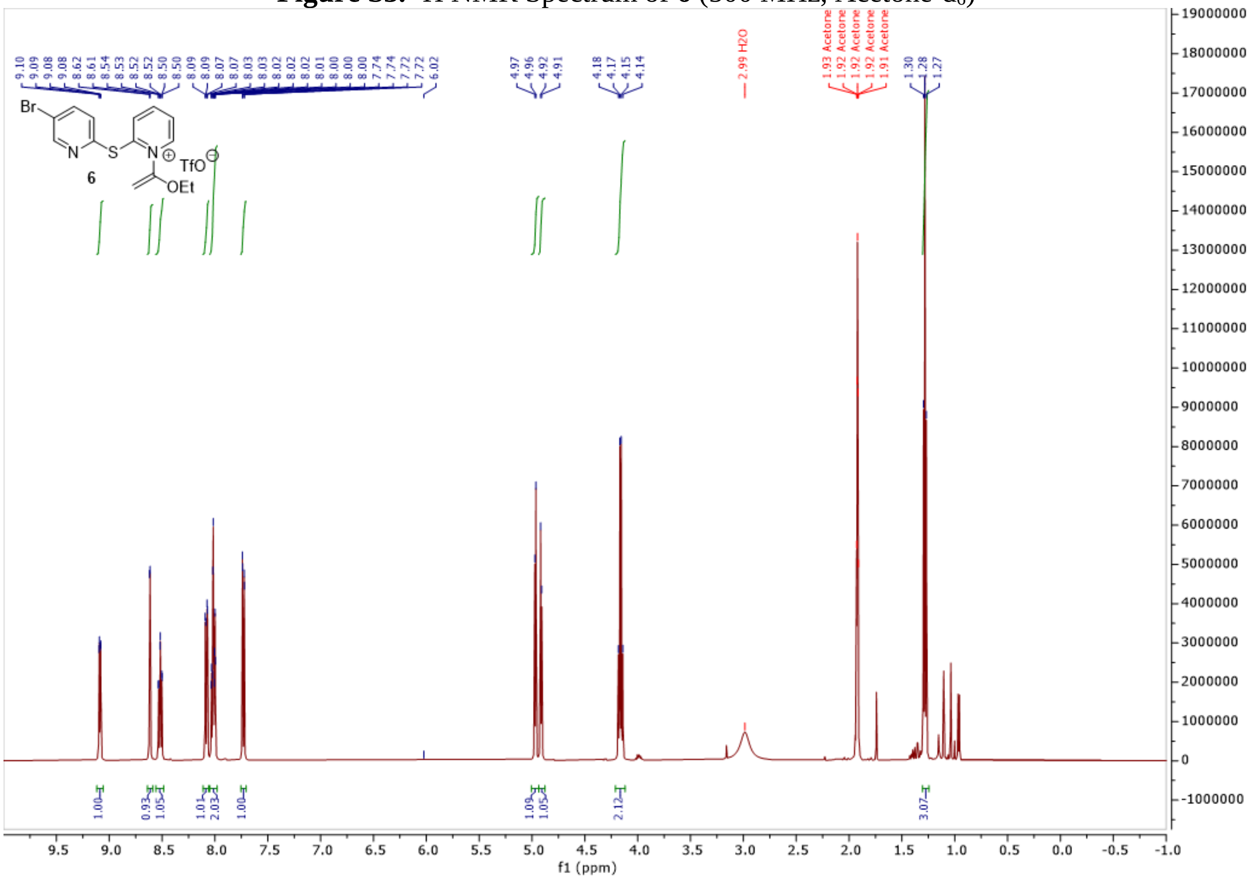

Figure S4. ^{13}C{^{1}H} NMR Spectrum of **6** (126 MHz, Acetone-d_6)

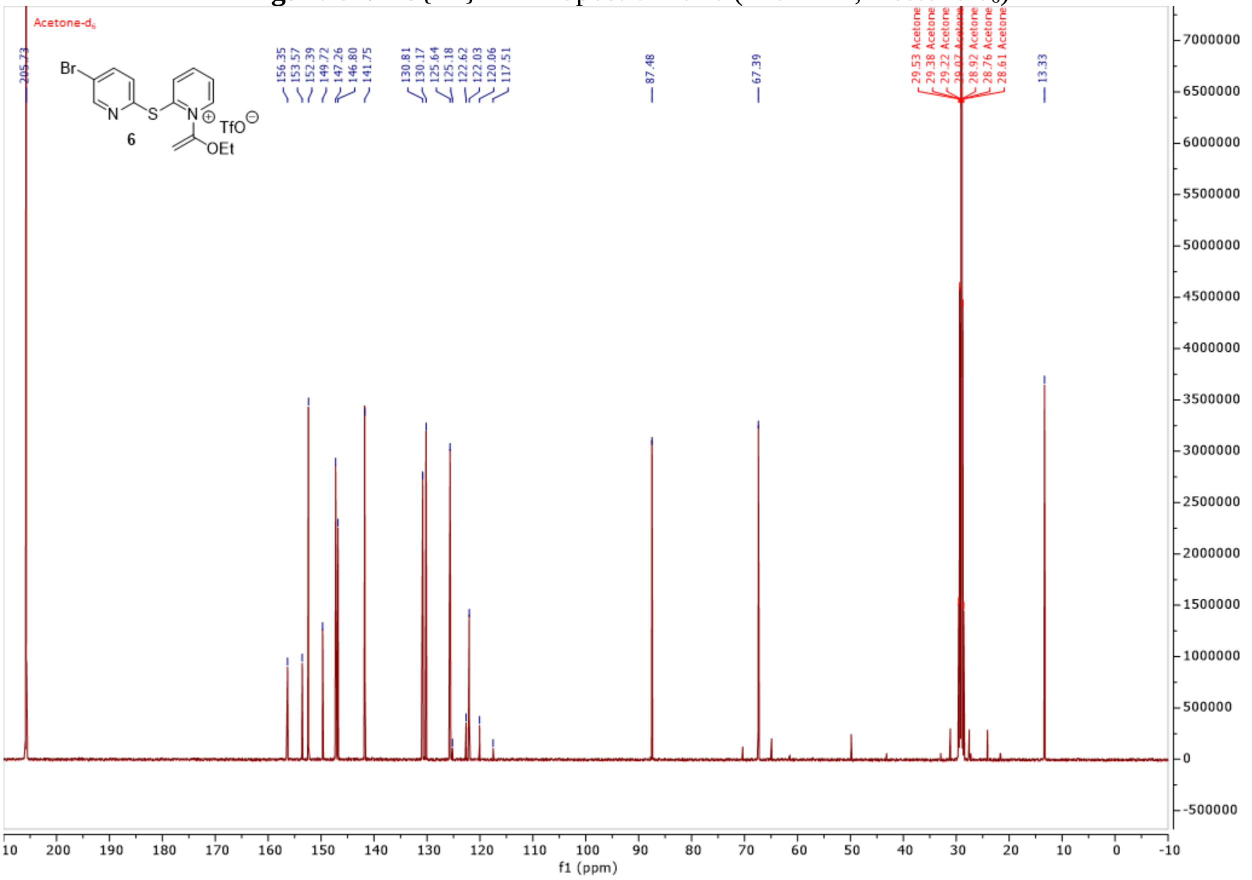

Figure S5. ^{1}H NMR Spectrum of **7** (500 MHz, Acetone-d_6)

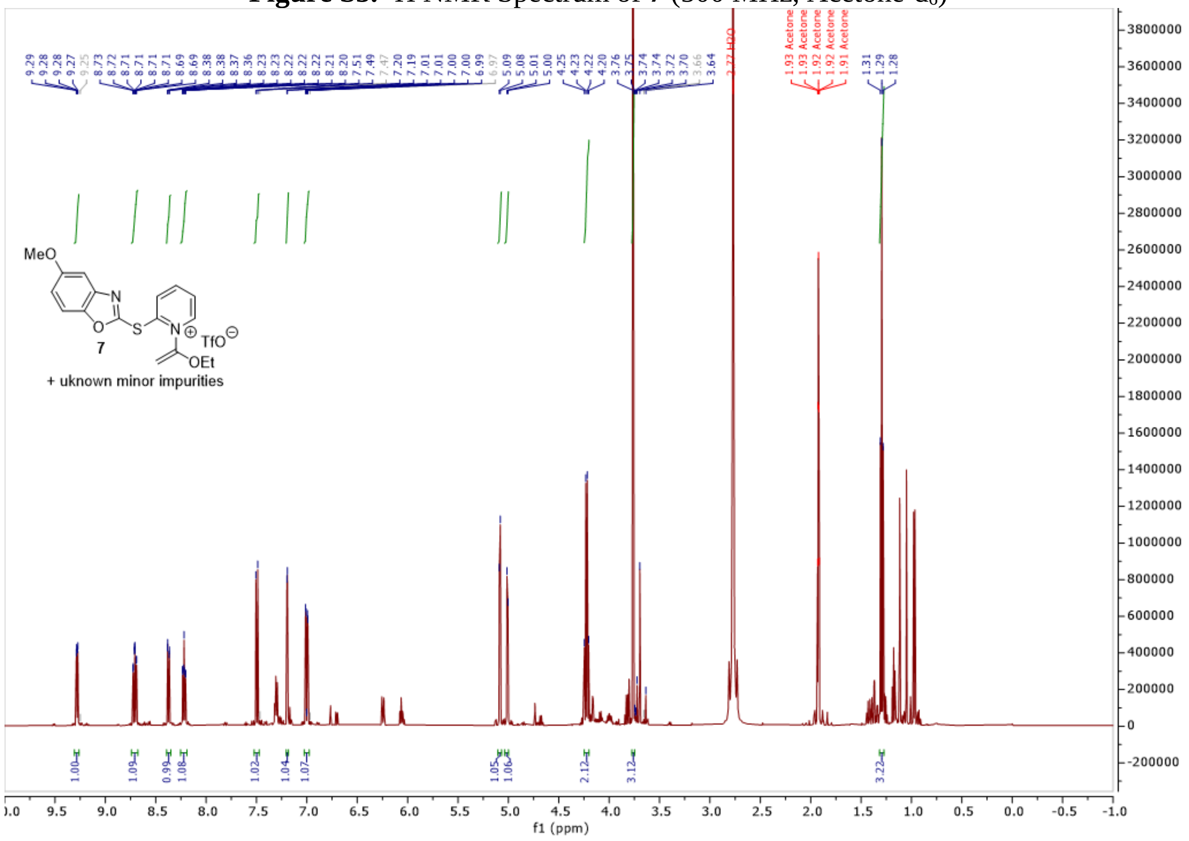

Figure S6. ^{13}C{^{1}H} NMR Spectrum of **7** (126 MHz, Acetone-d_6)

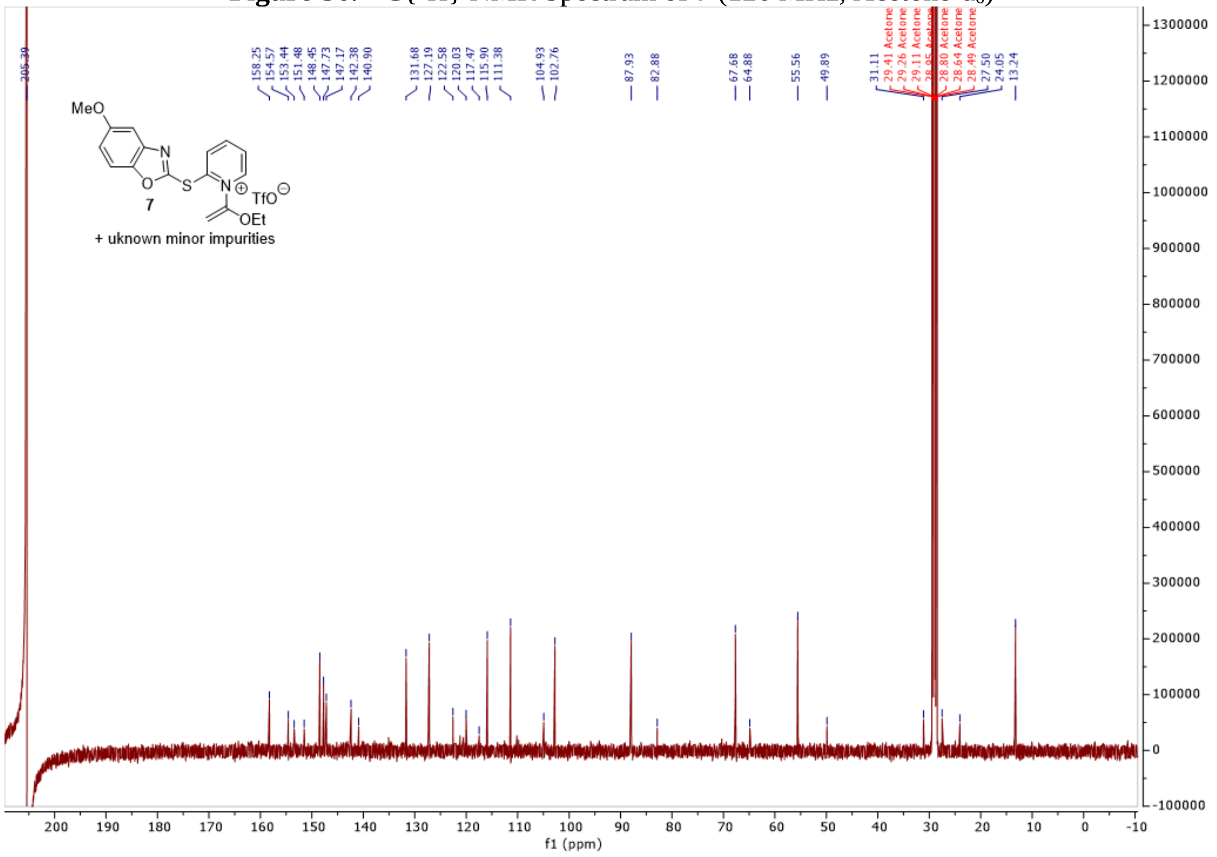

Figure S7. ^{1}H NMR Spectrum of **8** (500 MHz, Acetone-d_6)

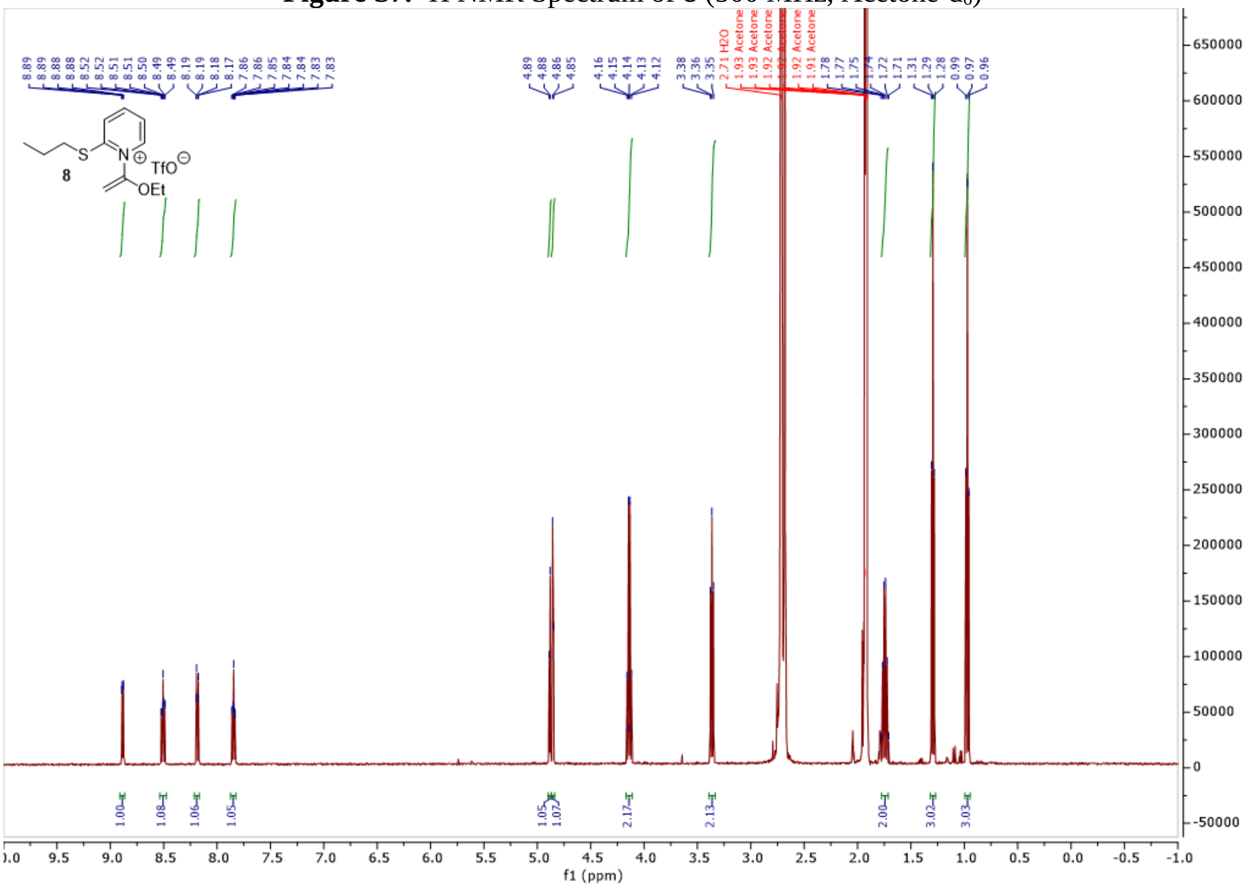

Figure S8. $^{13}C\{^{1}H\}$ NMR Spectrum of **8** (126 MHz, Acetone-d_6)

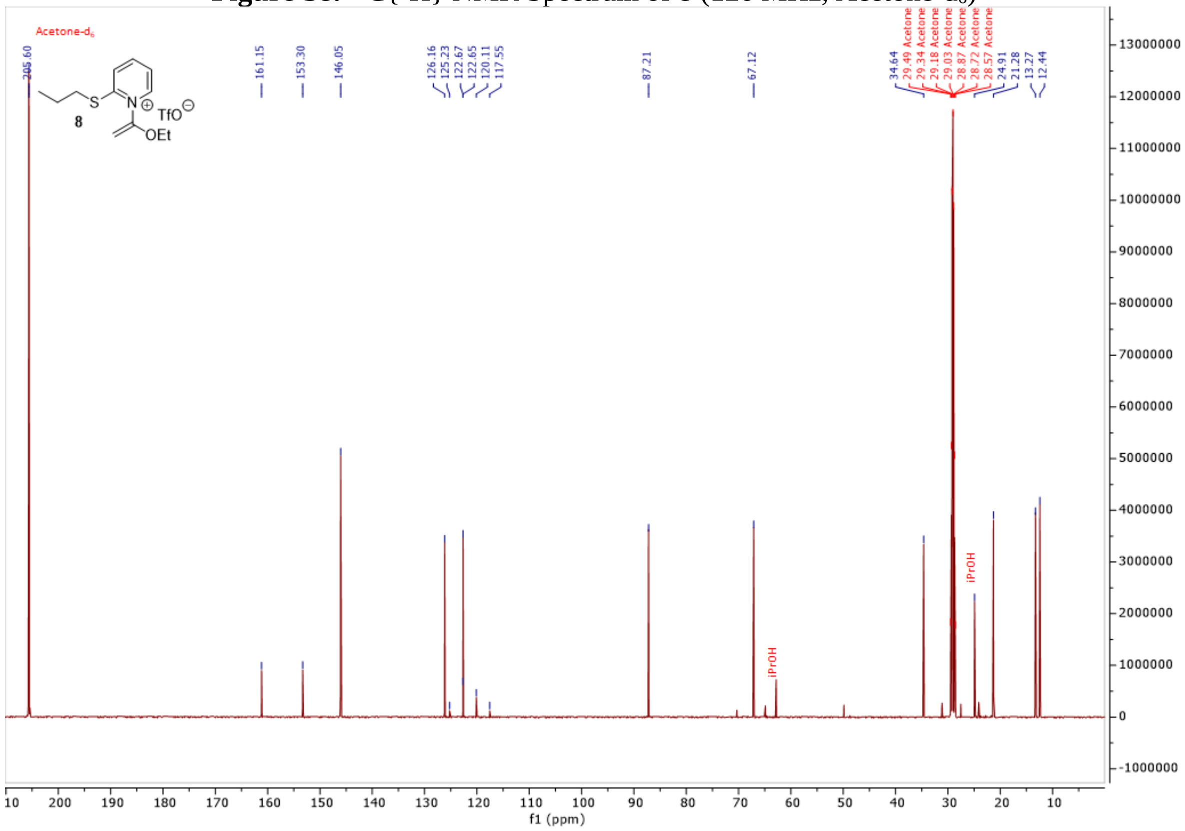

Figure S9. ^{1}H NMR Spectrum of **9** (500 MHz, Acetone-d_6)

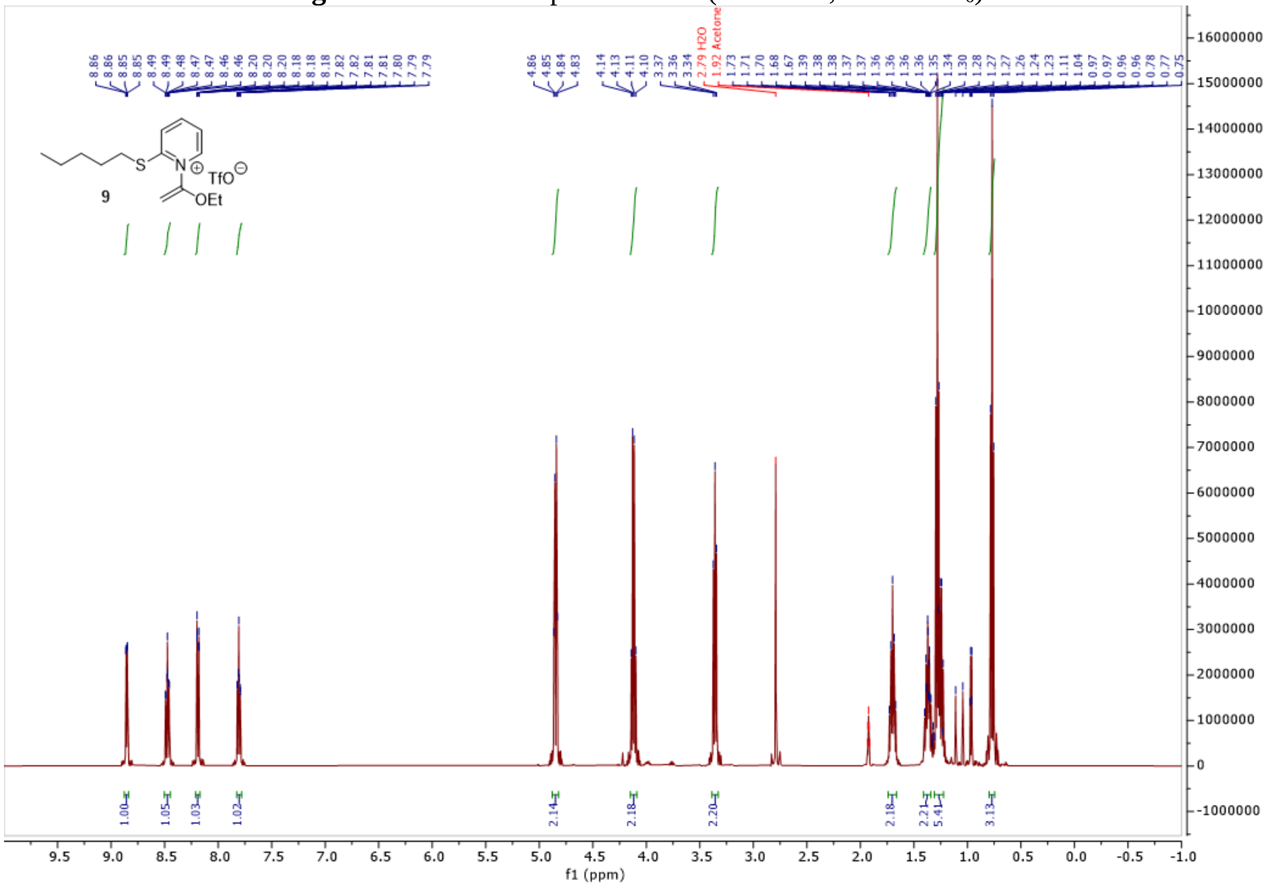

Figure S10. $^{13}C\{^{1}H\}$ NMR Spectrum of **9** (126 MHz, Acetone-d_6)

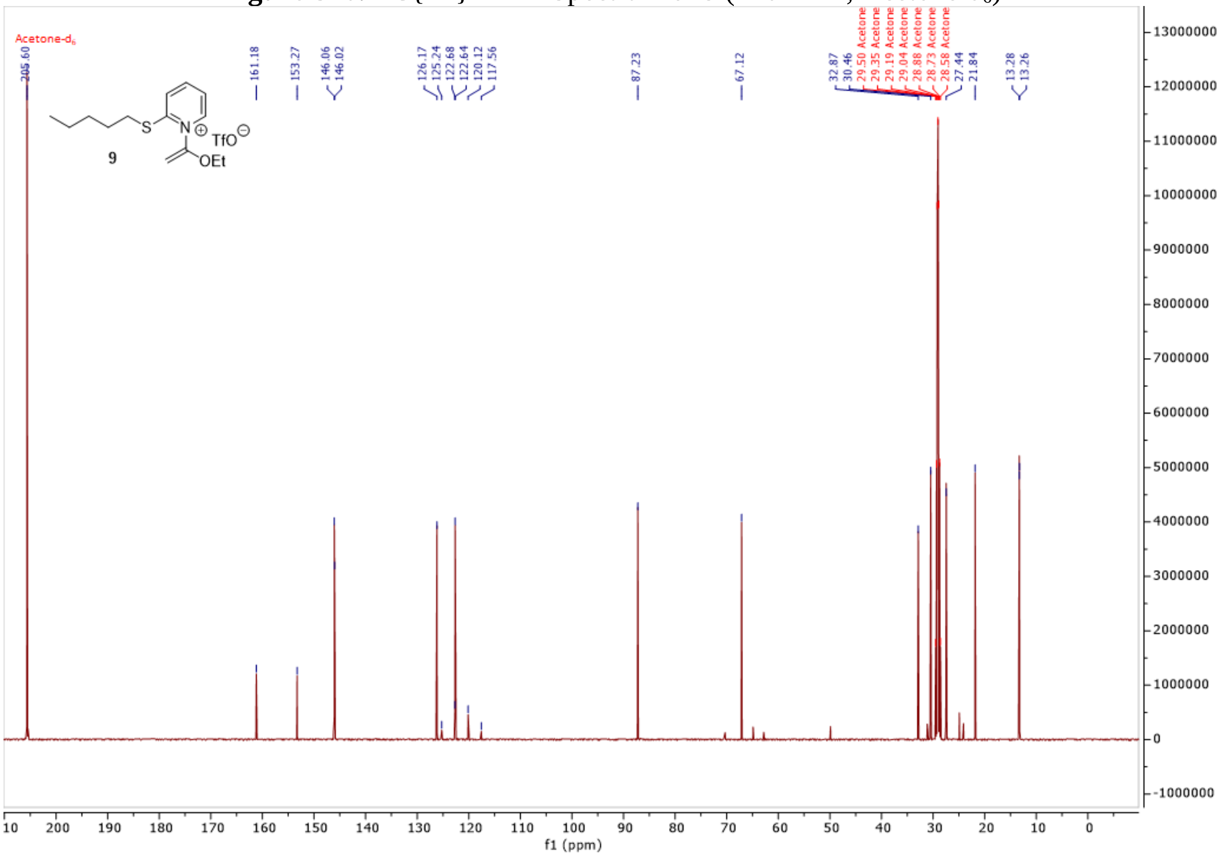

Figure S11. ^{1}H NMR Spectrum of **10** (500 MHz, $CDCl_3$)

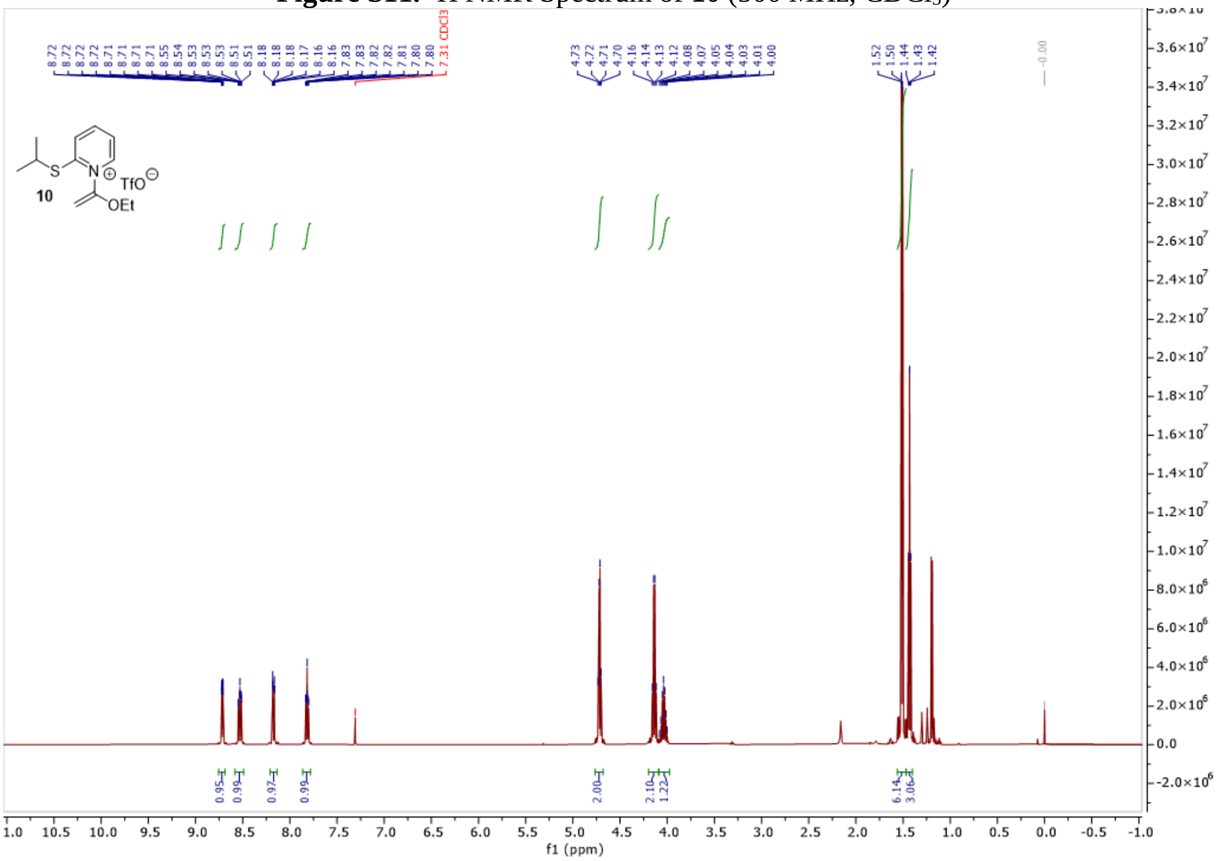

Figure S12. $^{13}C\{^1H\}$ NMR Spectrum of **10** (126 MHz, $CDCl_3$)

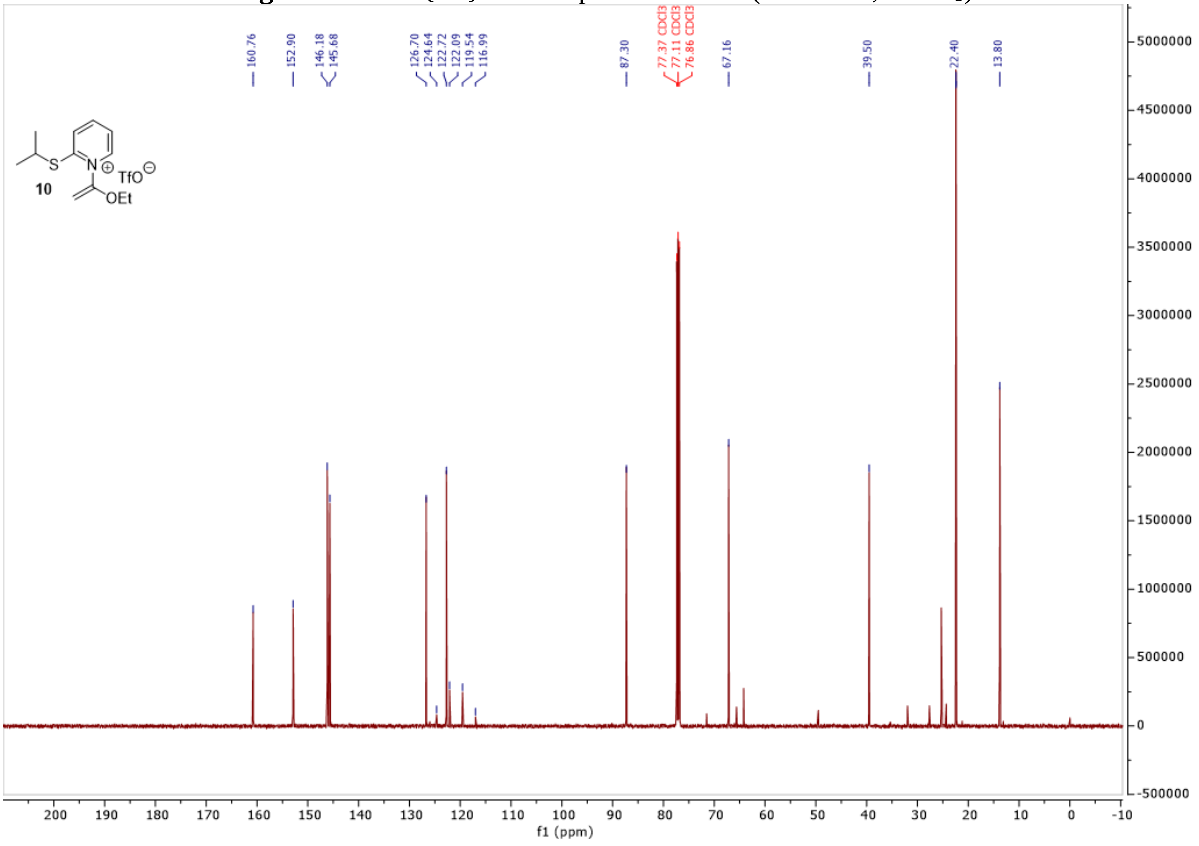

Figure S13. ^{1}H NMR Spectrum of **11** (500 MHz, Acetone-d_6)

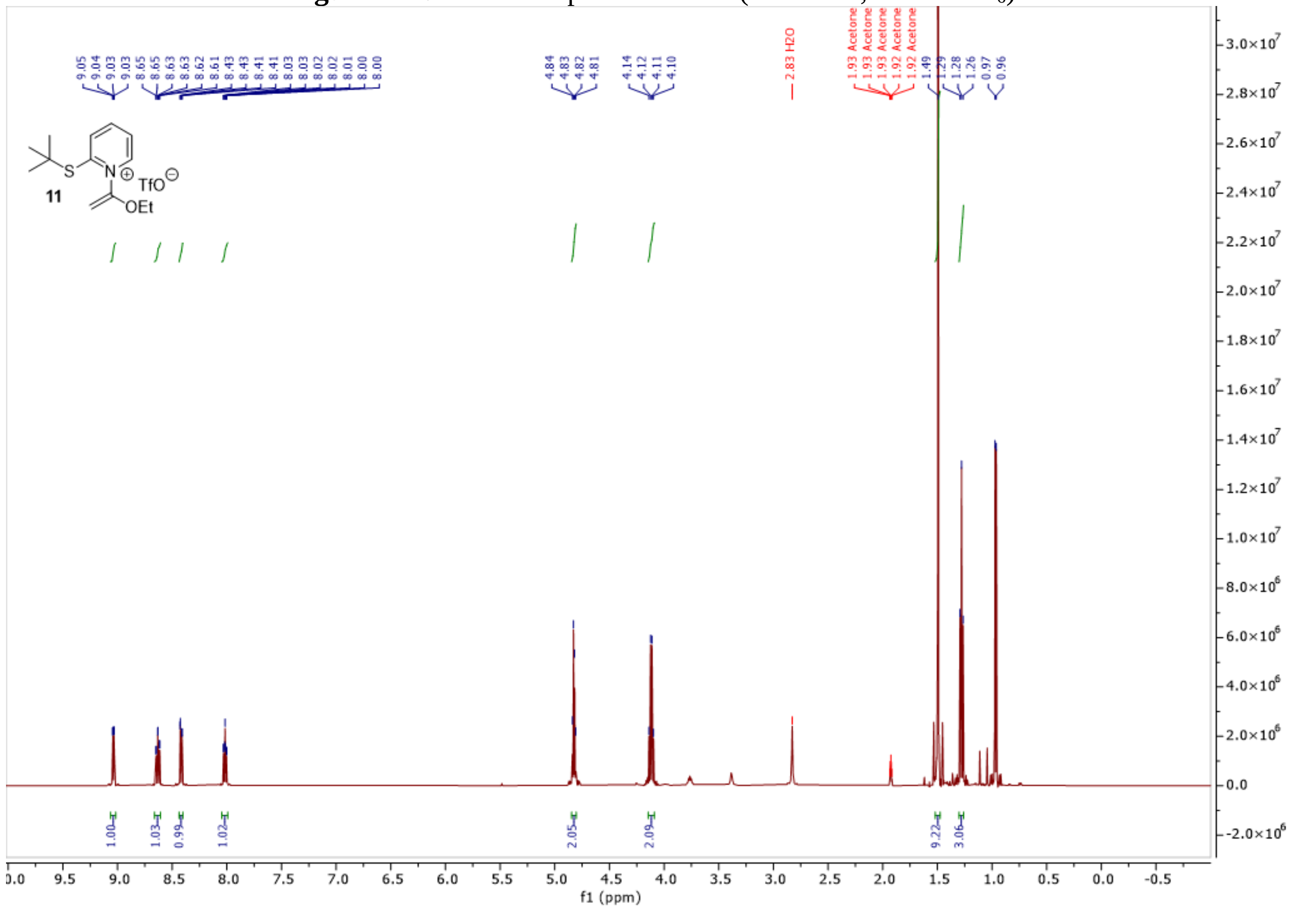

Figure S14. $^{13}C\{^{1}H\}$ NMR Spectrum of **11** (126 MHz, Acetone-d_6)

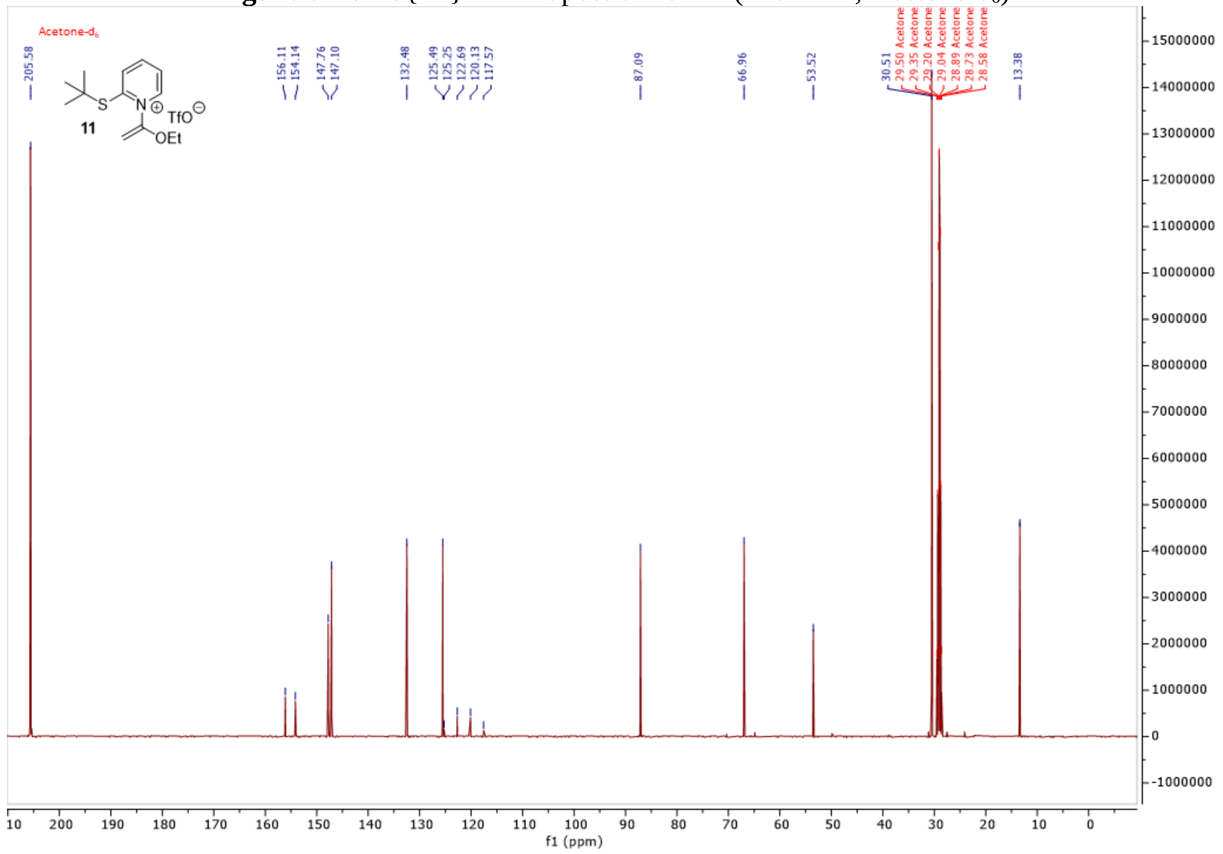

Figure S15. ^{1}H NMR Spectrum of **12** (500 MHz, Acetone-d_6)

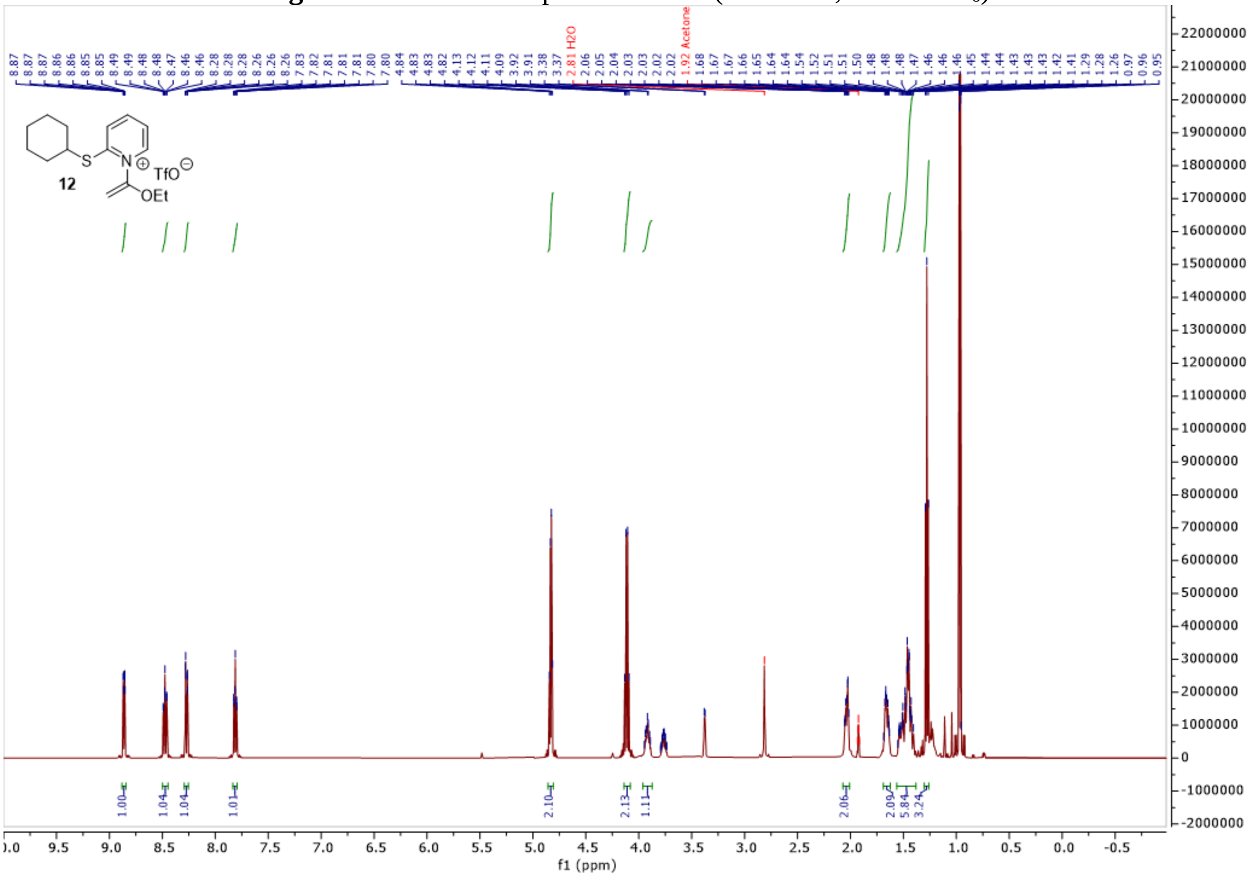

Figure S16. ^{13}C{^{1}H} NMR Spectrum of **12** (126 MHz, Acetone-d_6)

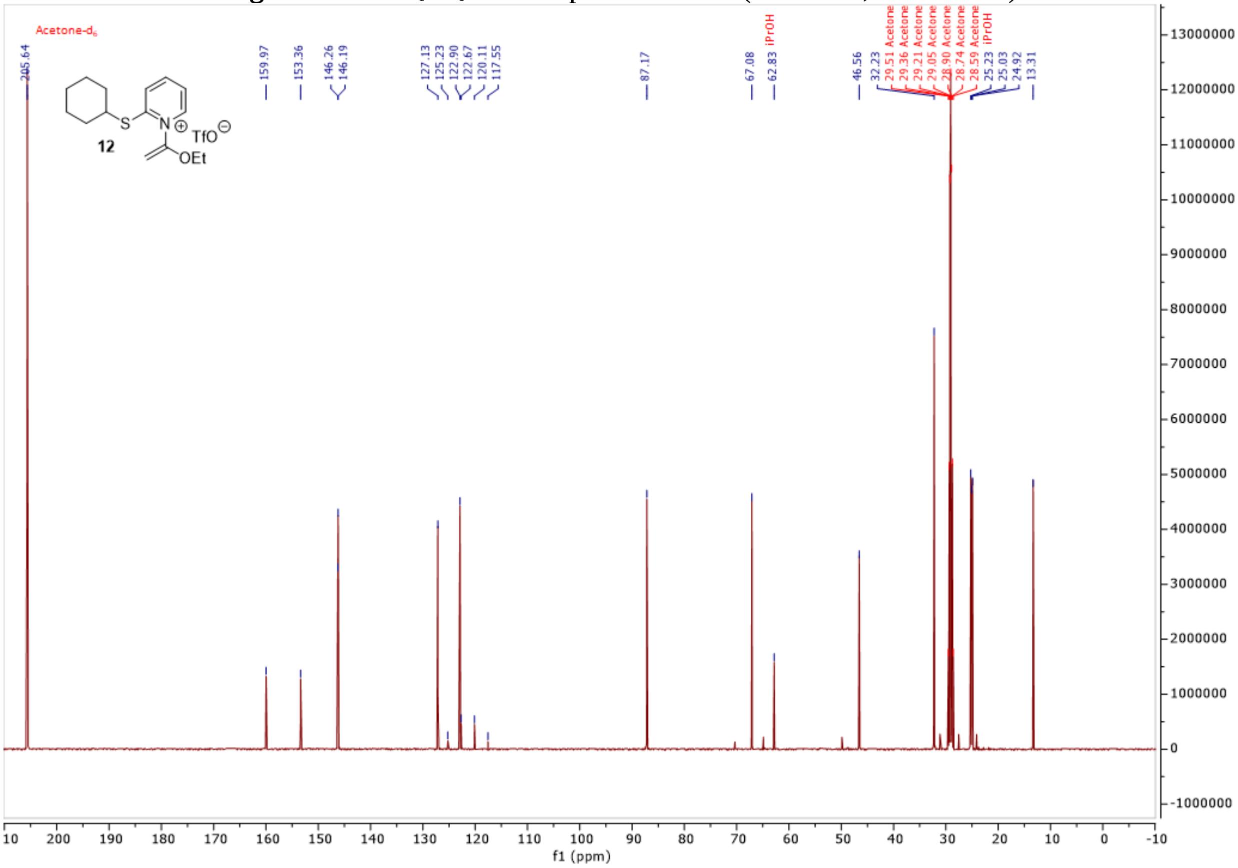

Figure S17. ^{1}H NMR Spectrum of **13** (500 MHz, Acetone-d_6)

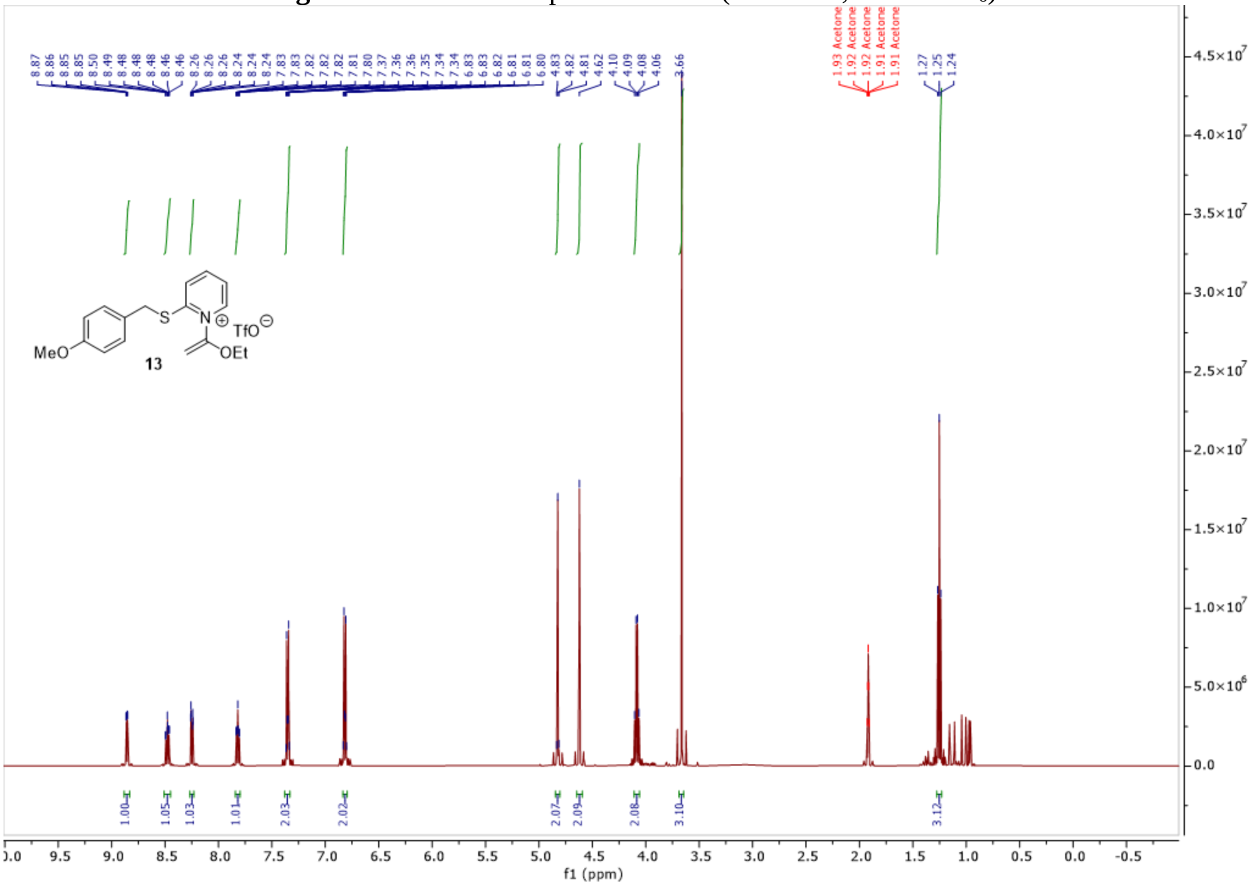

Figure S18. $^{13}C\{^{1}H\}$ NMR Spectrum of **13** (126 MHz, Acetone-d_6)

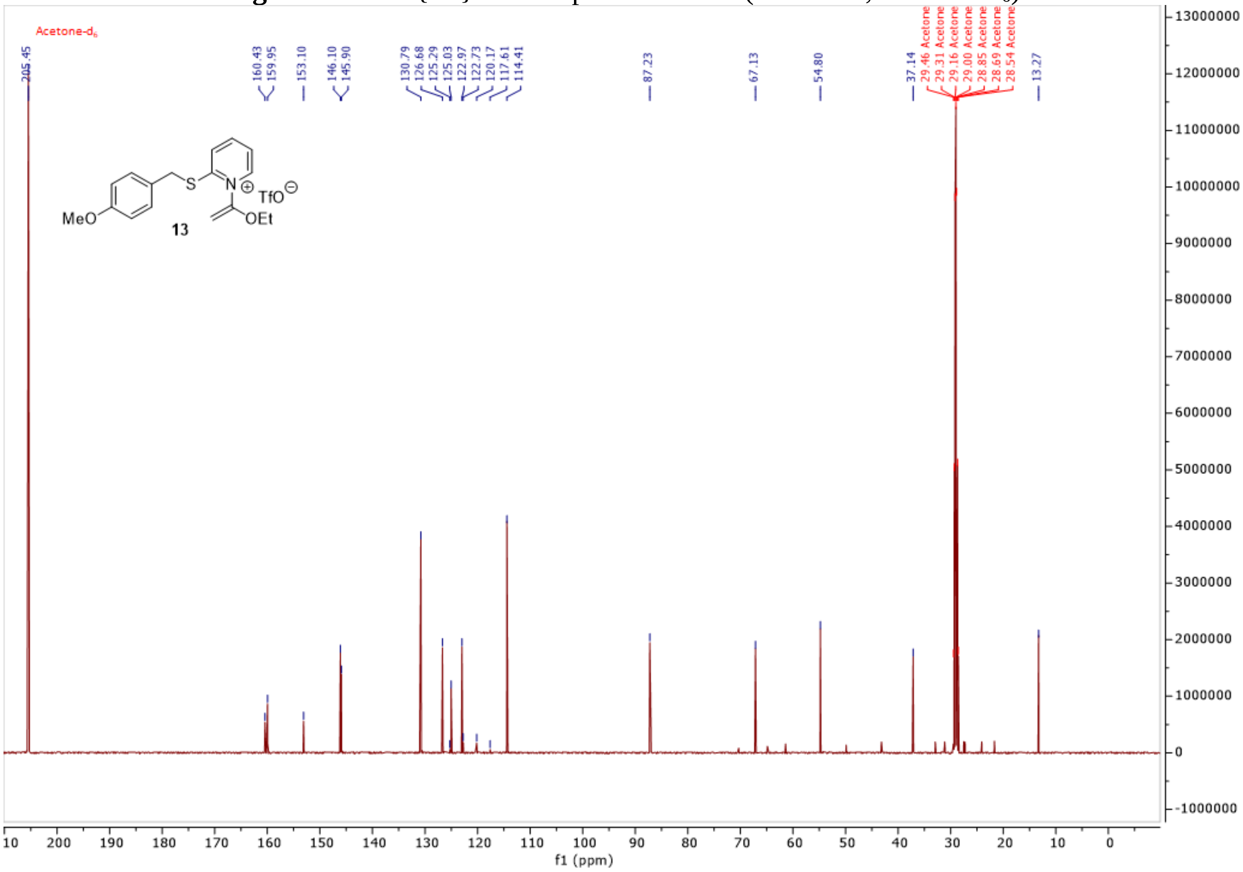

Figure S19. ^{1}H NMR Spectrum of **14** (500 MHz, Acetone-d$_6$)

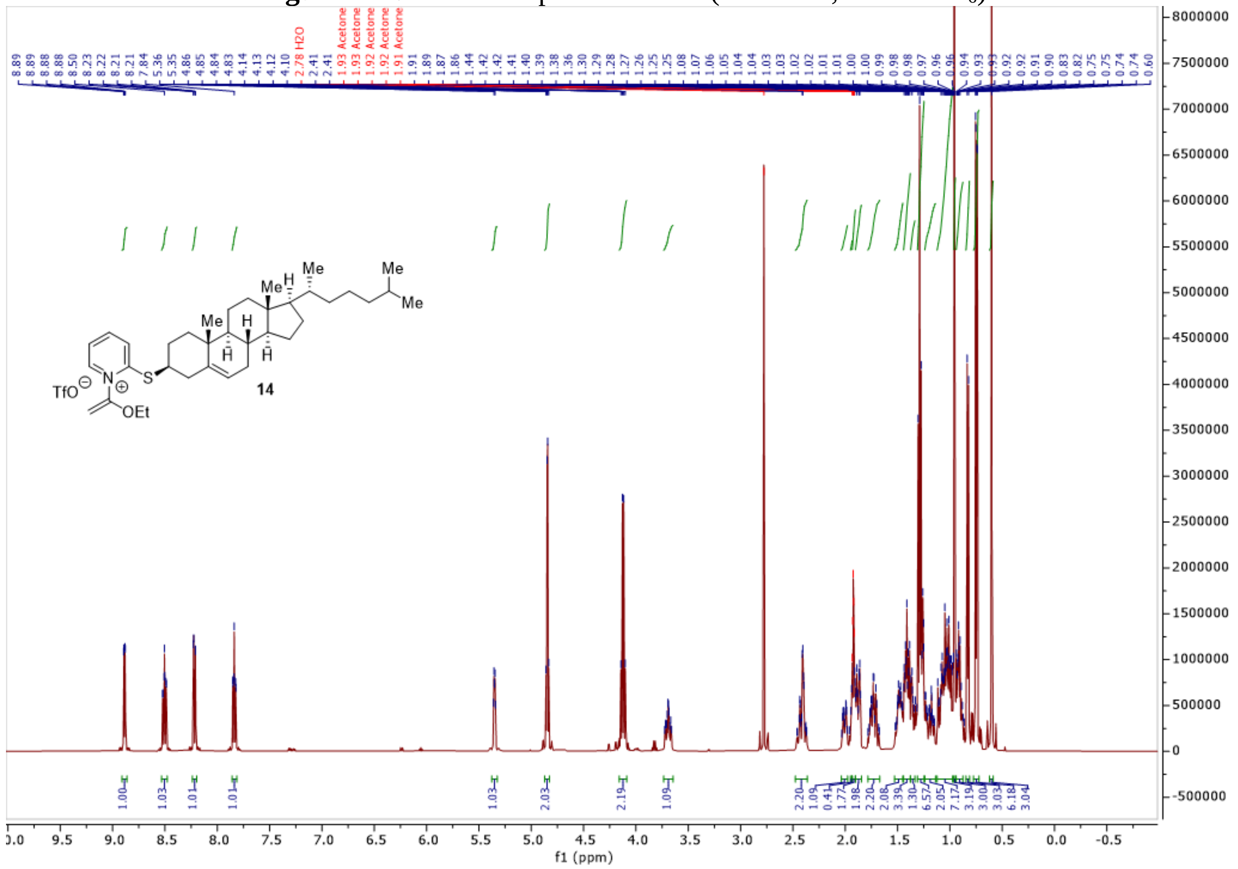

Figure S20. ^{13}C{^{1}H} NMR Spectrum of **14** (126 MHz, Acetone-d$_6$)

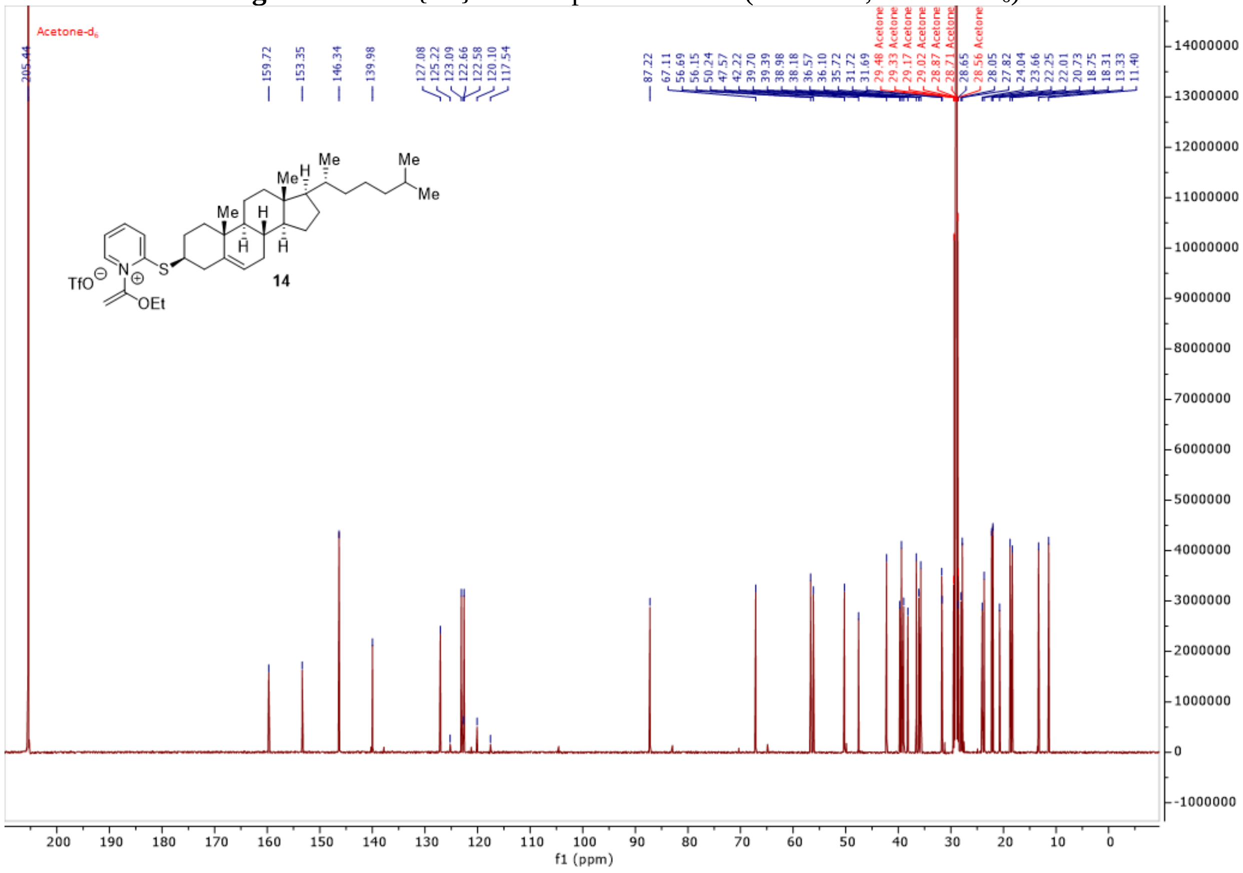

Figure S21. ^{1}H NMR Spectrum of **15** (500 MHz, Acetone-d$_6$)

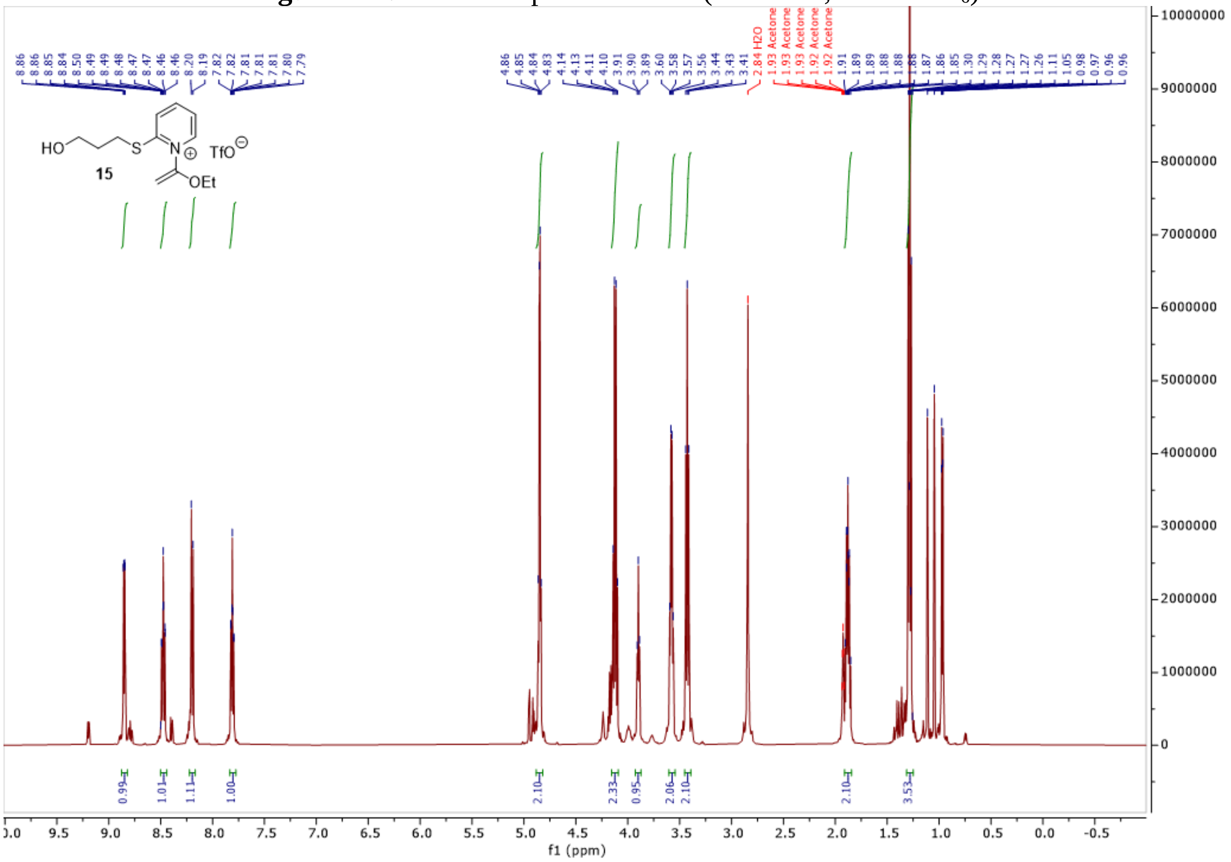

Figure S22. ^{13}C{^{1}H} NMR Spectrum of **15** (126 MHz, Acetone-d$_6$)

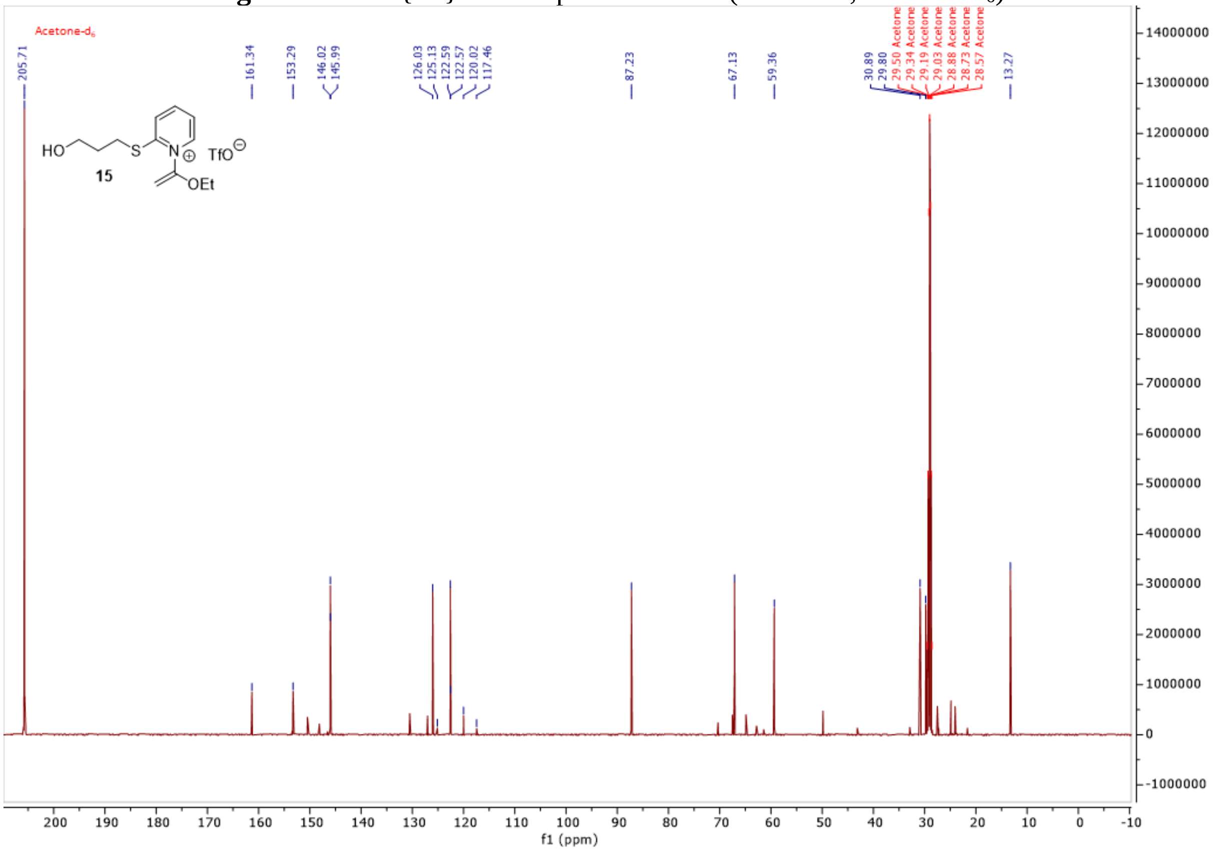

Figure S23. ^{1}H NMR Spectrum of **16** (500 MHz, Acetone-d_6)

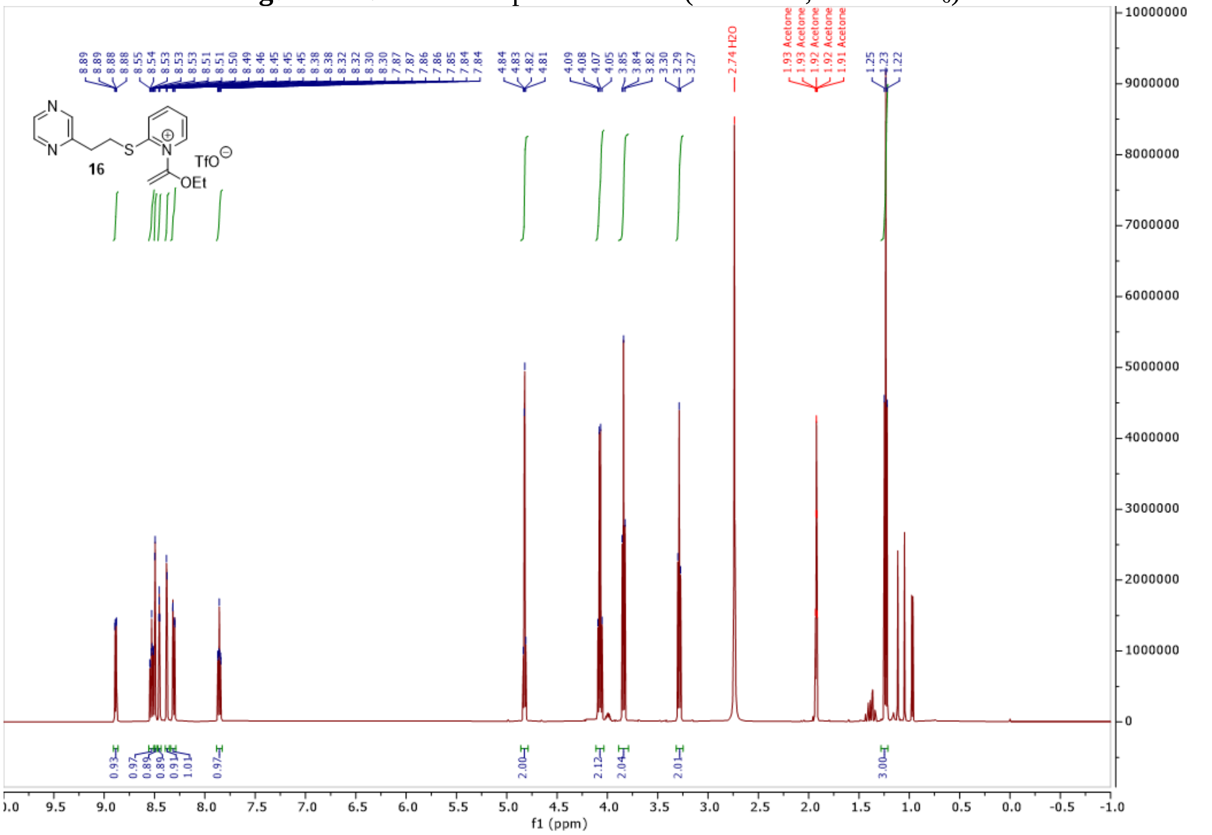

Figure S24. $^{13}C\{^{1}H\}$ NMR Spectrum of **16** (126 MHz, Acetone-d_6)

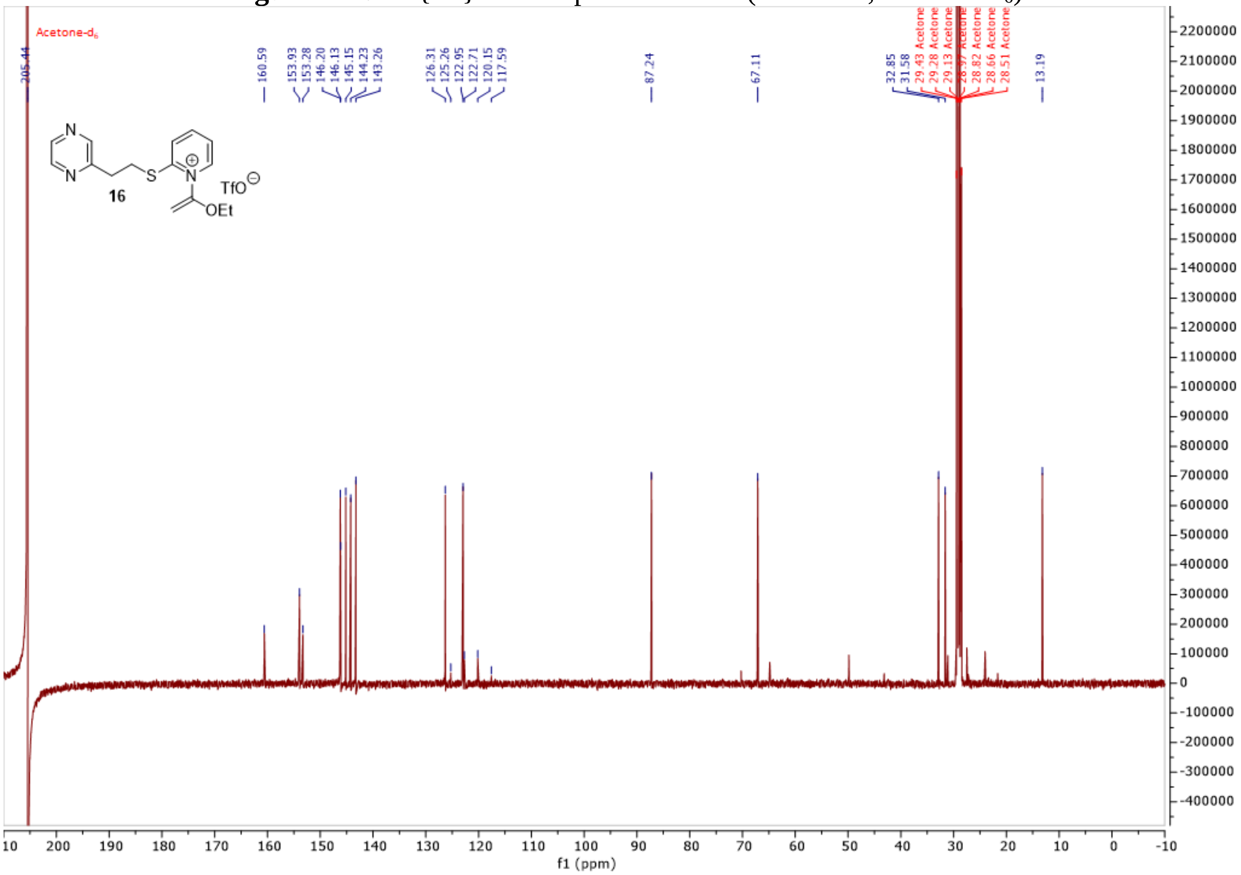

Figure S25. ^{1}H NMR Spectrum of **18** (500 MHz, Acetone-d_6)

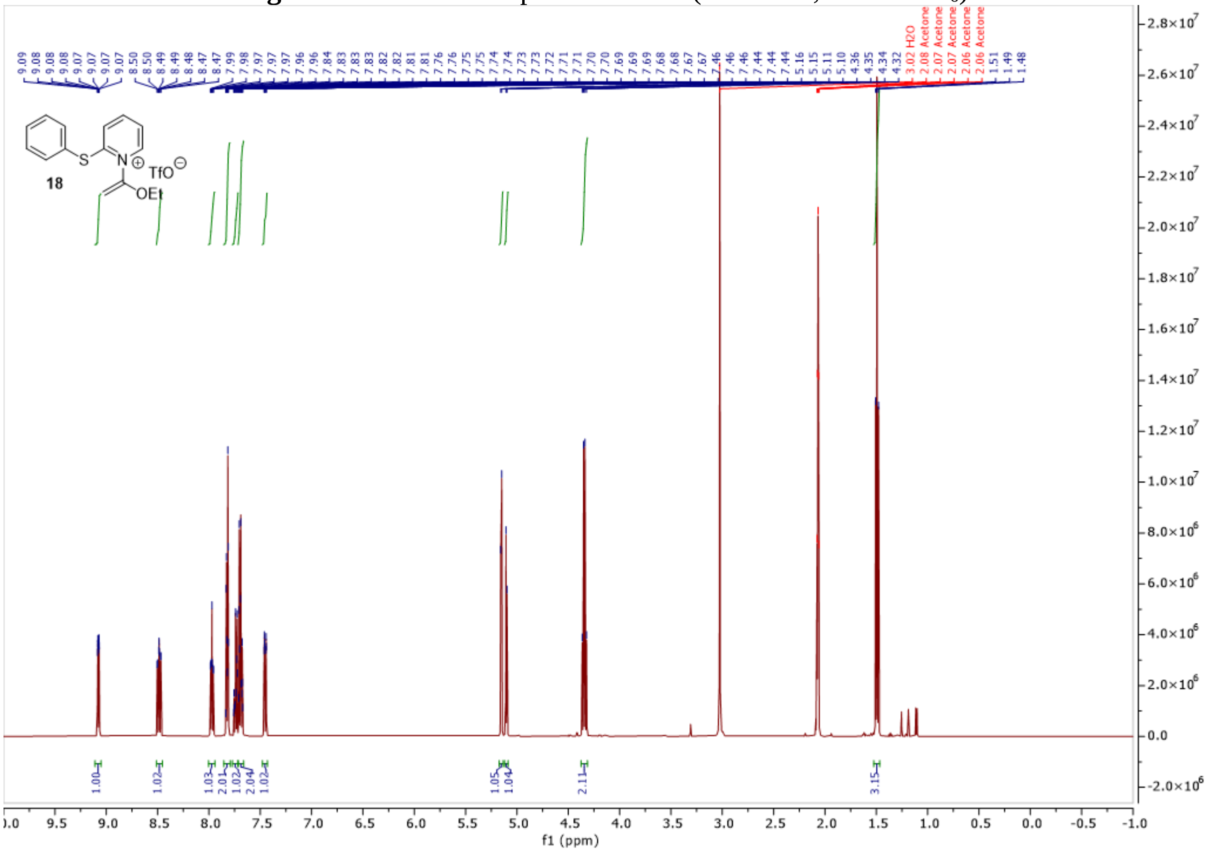

Figure S26. $^{13}C\{^{1}H\}$ NMR Spectrum of **18** (126 MHz, Acetone-d_6)

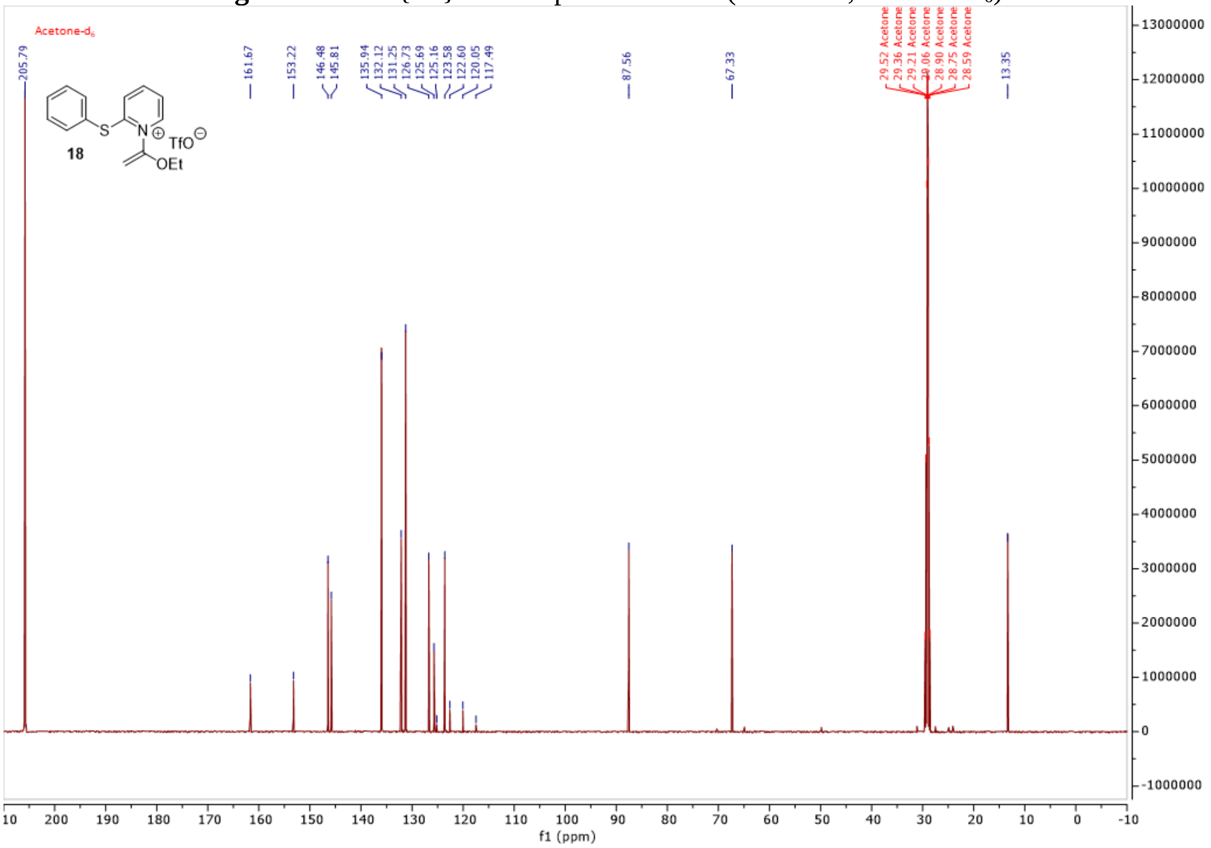

Figure S27. ^{1}H NMR Spectrum of **19** (500 MHz, Acetone-d_6)

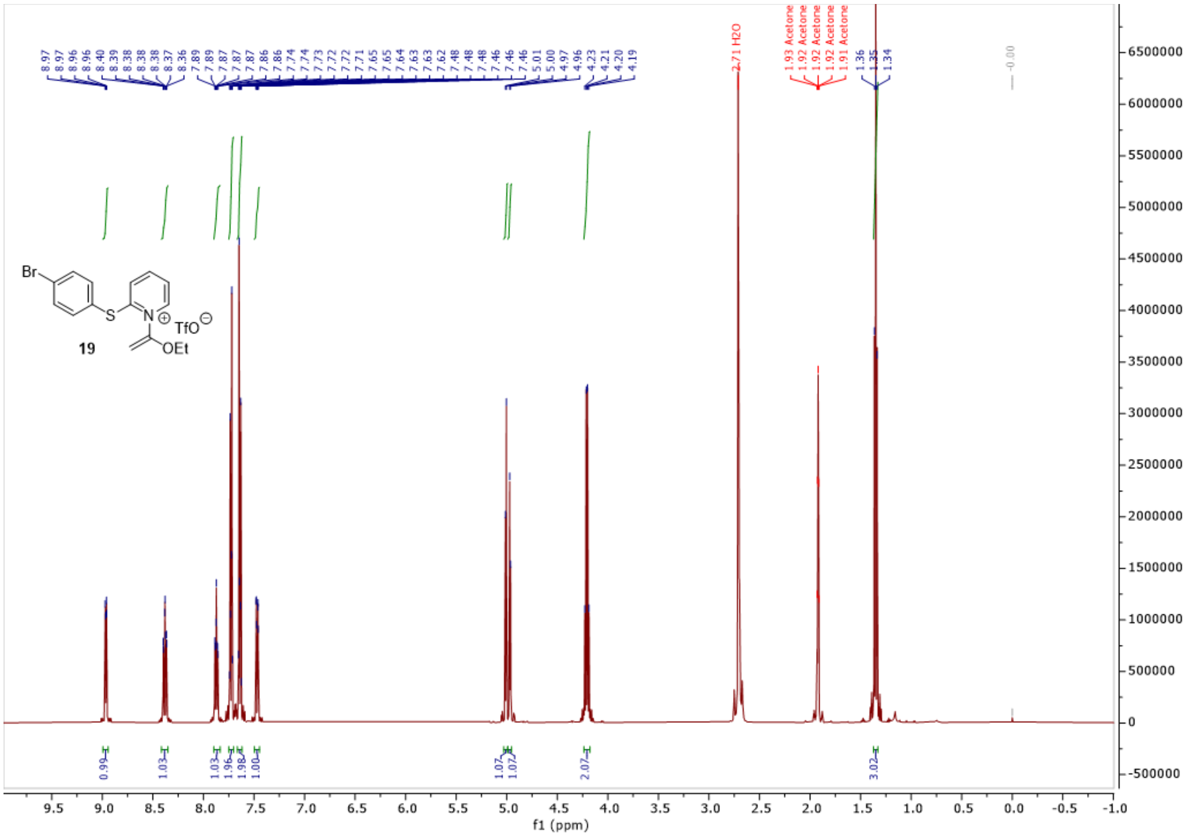

Figure S28. $^{13}C\{^{1}H\}$ NMR Spectrum of **19** (126 MHz, Acetone-d_6)

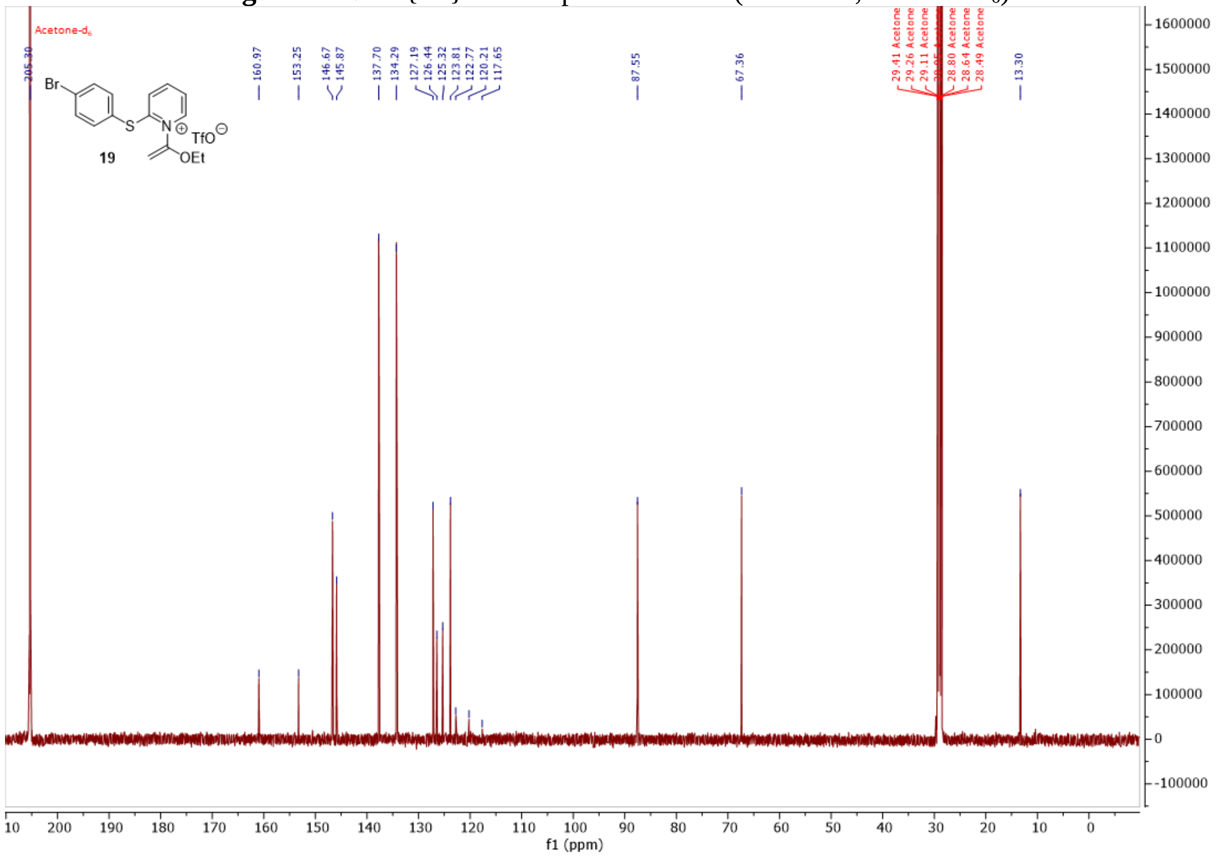

Figure S29. ^{1}H NMR Spectrum of **20** (500 MHz, Acetone-d_6)

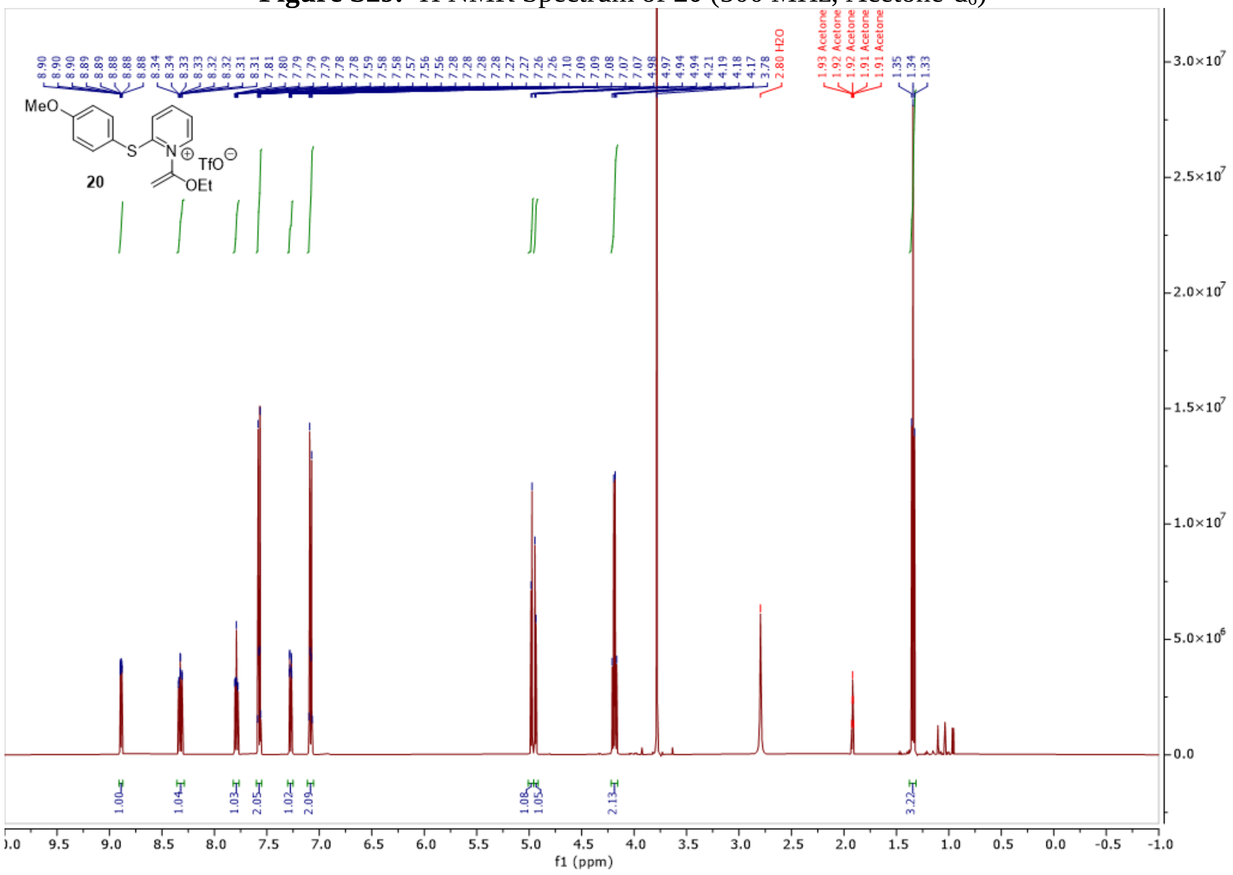

Figure S30. ^{13}C{^{1}H} NMR Spectrum of **20** (126 MHz, Acetone-d_6)

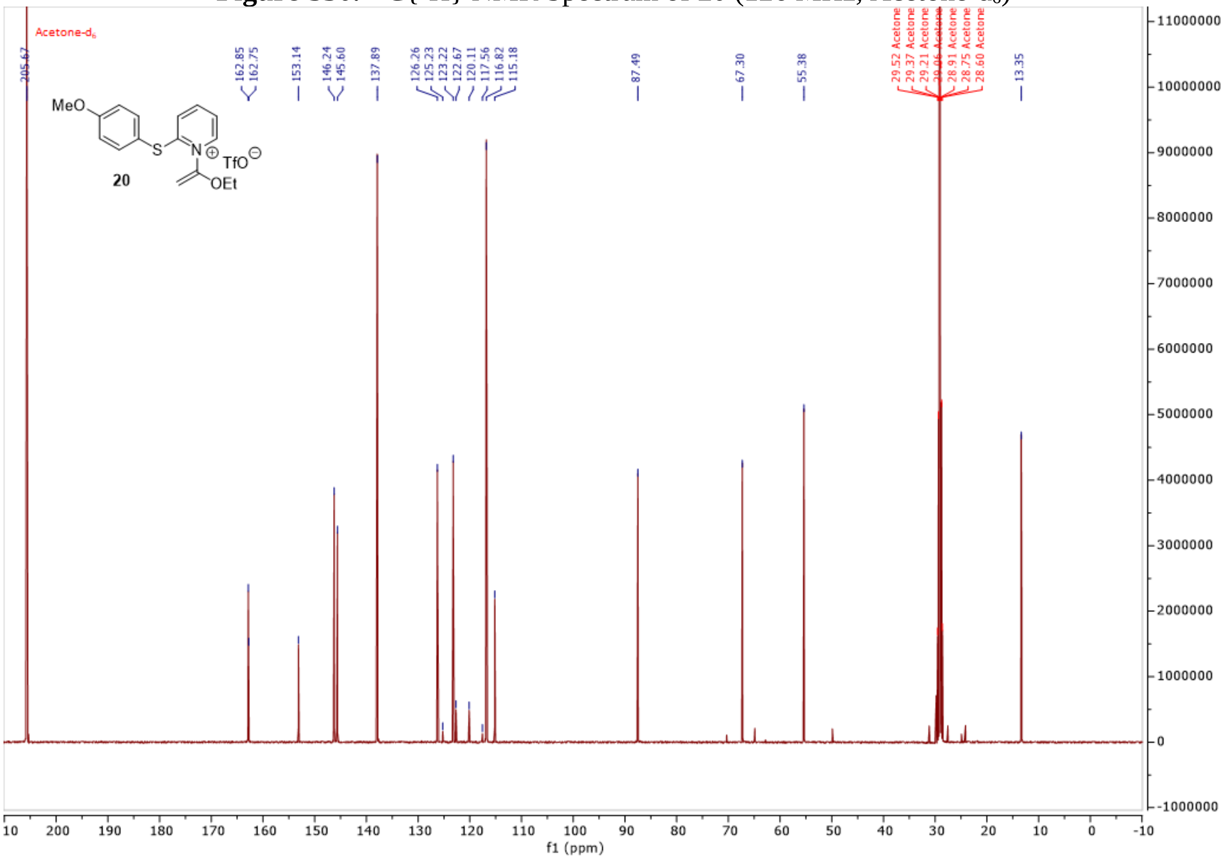

Figure S31. ^{1}H NMR Spectrum of **21** (500 MHz, Acetone-d_6)

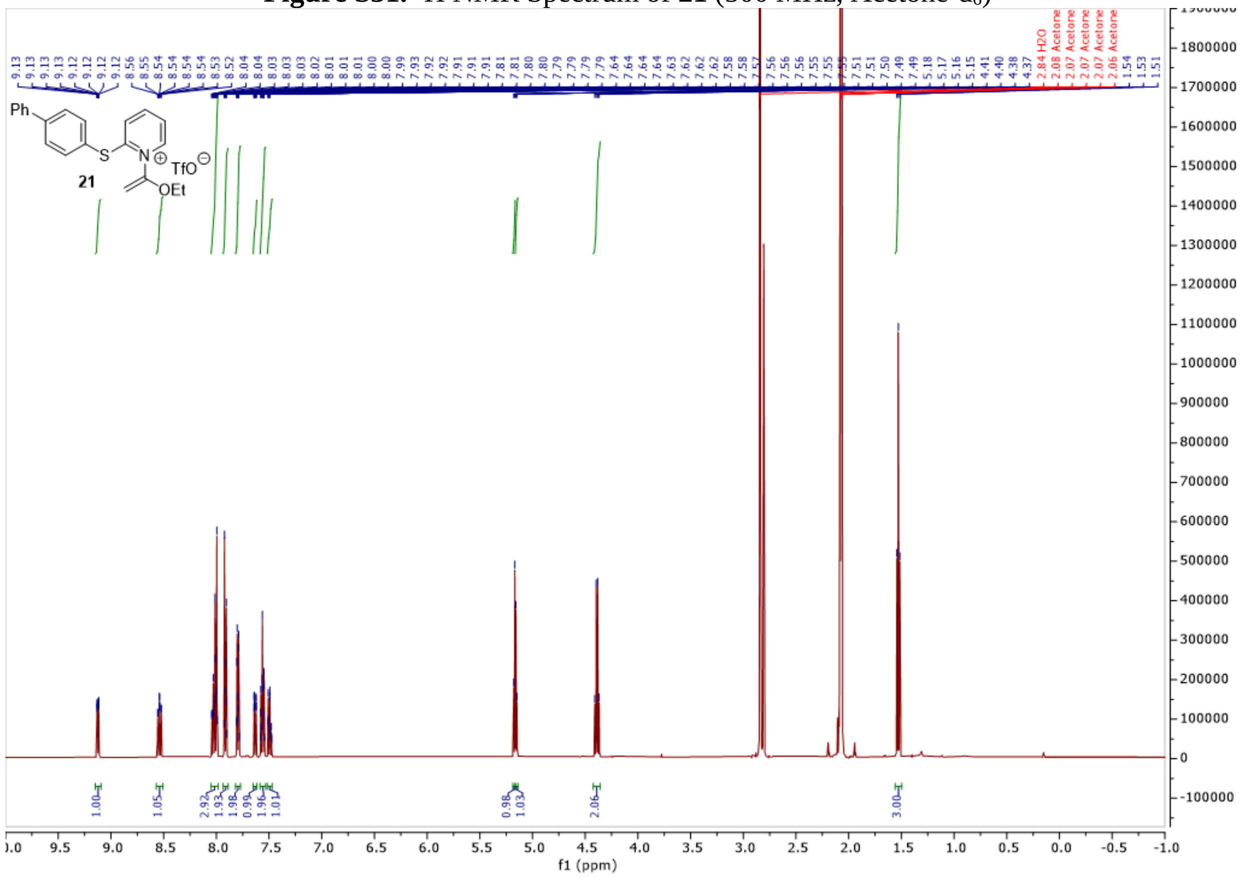

Figure S32. $^{13}C\{^{1}H\}$ NMR Spectrum of **21** (126 MHz, Acetone-d_6)

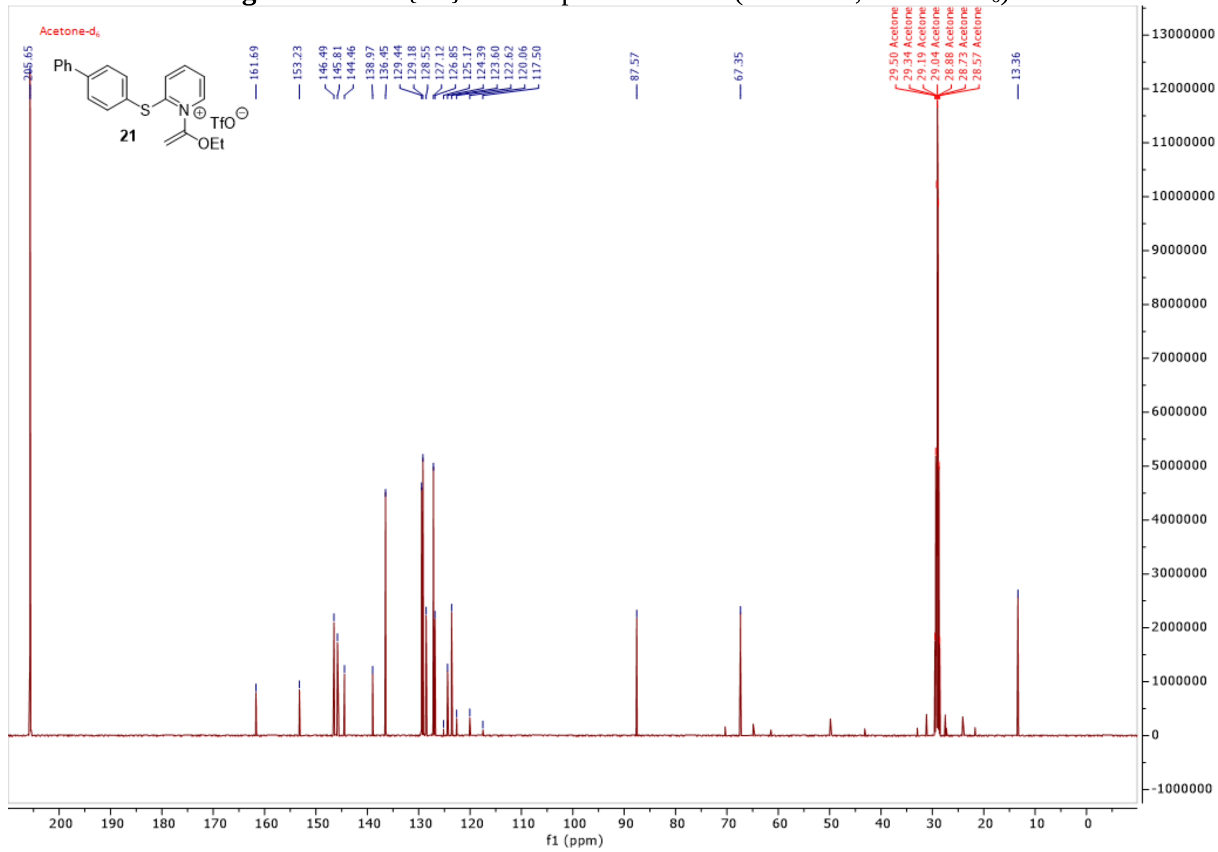

Figure S33. ^{1}H NMR Spectrum of **22** (500 MHz, Acetone-d$_6$)

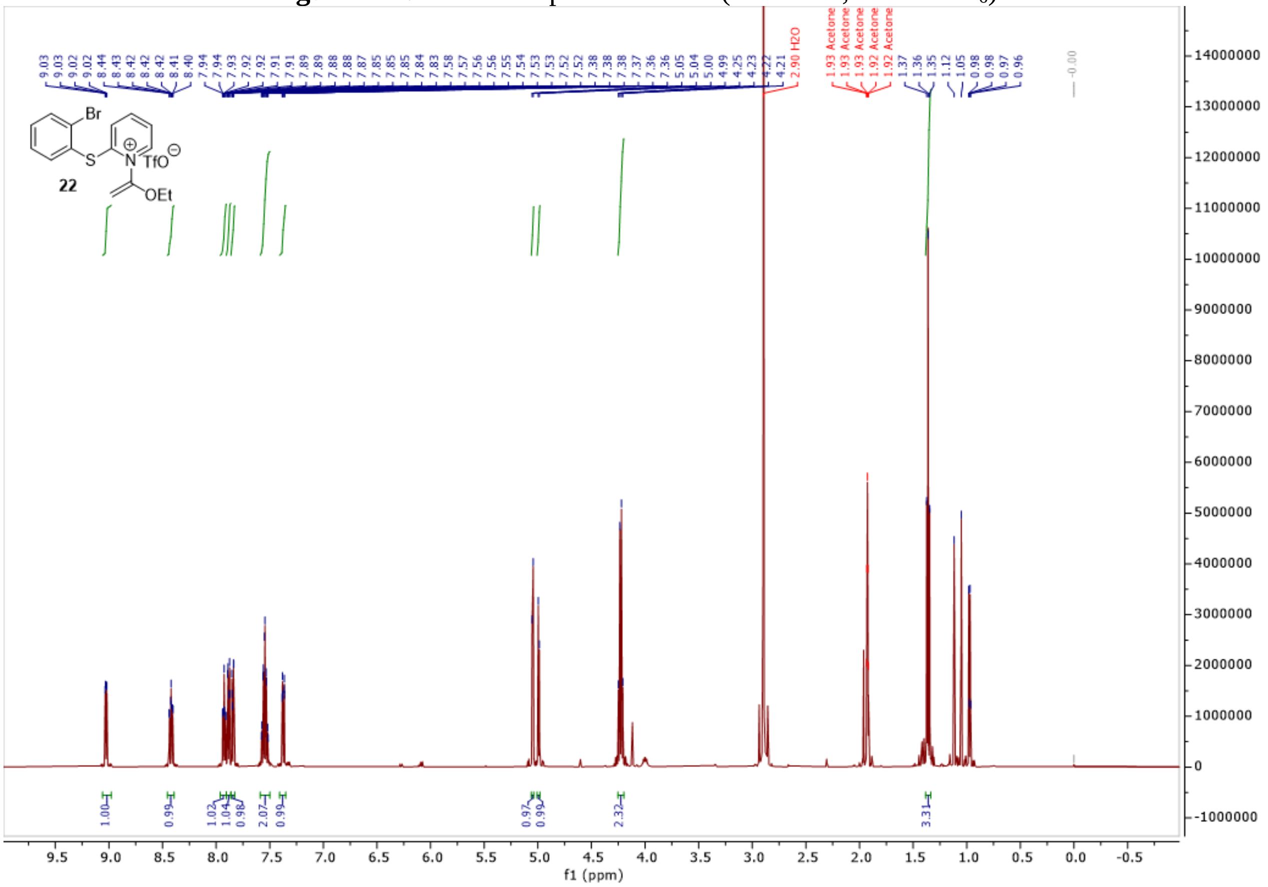

Figure S34. ^{13}C{^{1}H} NMR Spectrum of **22** (126 MHz, Acetone-d$_6$)

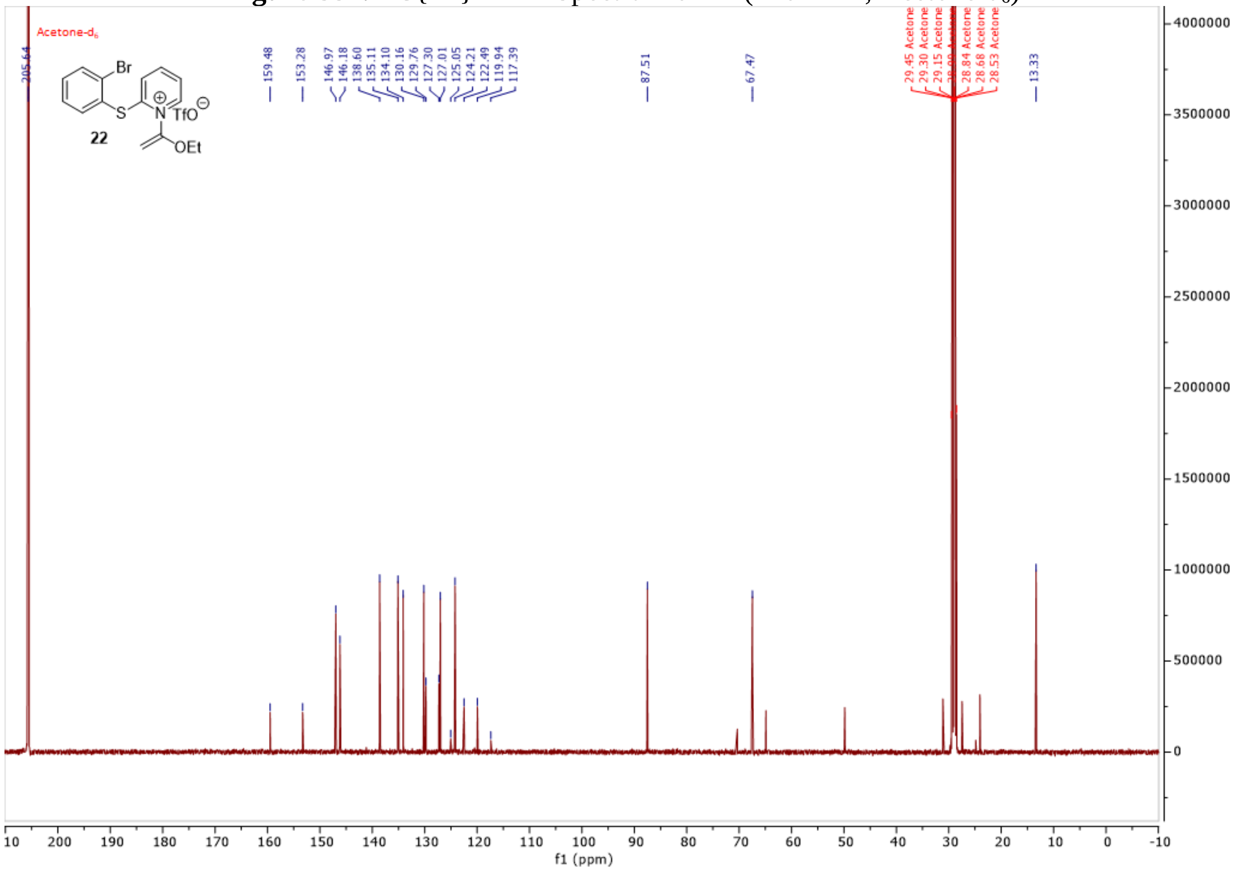

Figure S35. ^{1}H NMR Spectrum of **23** (500 MHz, Acetone-d_6)

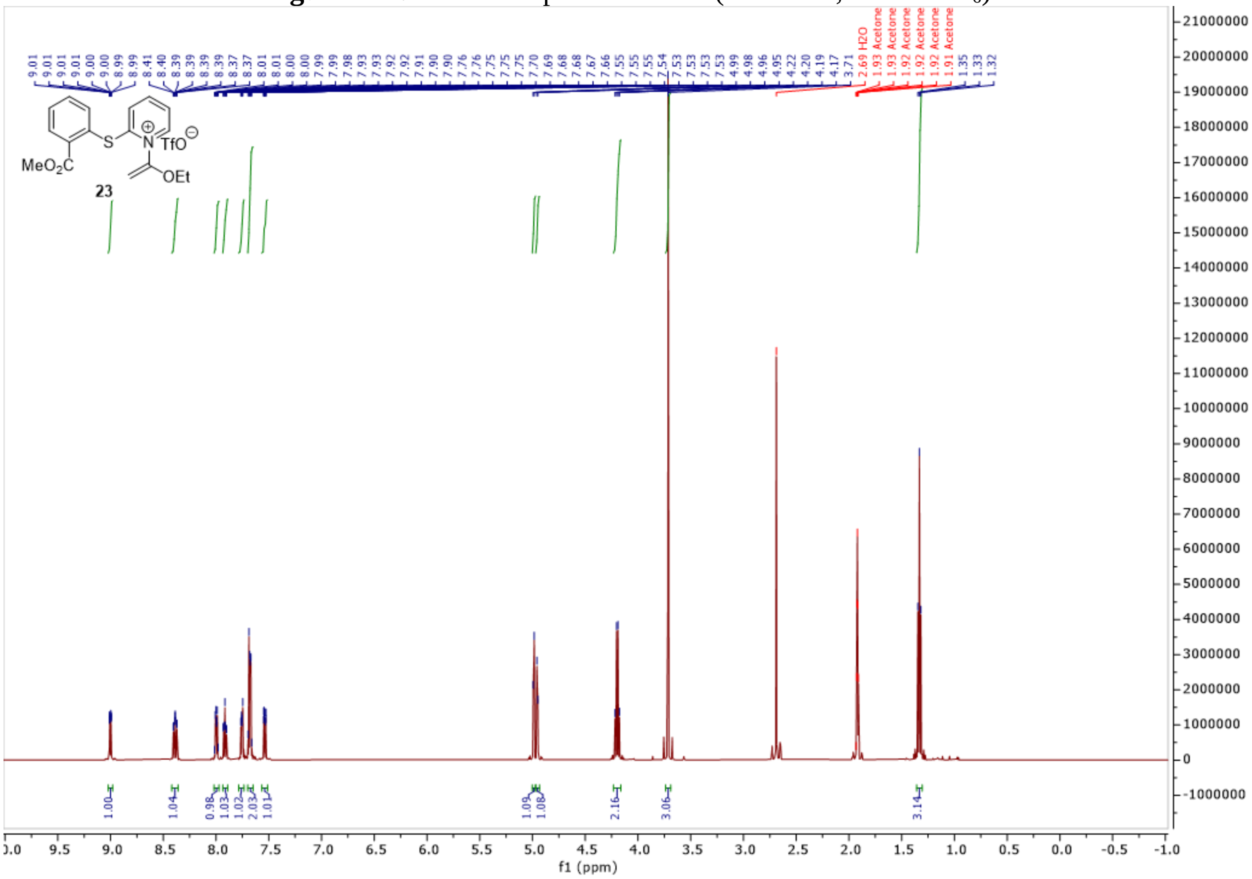

Figure S36. ^{13}C{^{1}H} NMR Spectrum of **23** (126 MHz, Acetone-d_6)

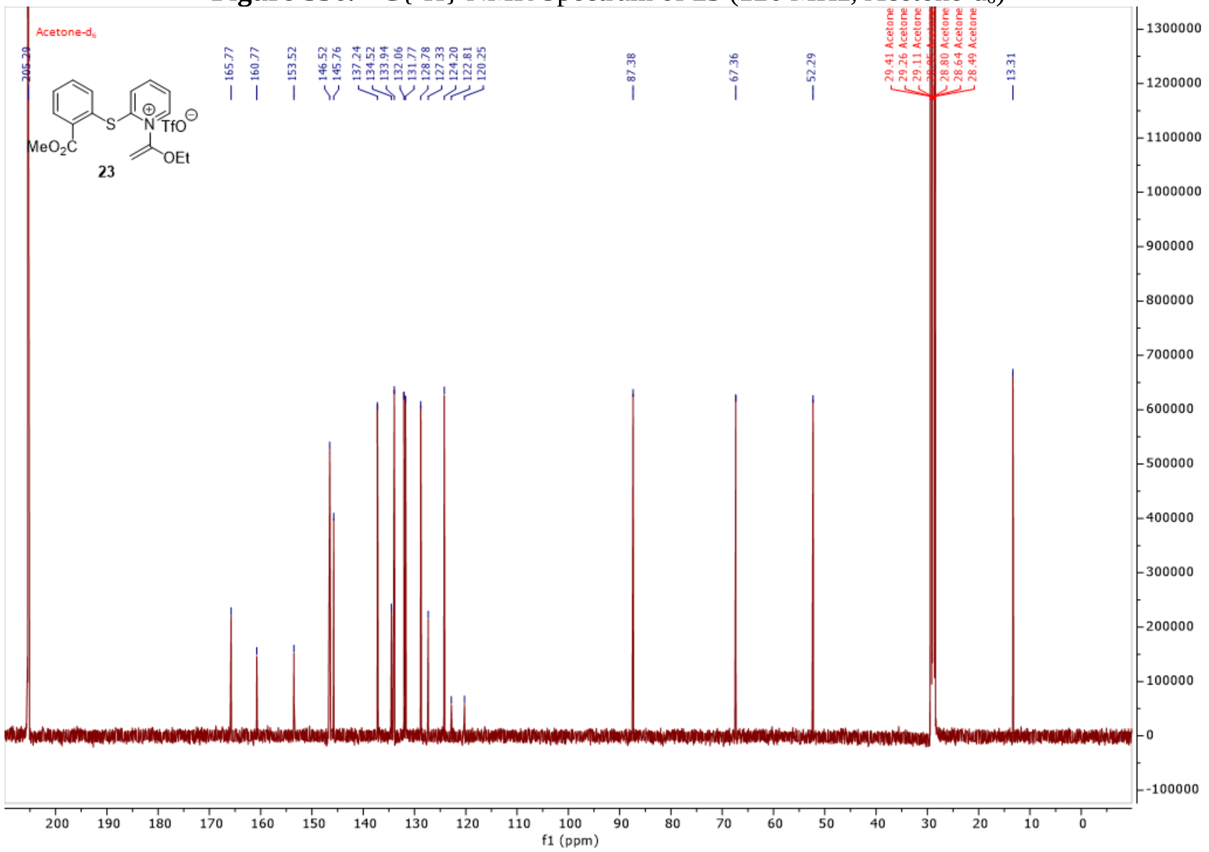

Figure S37. ^{1}H NMR Spectrum of **25** (500 MHz, Acetone-d$_6$)

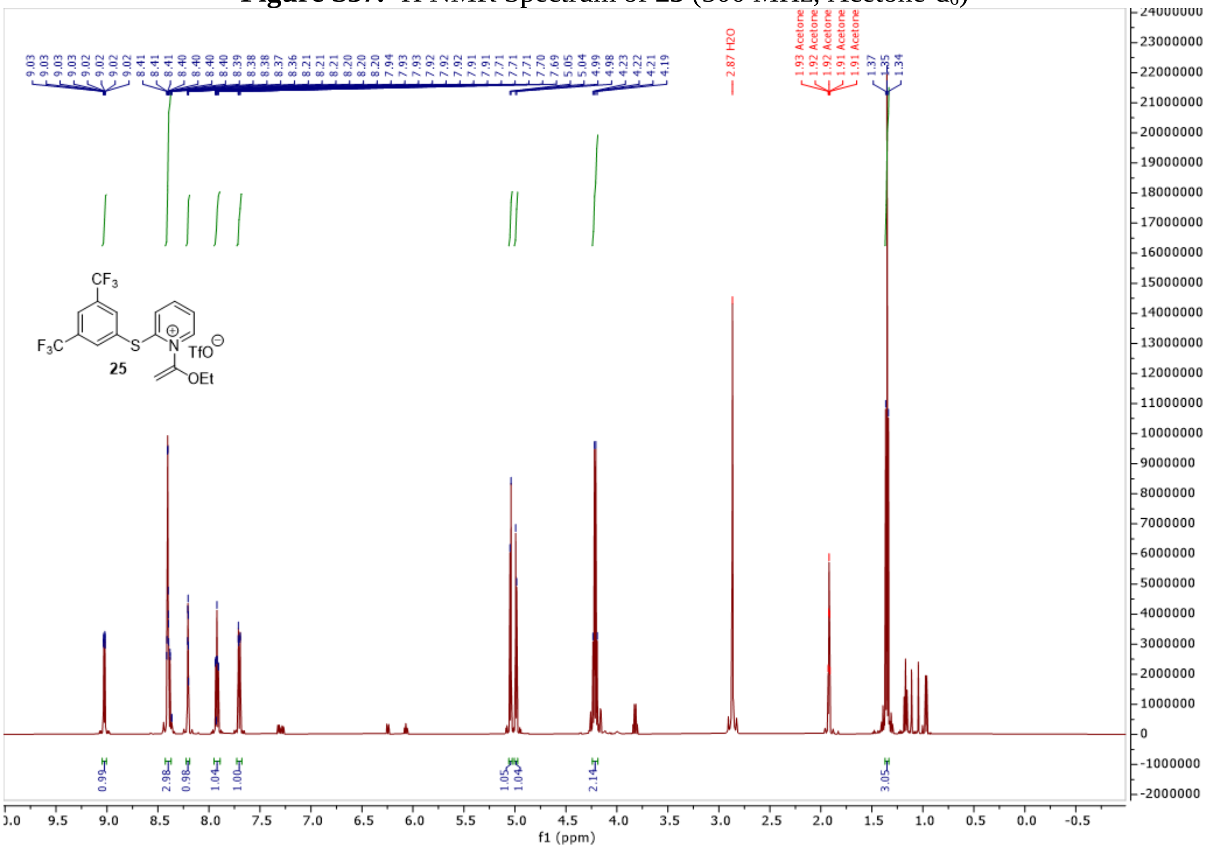

Figure S38. ^{13}C{^{1}H} NMR Spectrum of **25** (126 MHz, Acetone-d$_6$)

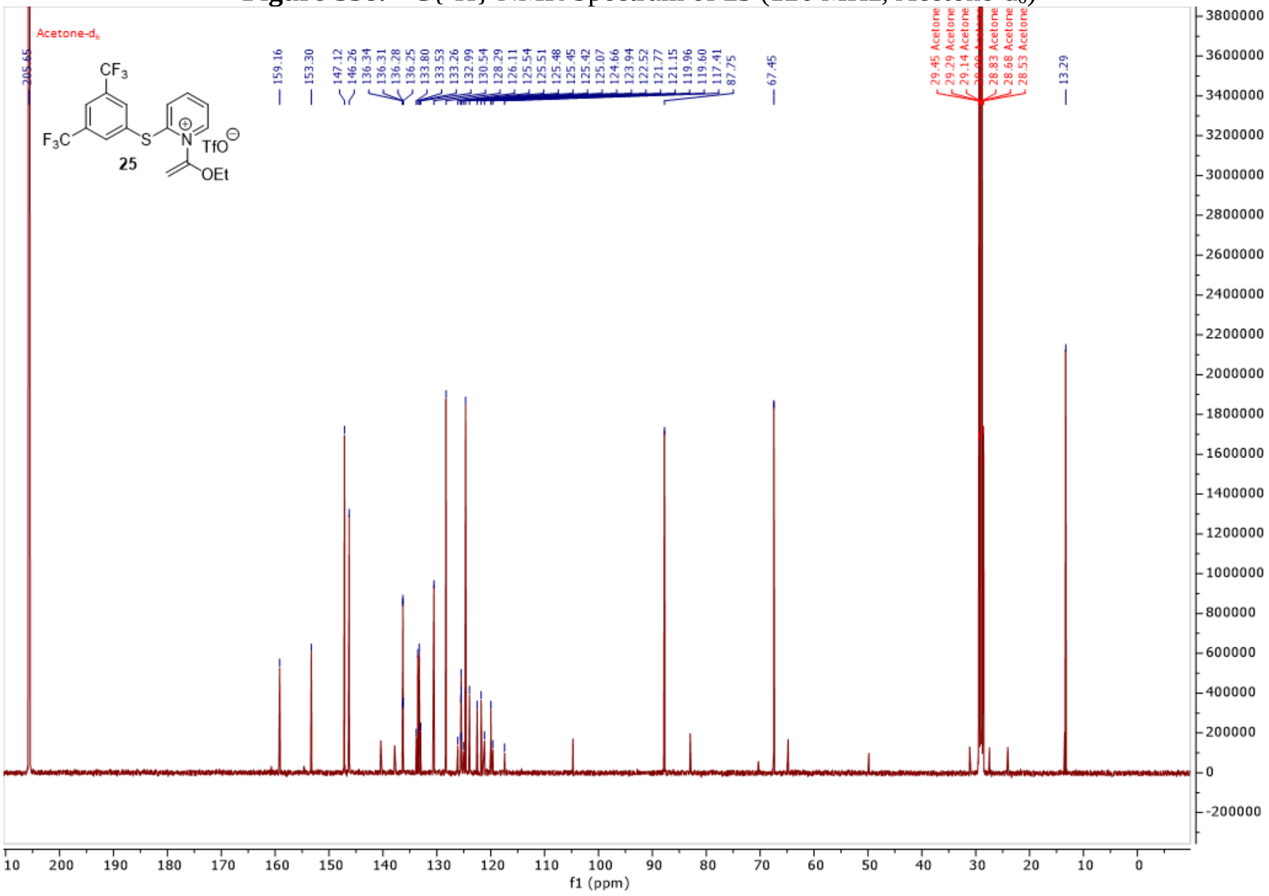

Figure S39. ^{1}H NMR Spectrum of **26** (500 MHz, Acetone-d_6)

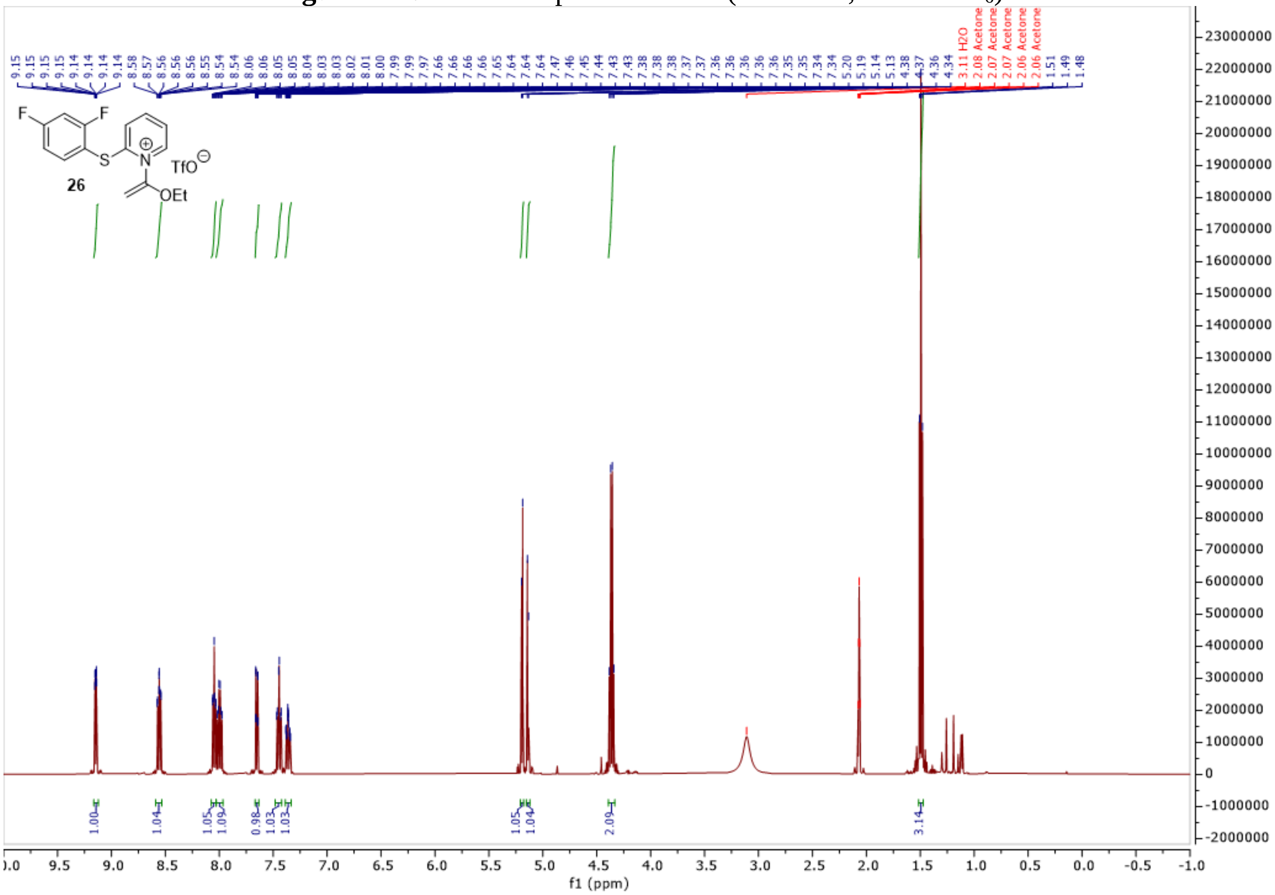

Figure S40. $^{13}C\{^{1}H\}$ NMR Spectrum of **26** (126 MHz, Acetone-d_6)

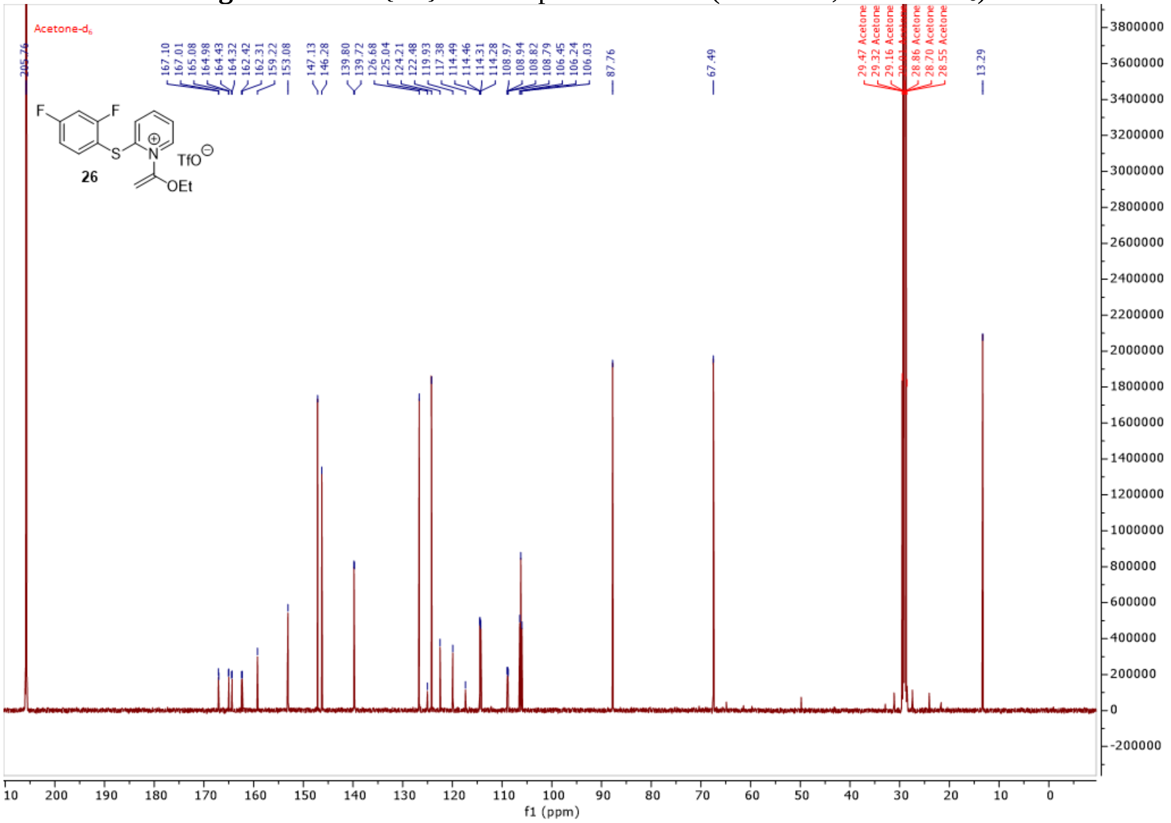

Figure S41. ^{1}H NMR Spectrum of **27** (500 MHz, Acetone-d$_6$)

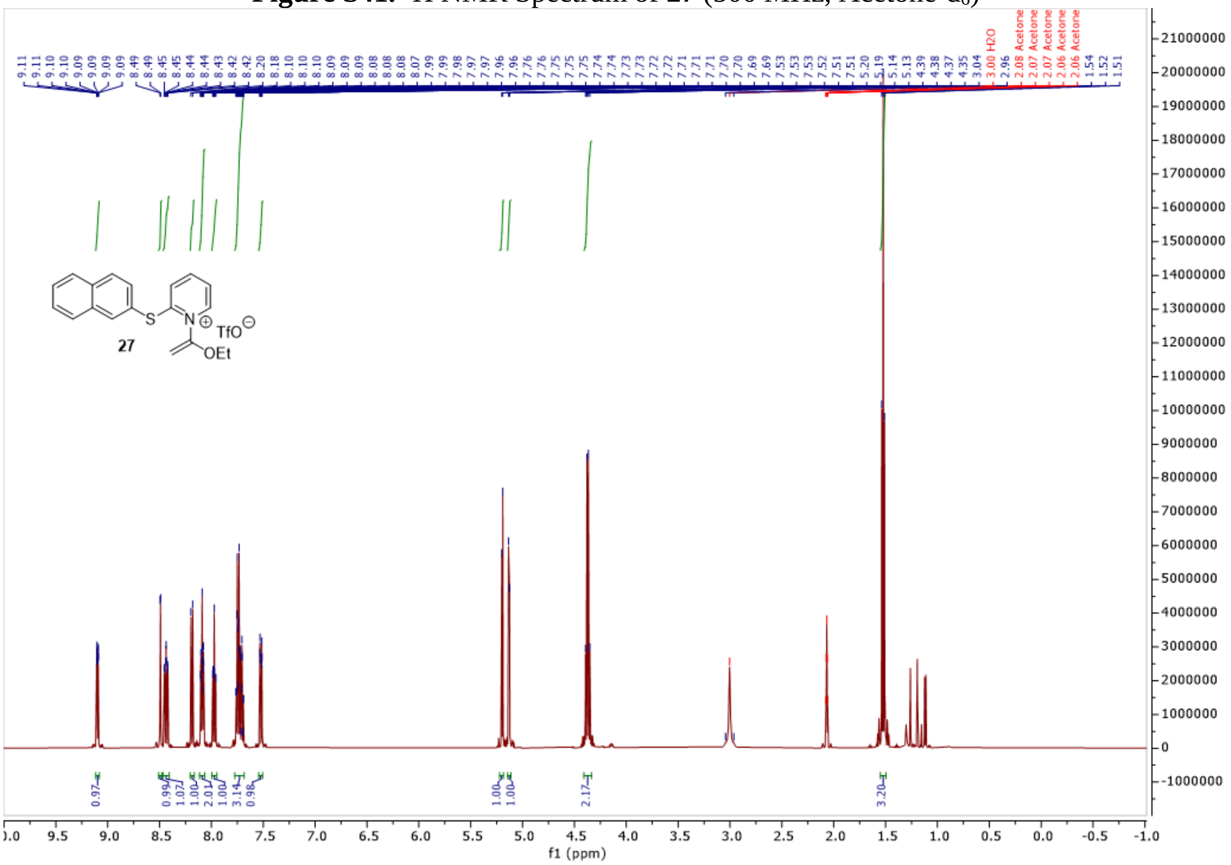

Figure S42. ^{13}C{^{1}H} NMR Spectrum of **27** (126 MHz, Acetone-d$_6$)

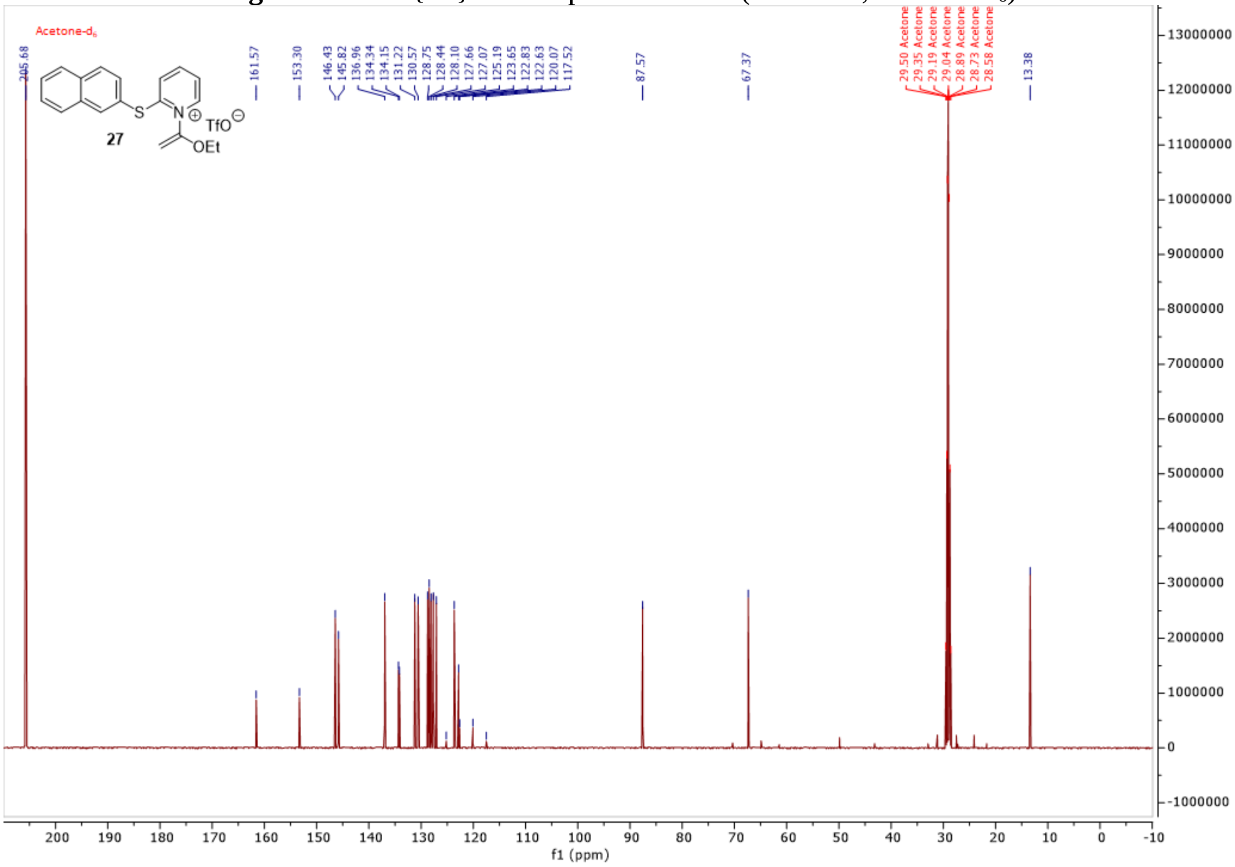

Figure S43. ^{1}H NMR Spectrum of **28** (500 MHz, Acetone-d$_6$)

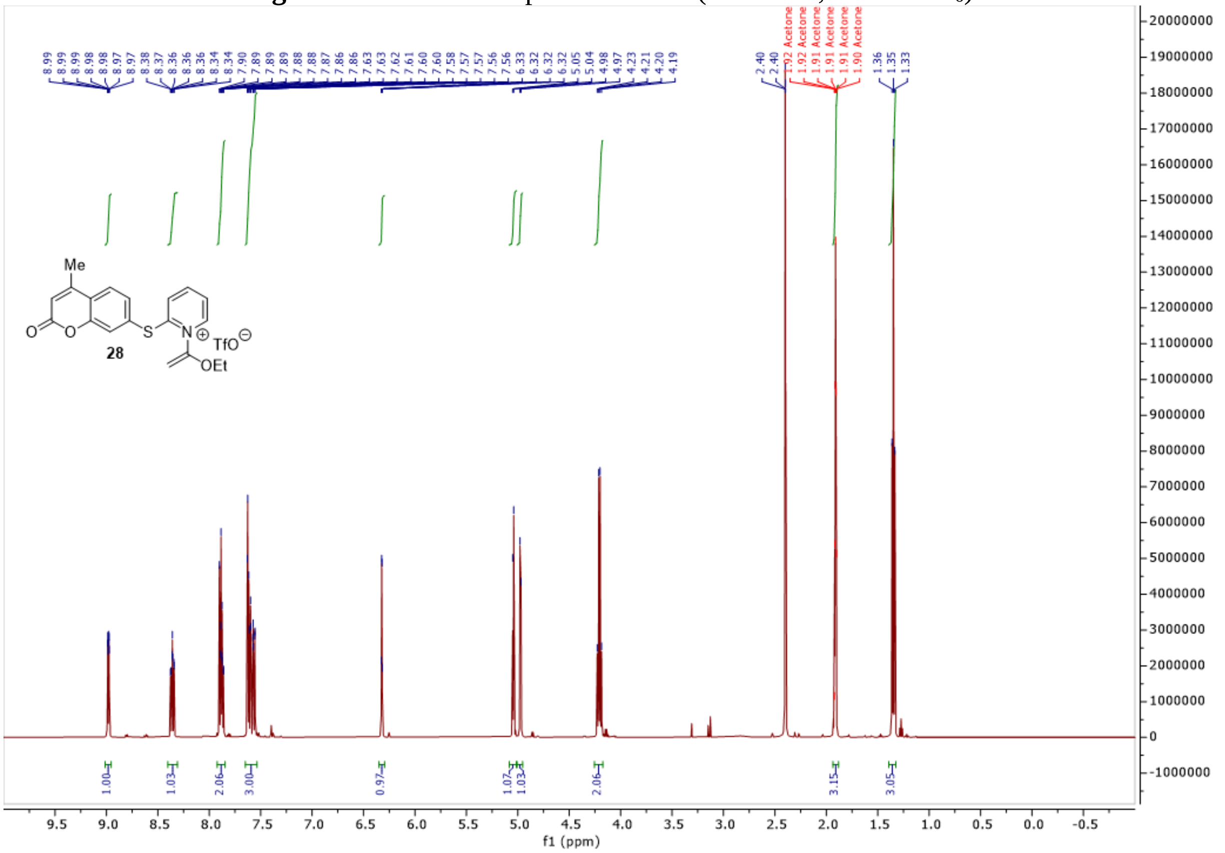

Figure S44. ^{13}C{^{1}H} NMR Spectrum of **28** (126 MHz, Acetone-d$_6$)

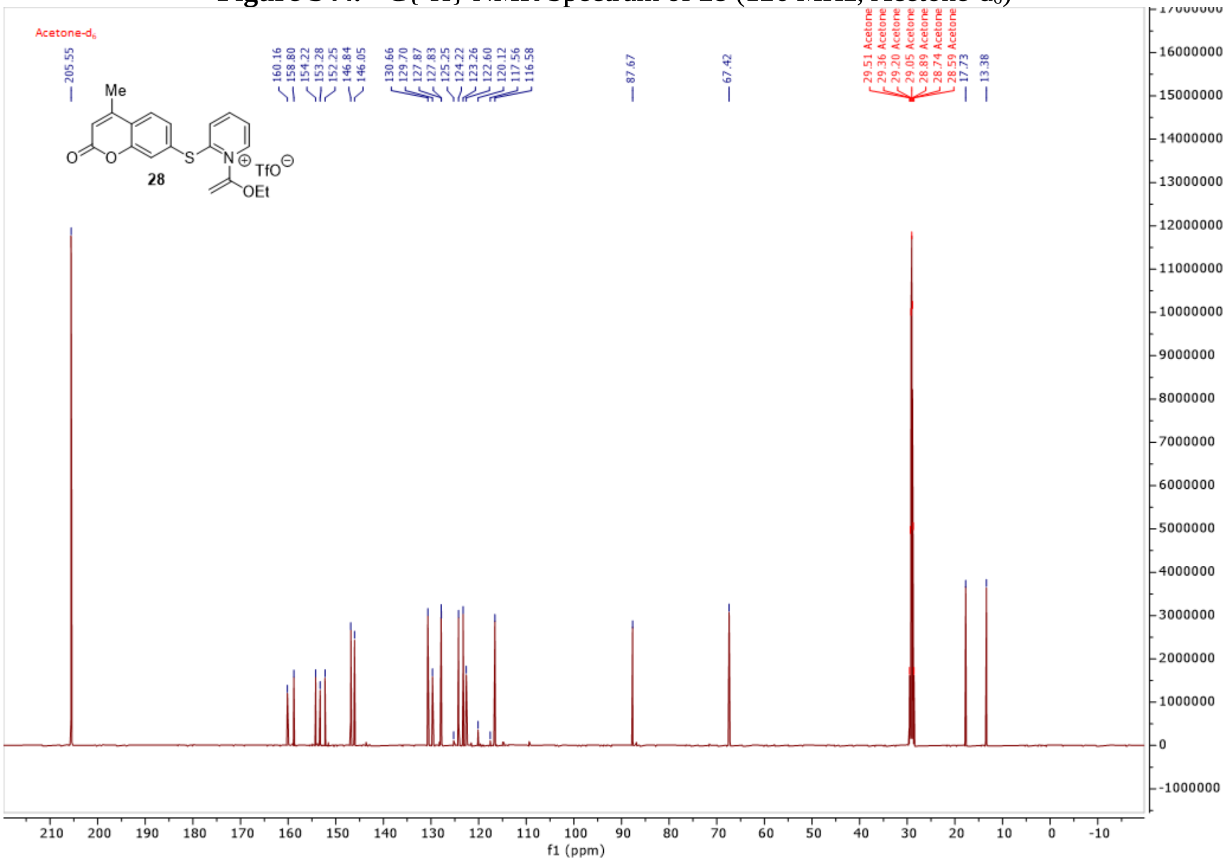

Figure S45. ^{1}H NMR Spectrum of **29** (500 MHz, Acetone-d_6)

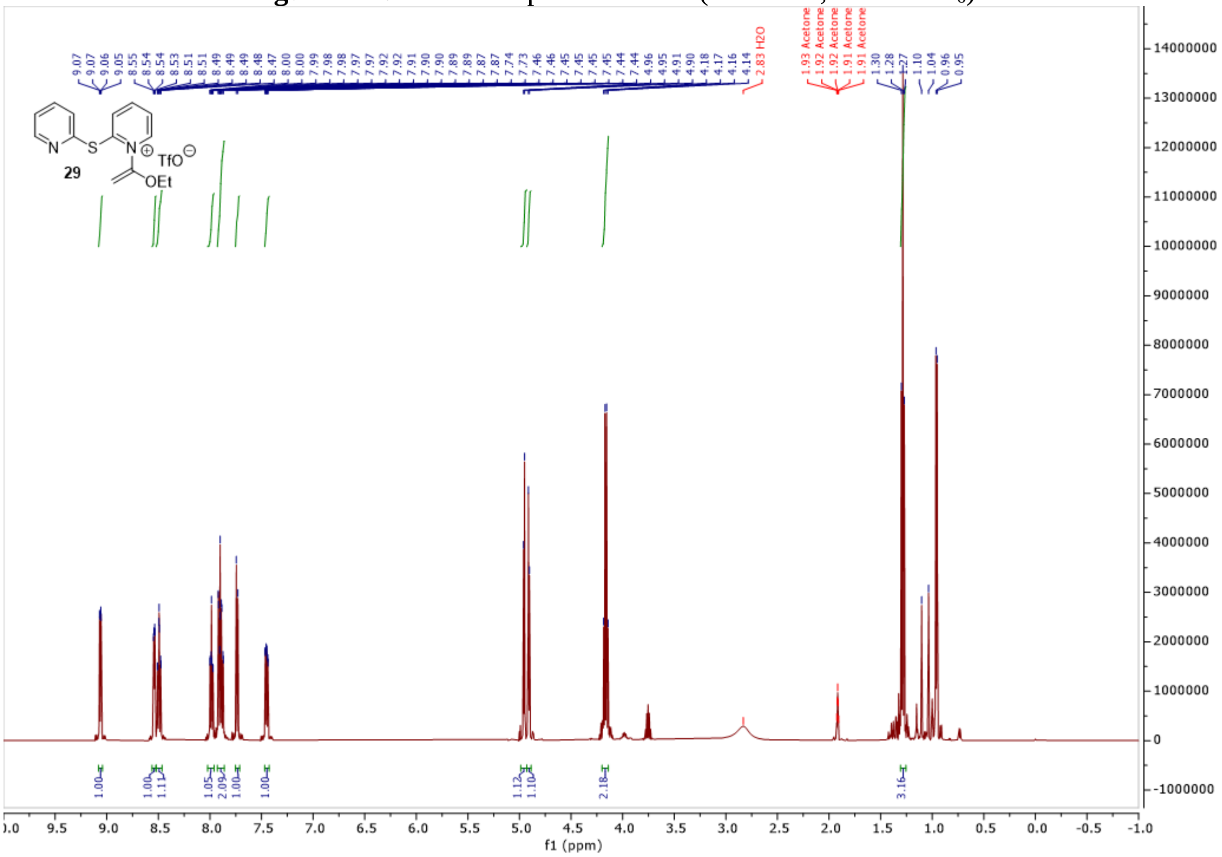

Figure S46. ^{13}C{^{1}H} NMR Spectrum of **29** (126 MHz, Acetone-d_6)

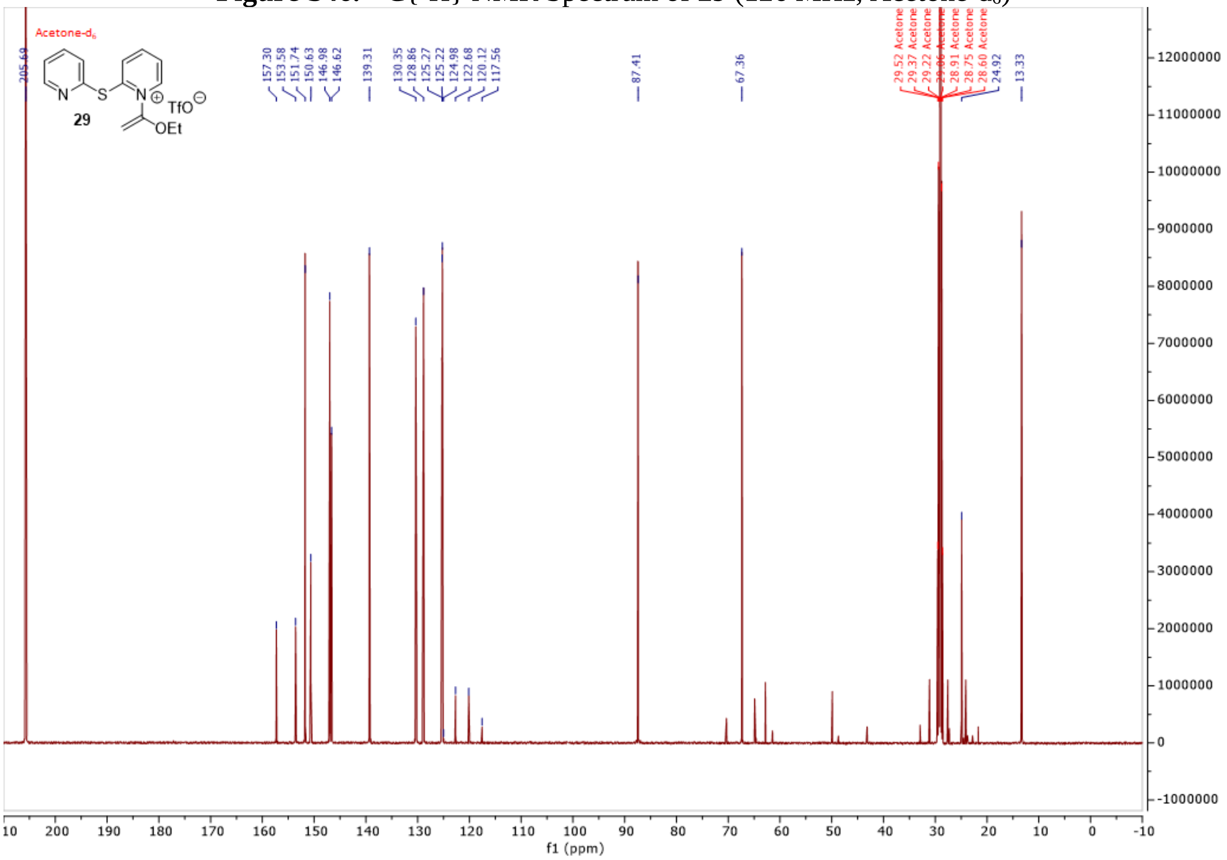

Figure S47. ^{1}H NMR Spectrum of **30** (500 MHz, Acetone-d$_6$)

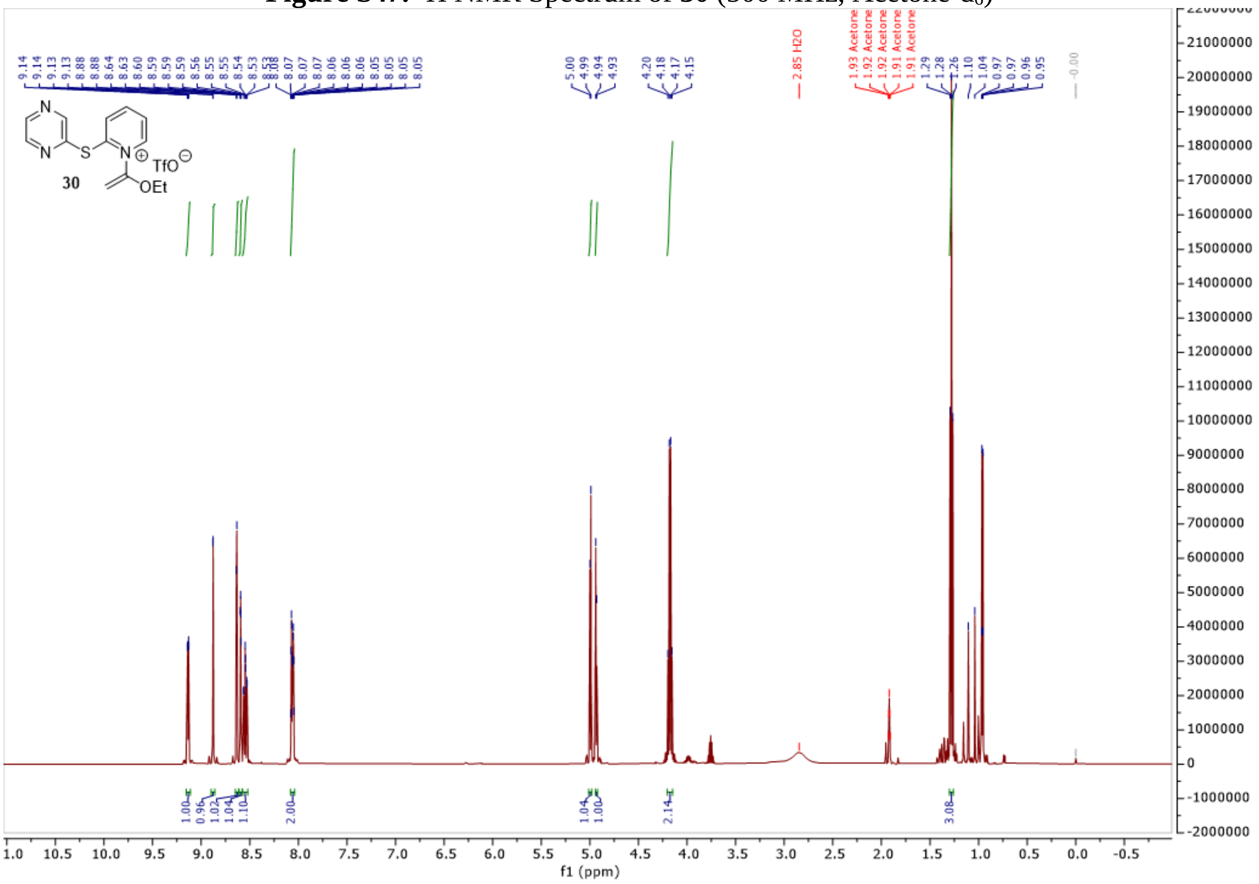

Figure S48. ^{13}C{^{1}H} NMR Spectrum of **30** (126 MHz, Acetone-d$_6$)

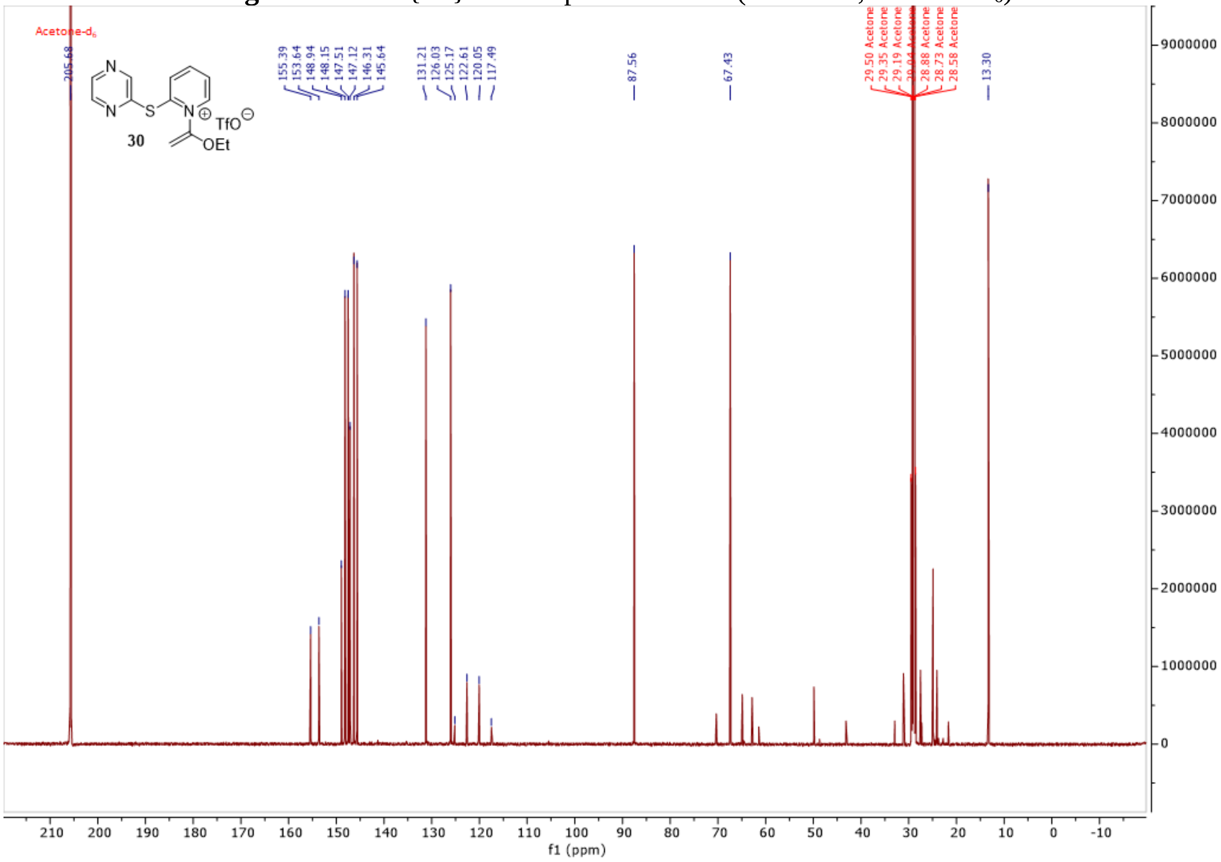

Figure S49. ^{1}H NMR Spectrum of **31** (500 MHz, Acetone-d_6)

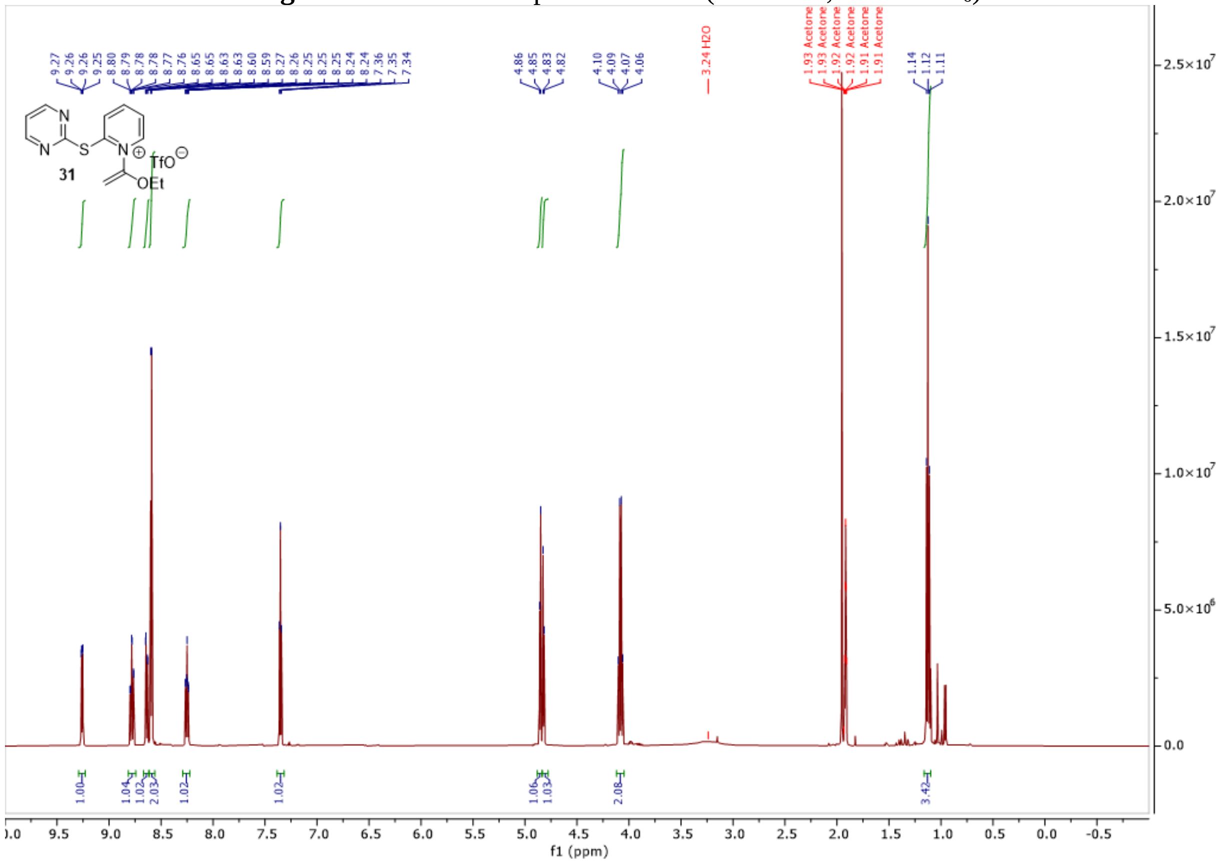

Figure S50. ^{13}C{^{1}H} NMR Spectrum of **31** (126 MHz, Acetone-d_6)

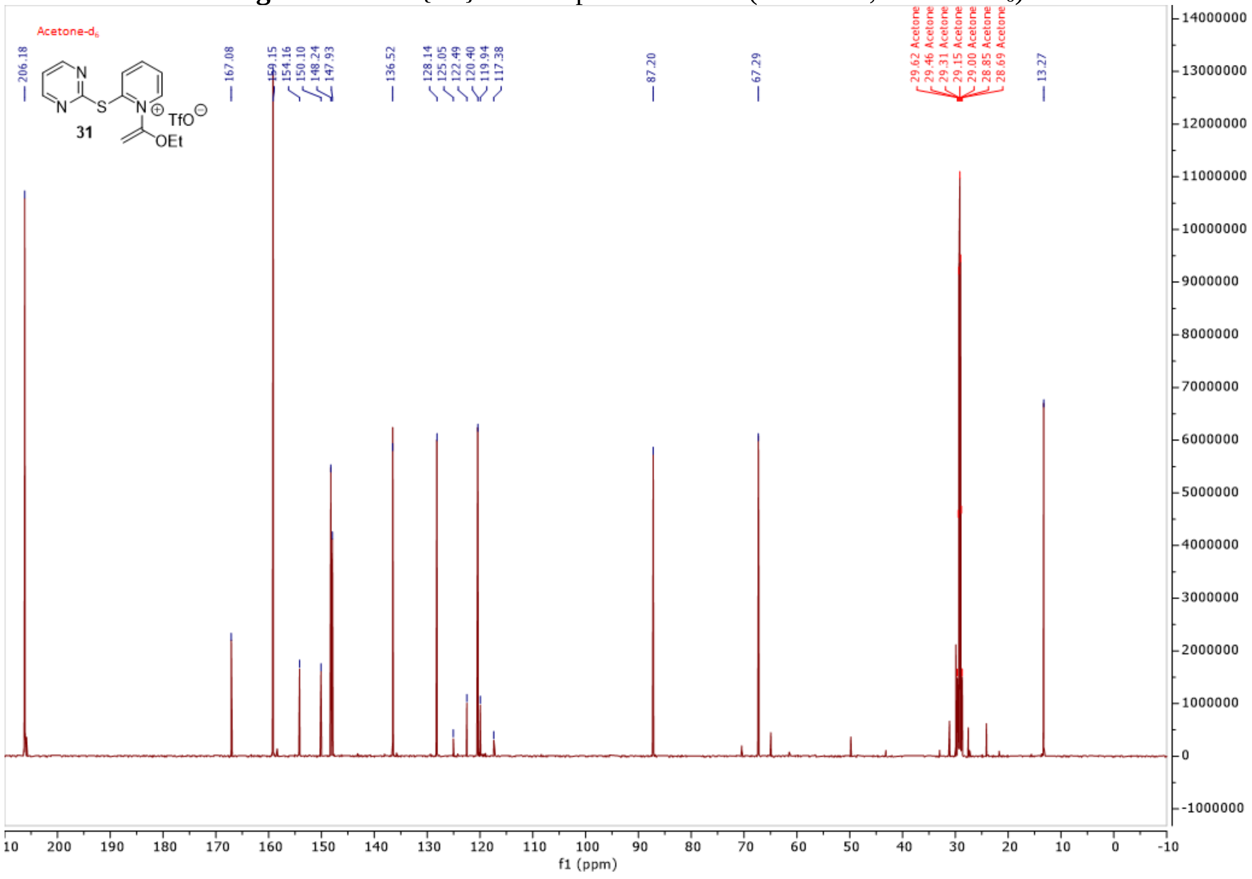

Figure S51. ^{1}H NMR Spectrum of **32** (500 MHz, Acetone-d_6)

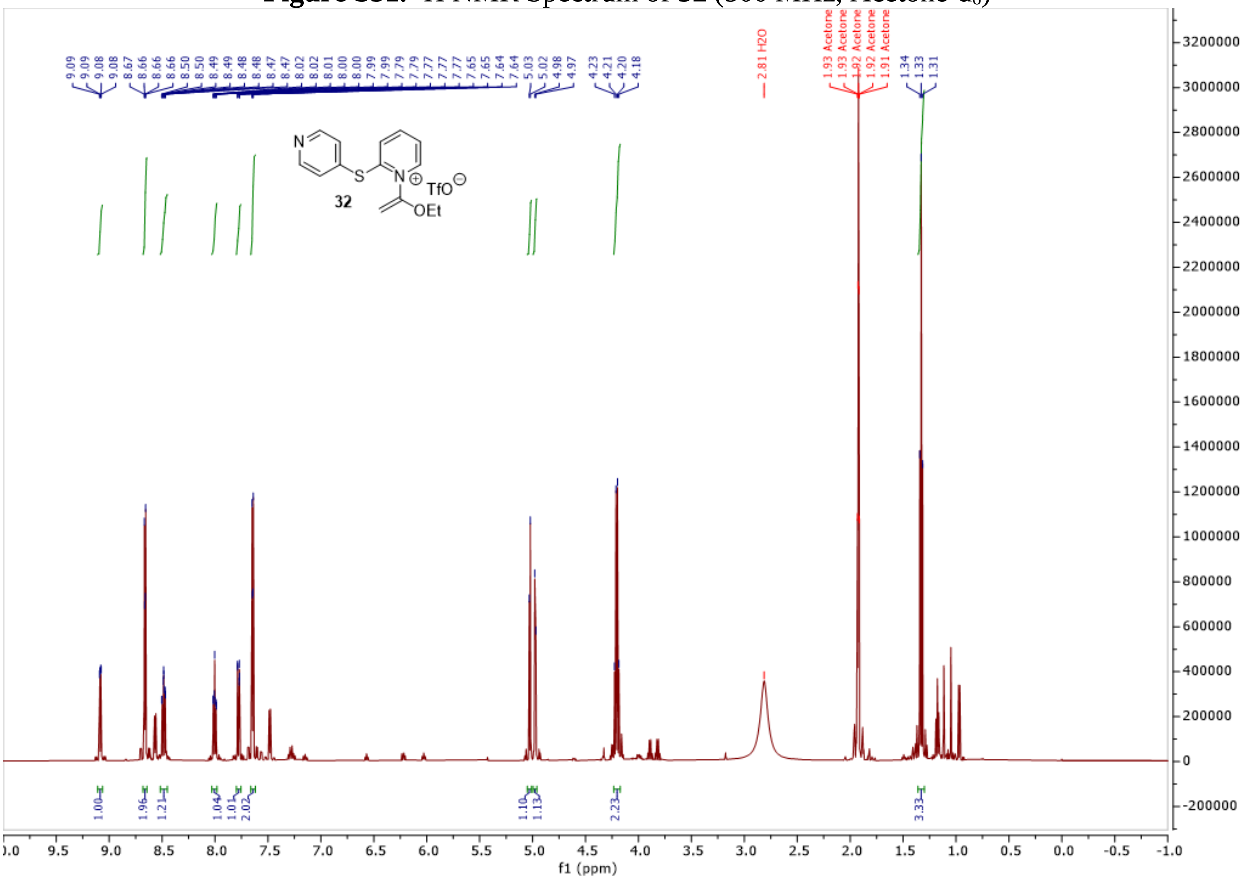

Figure S52. $^{13}C\{^{1}H\}$ NMR Spectrum of **32** (126 MHz, Acetone-d_6)

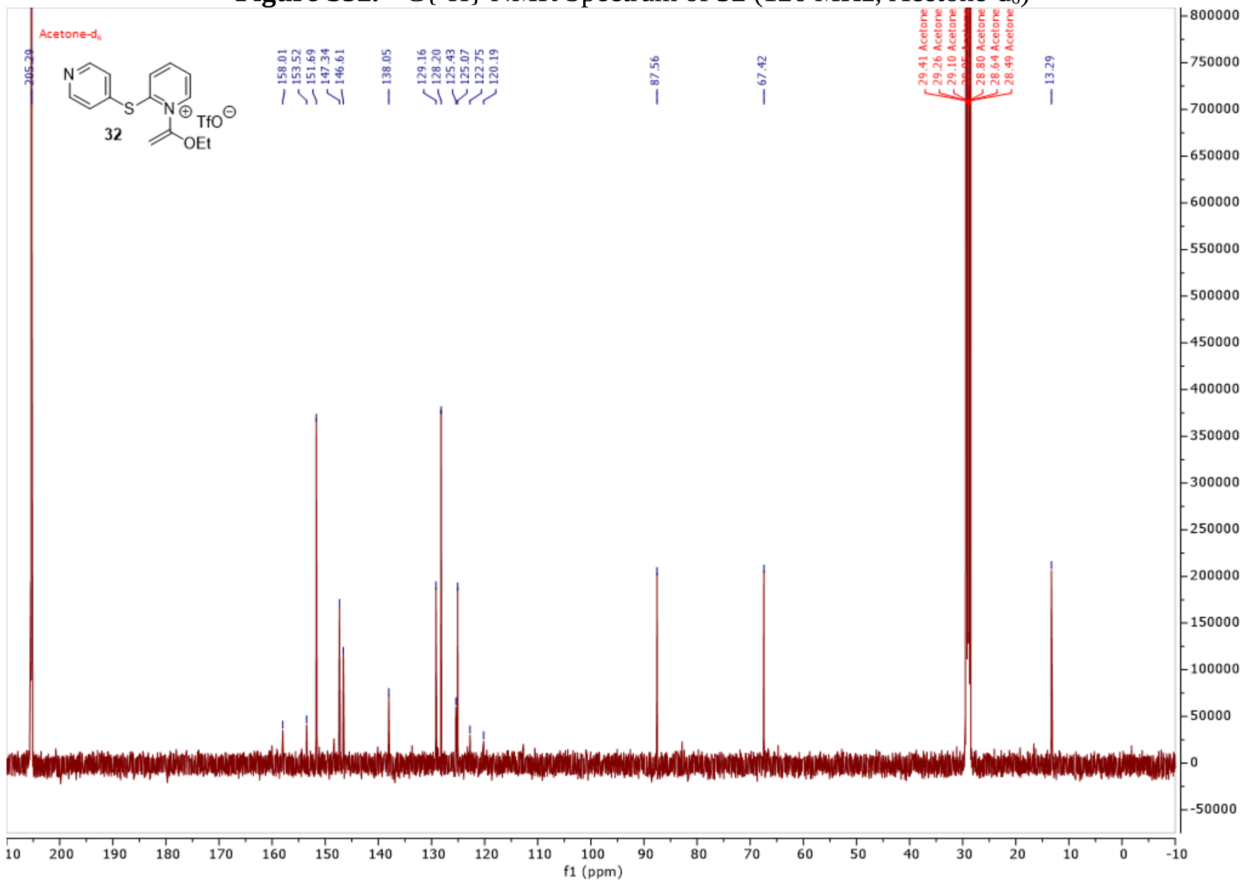

Figure S53. ^{1}H NMR Spectrum of **33** (500 MHz, $CDCl_3$)

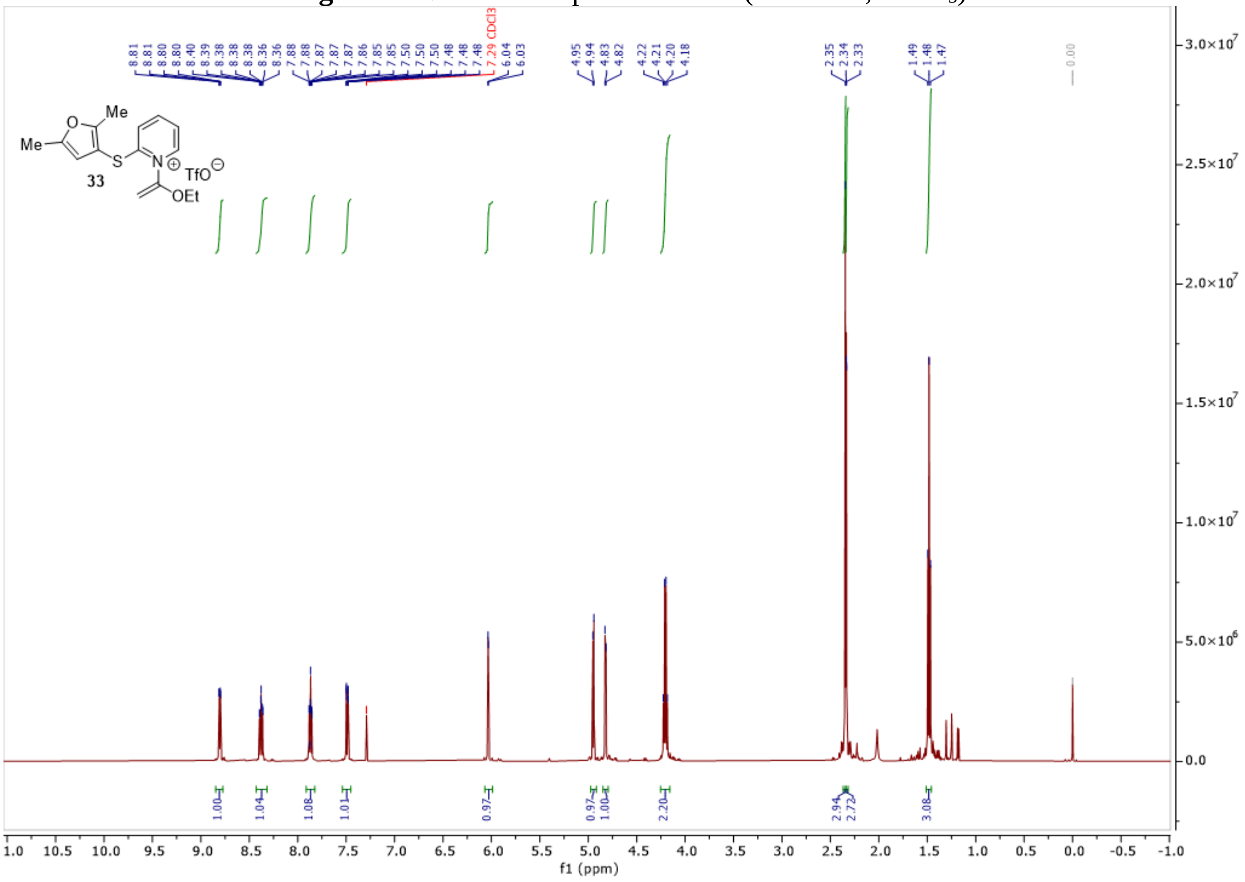

Figure S54. $^{13}C\{^{1}H\}$ NMR Spectrum of **33** (126 MHz, $CDCl_3$)

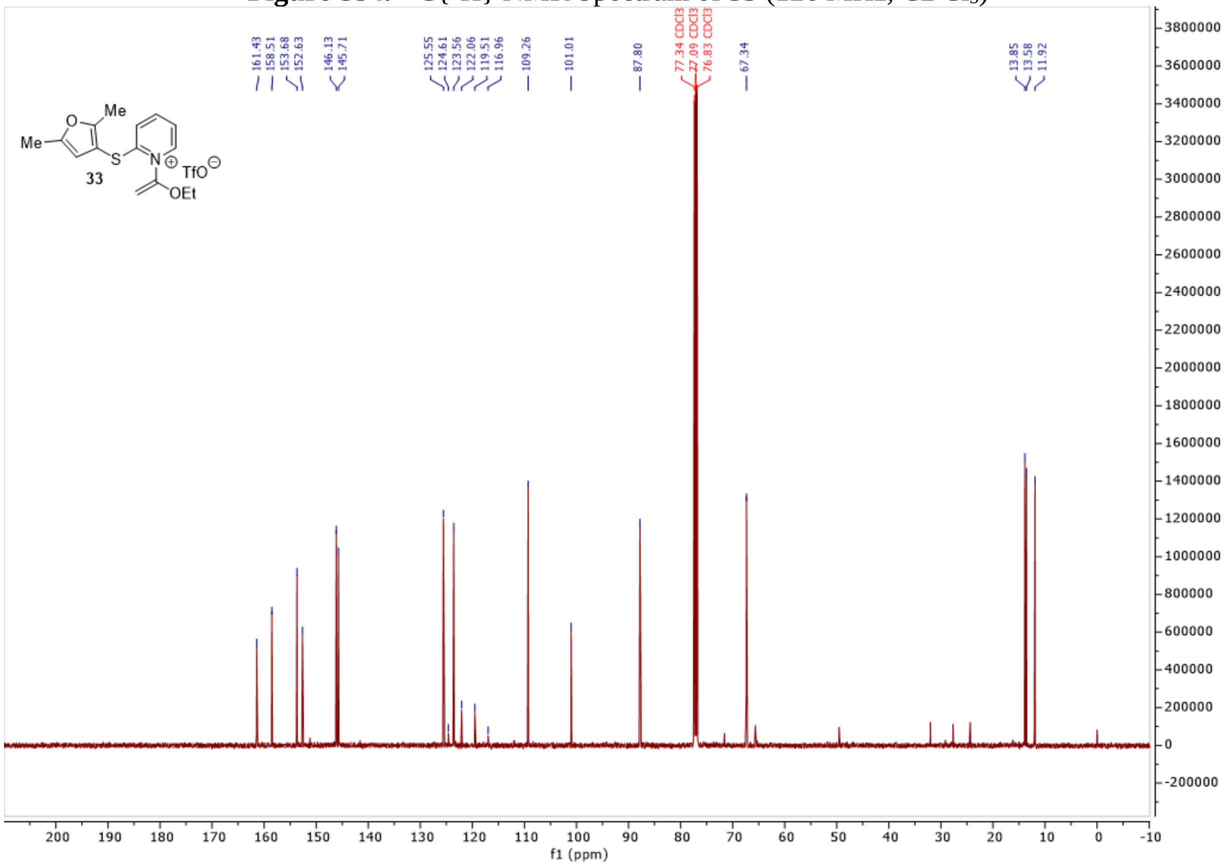

Figure S55. ^{1}H NMR Spectrum of **34** (500 MHz, Acetone-d_6)

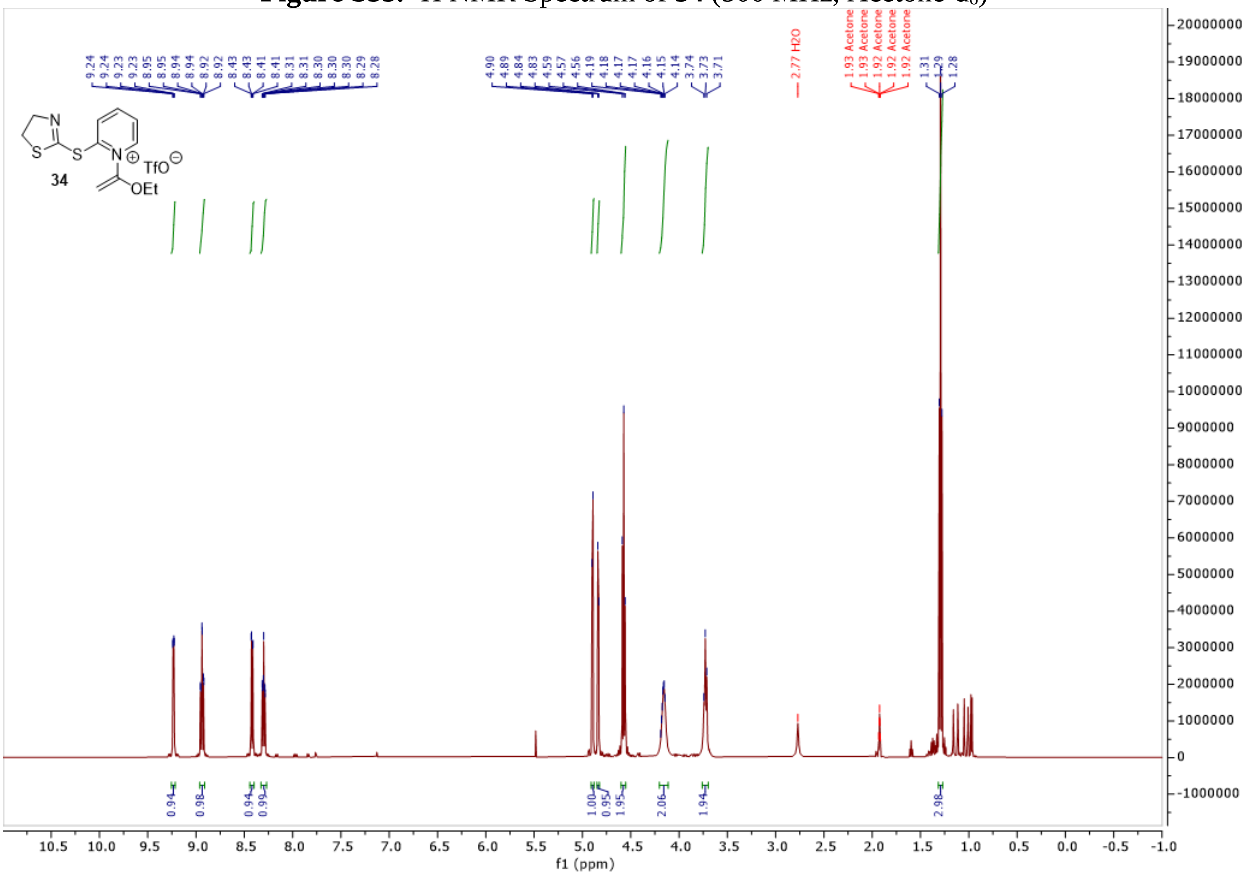

Figure S56. ^{13}C{^{1}H} NMR Spectrum of **34** (126 MHz, Acetone-d_6)

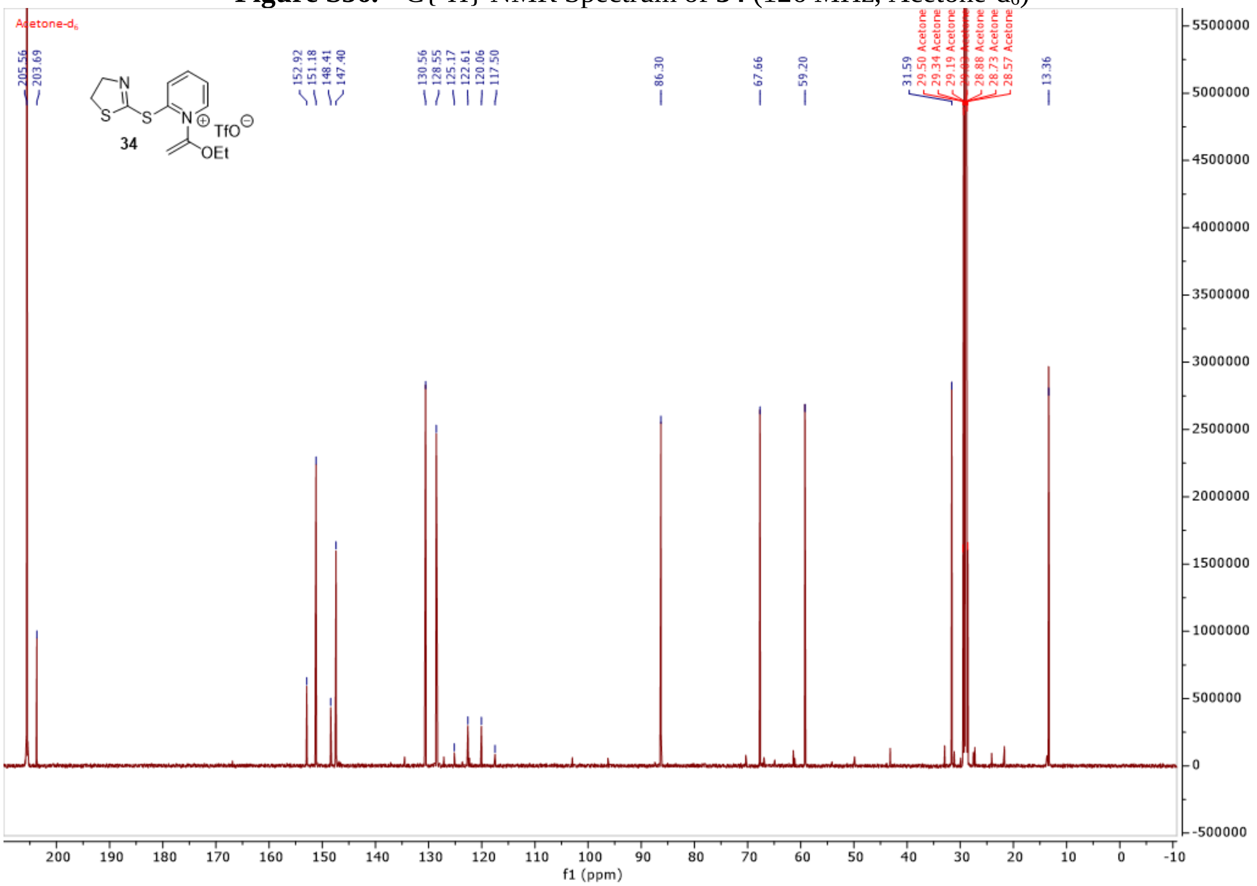

Figure S57. ^{1}H NMR Spectrum of **36** (500 MHz, Acetone-d_6)

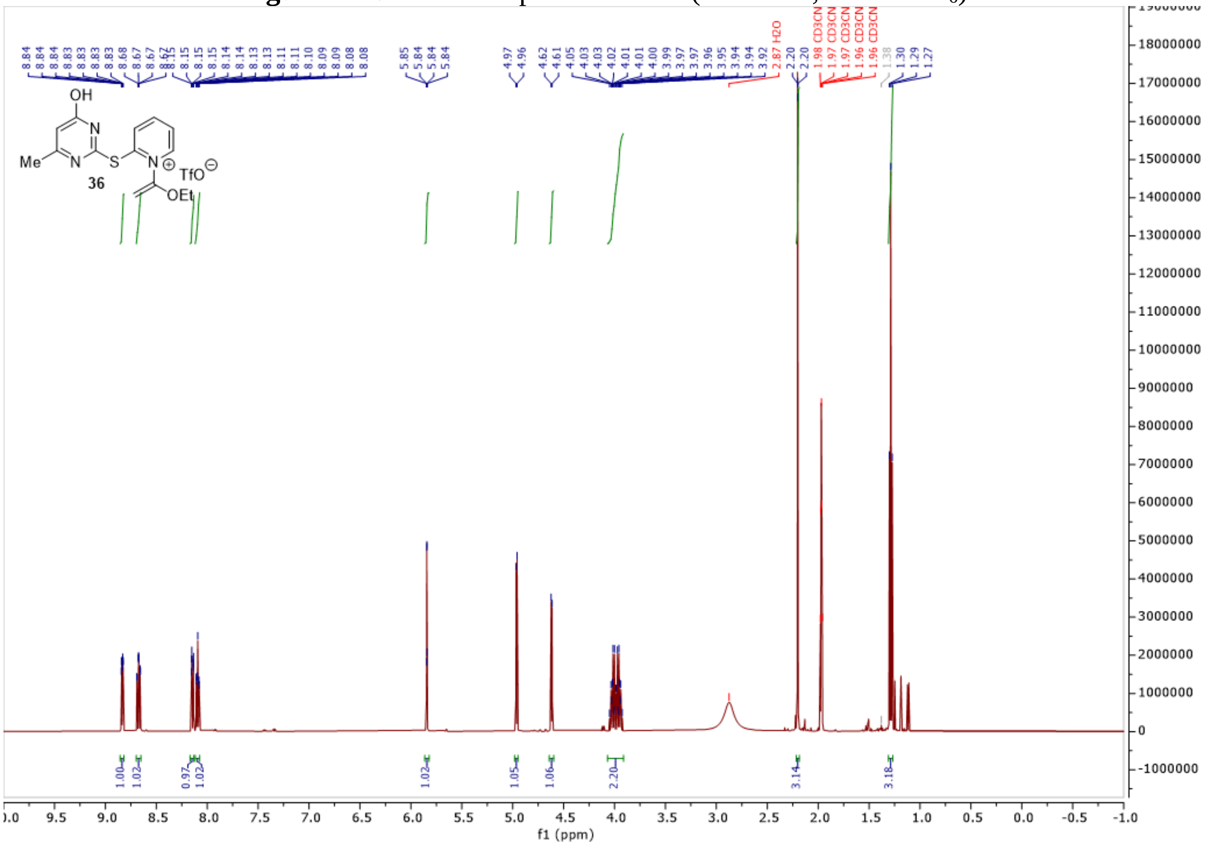

Figure S58. $^{13}C\{^{1}H\}$ NMR Spectrum of **36** (126 MHz, Acetone-d_6)

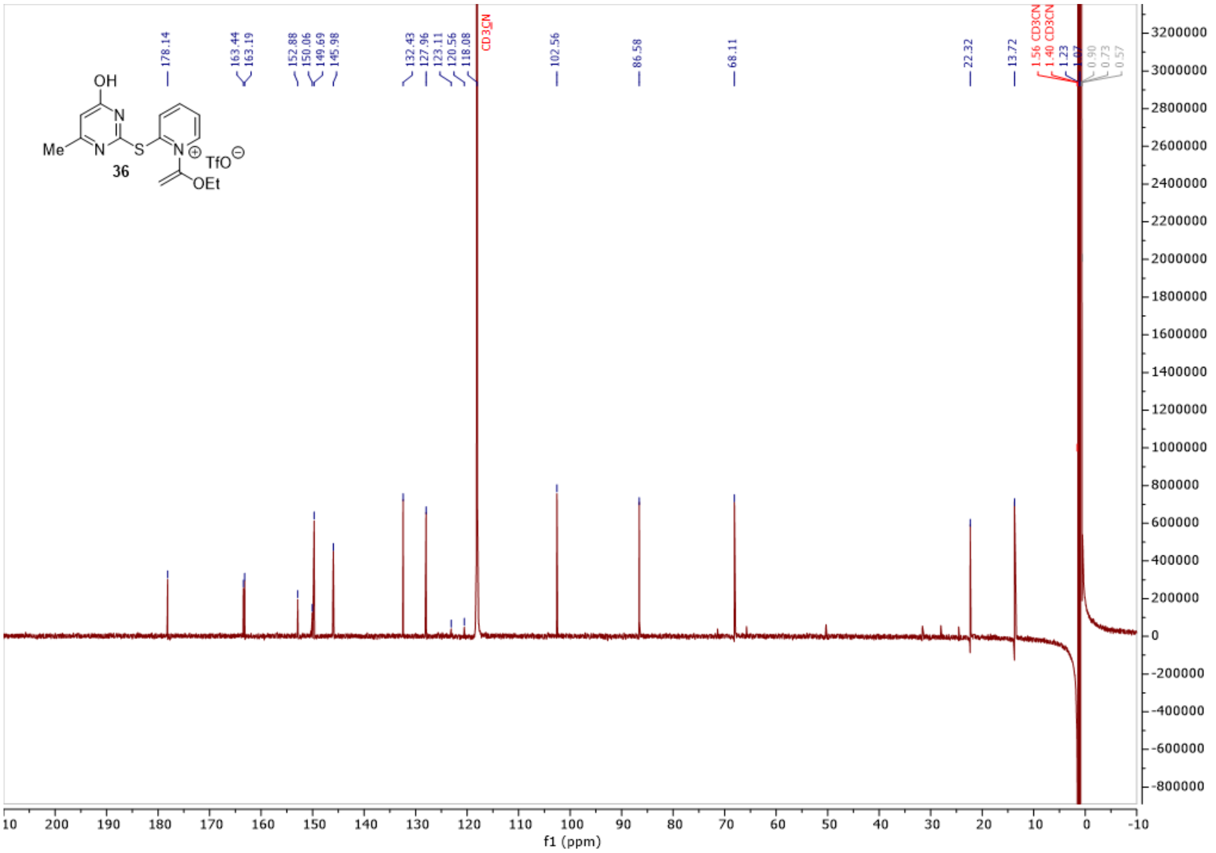

Figure S59. ^{1}H NMR Spectrum of **37** (500 MHz, Acetone-d_6)

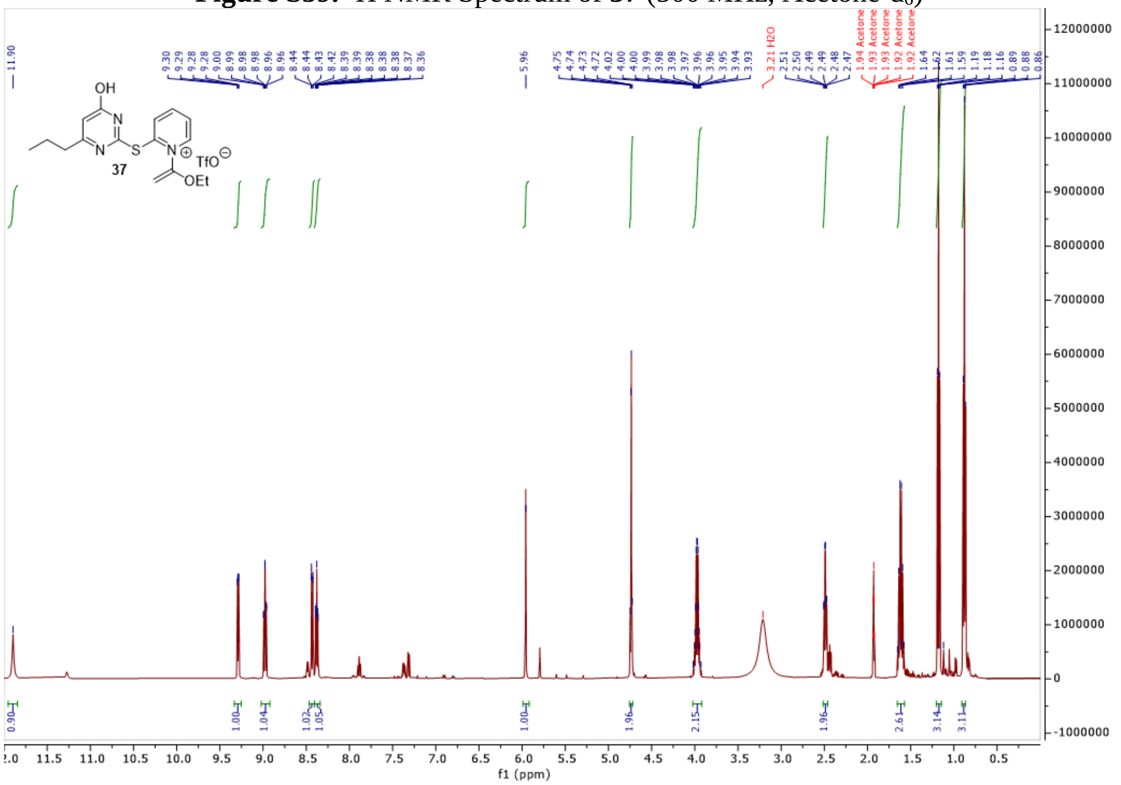

Figure S60. ^{13}C{^{1}H} NMR Spectrum of **37** (126 MHz, Acetone-d_6)

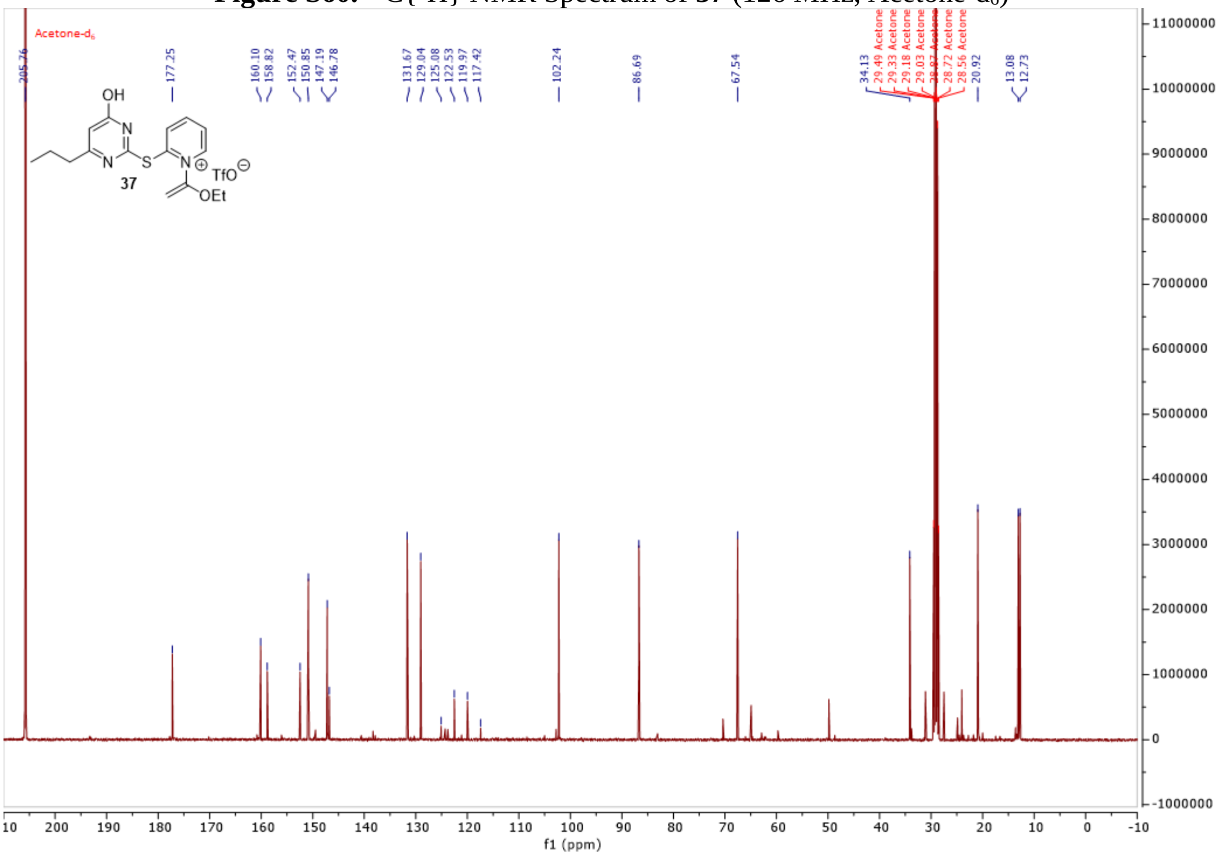

Figure S61. ^{1}H NMR Spectrum of **39** (500 MHz, Acetone-d_6)

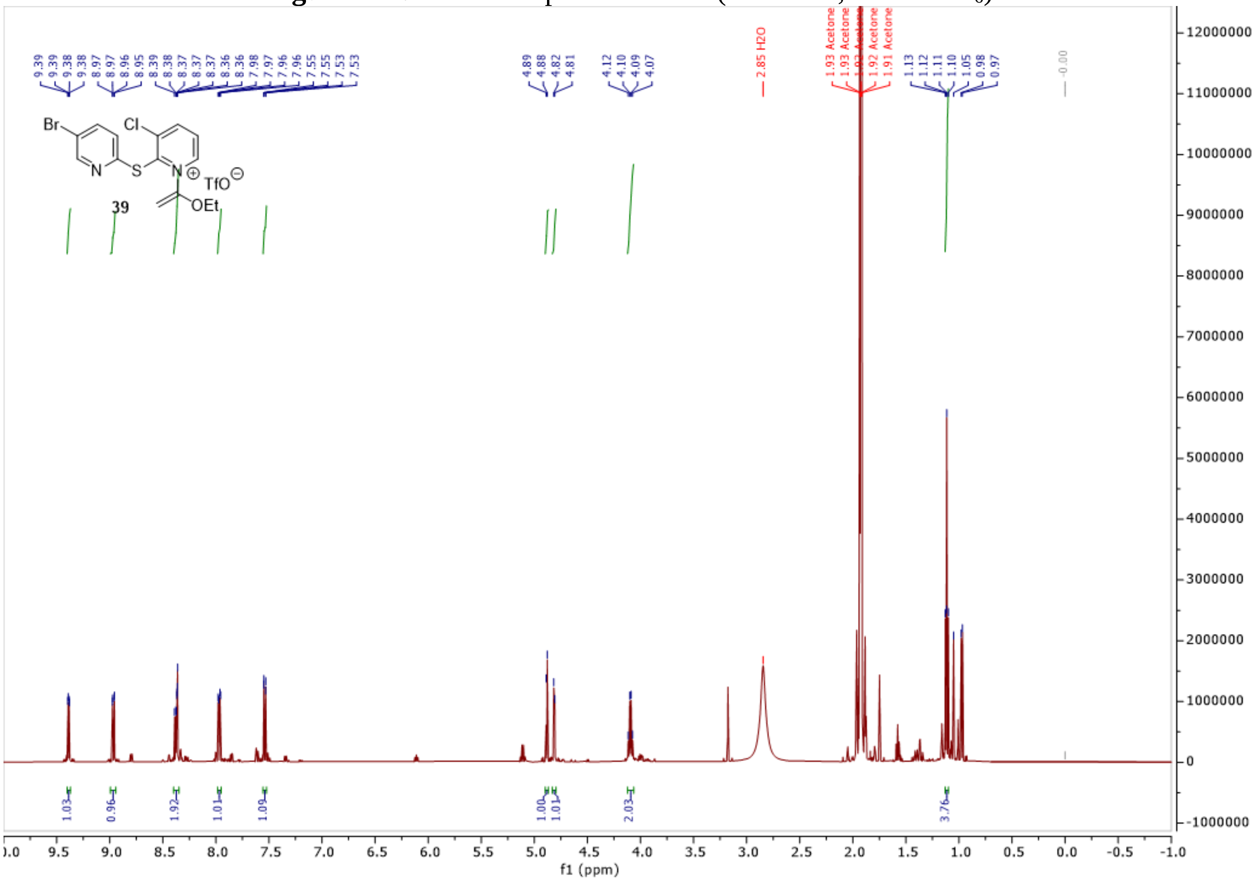

Figure S62. $^{13}C\{^{1}H\}$ NMR Spectrum of **39** (126 MHz, Acetone-d_6)

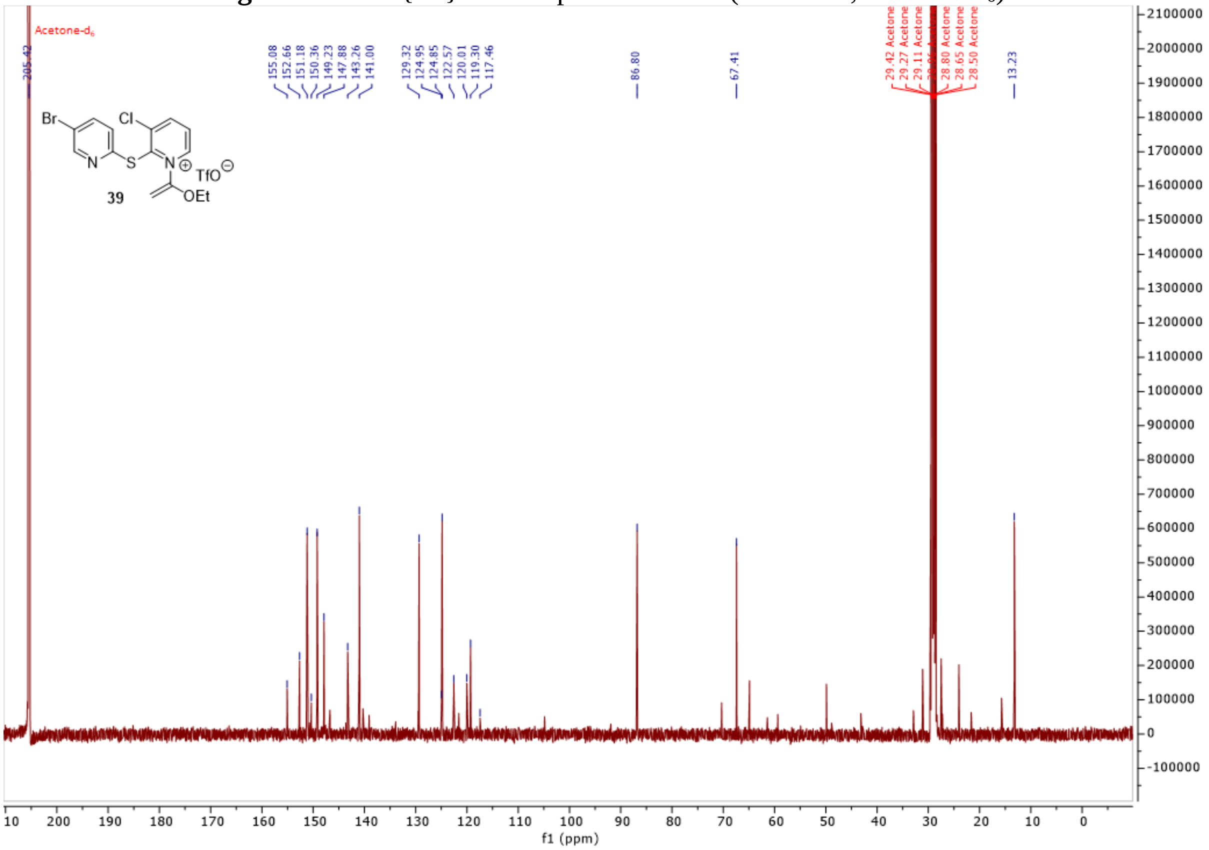

Figure S63. ^{1}H NMR Spectrum of **41** (500 MHz, Acetone-d_6)

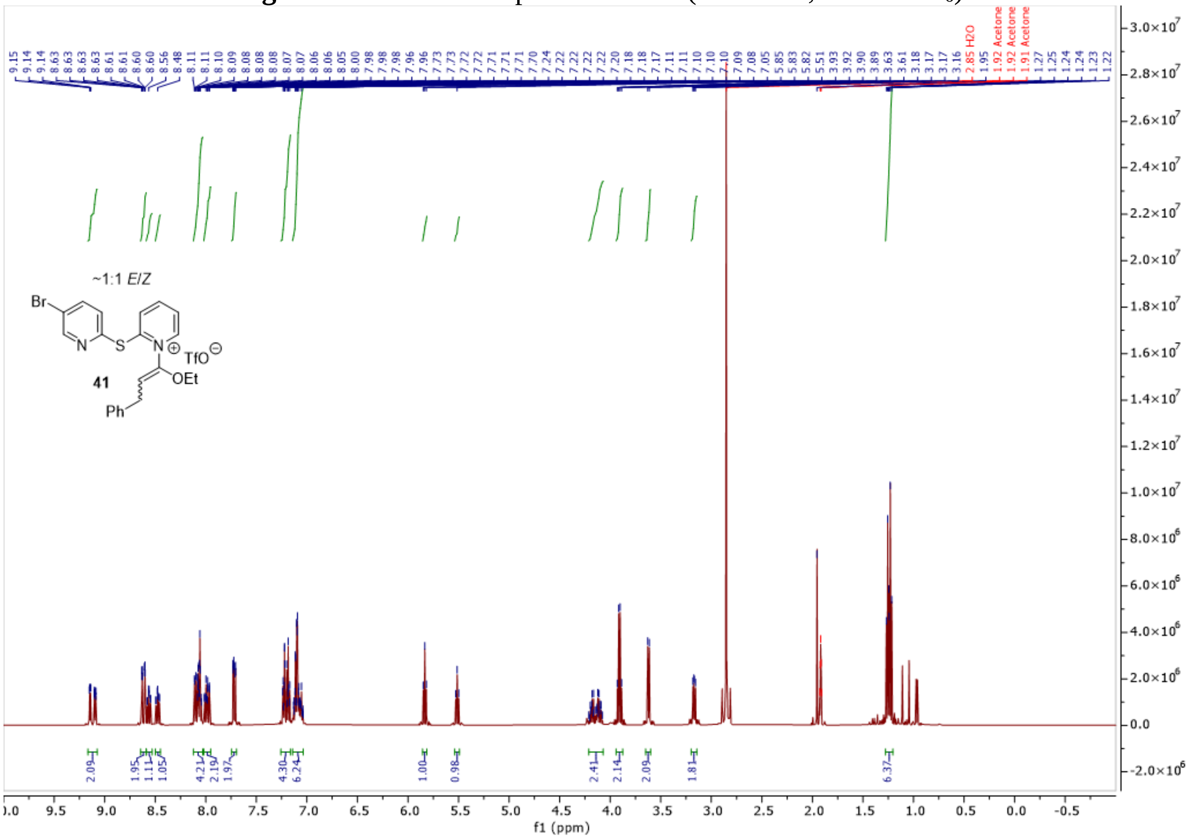

Figure S64. ^{13}C{^{1}H} NMR Spectrum of **41** (126 MHz, Acetone-d_6)

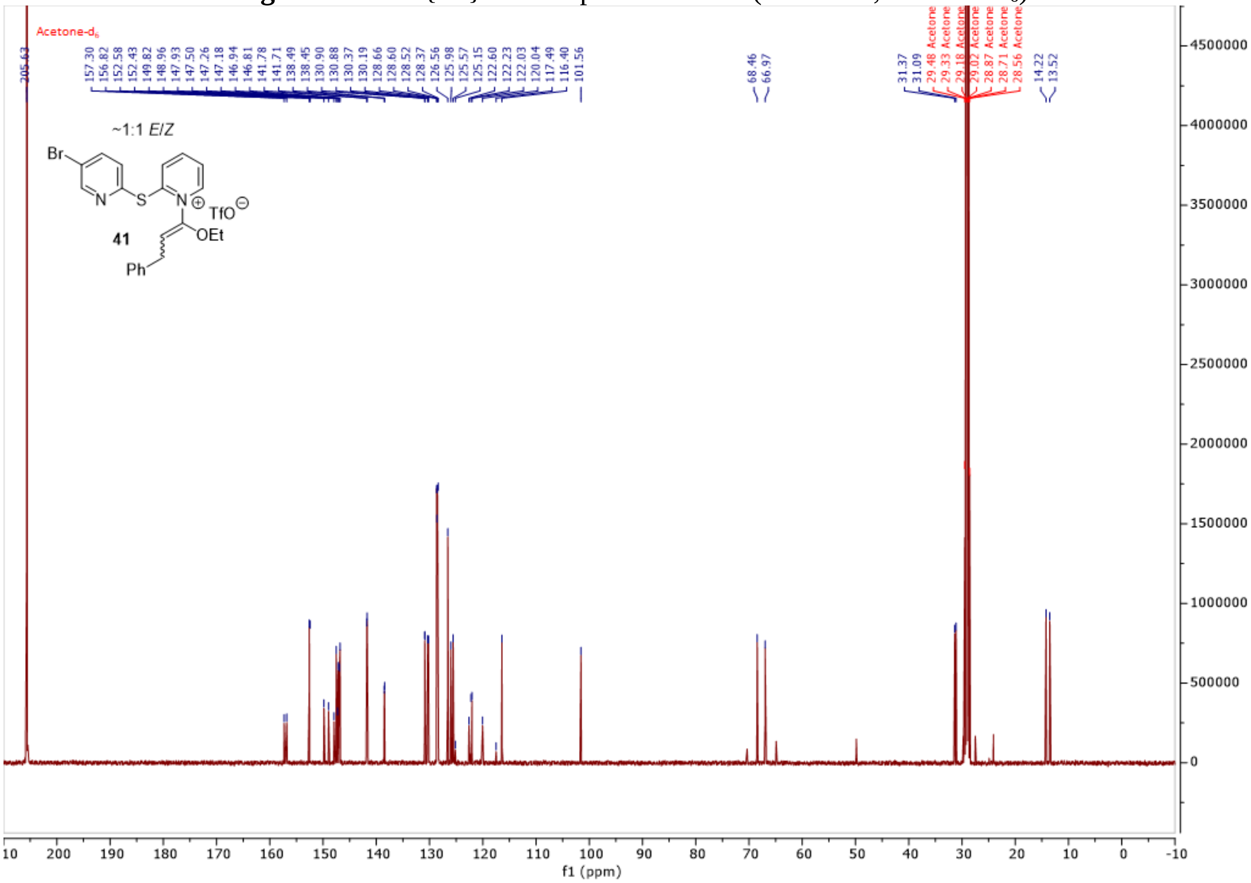

Figure S65. ^{1}H NMR Spectrum of (*E*)-**42** (500 MHz, Acetone-d$_6$)

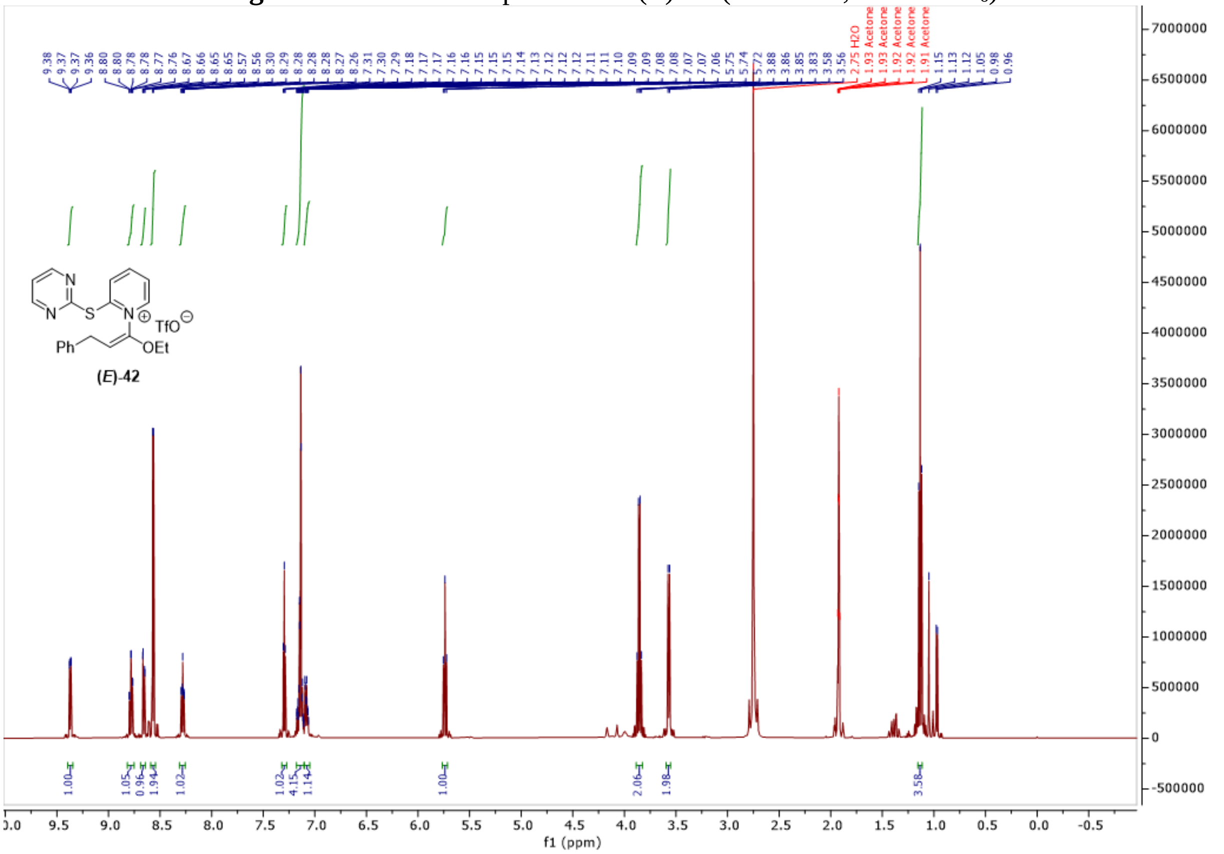

Figure S66. ^{13}C{^{1}H} NMR Spectrum of (*E*)-**42** (126 MHz, Acetone-d$_6$)

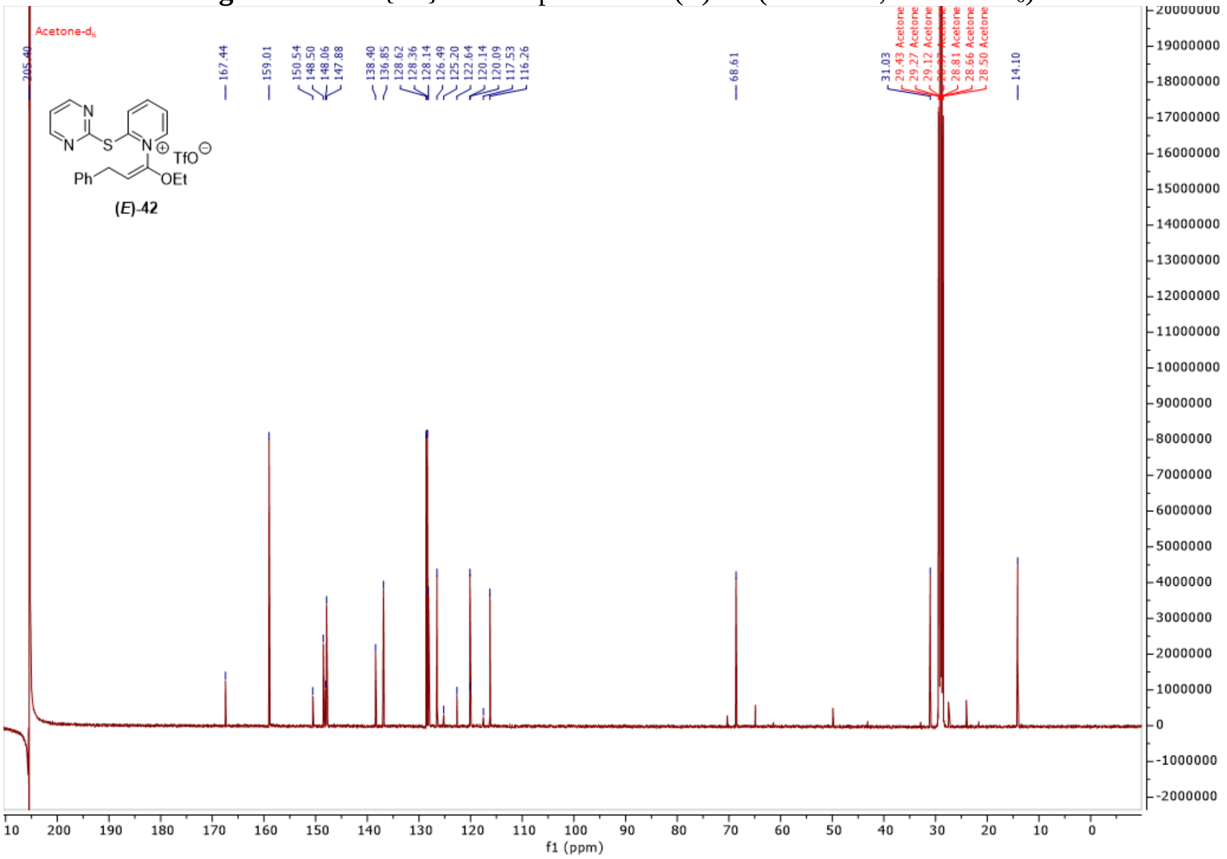

Figure S67. ^{1}H NMR Spectrum of (*Z*)-**42** (500 MHz, Acetone-d_6)

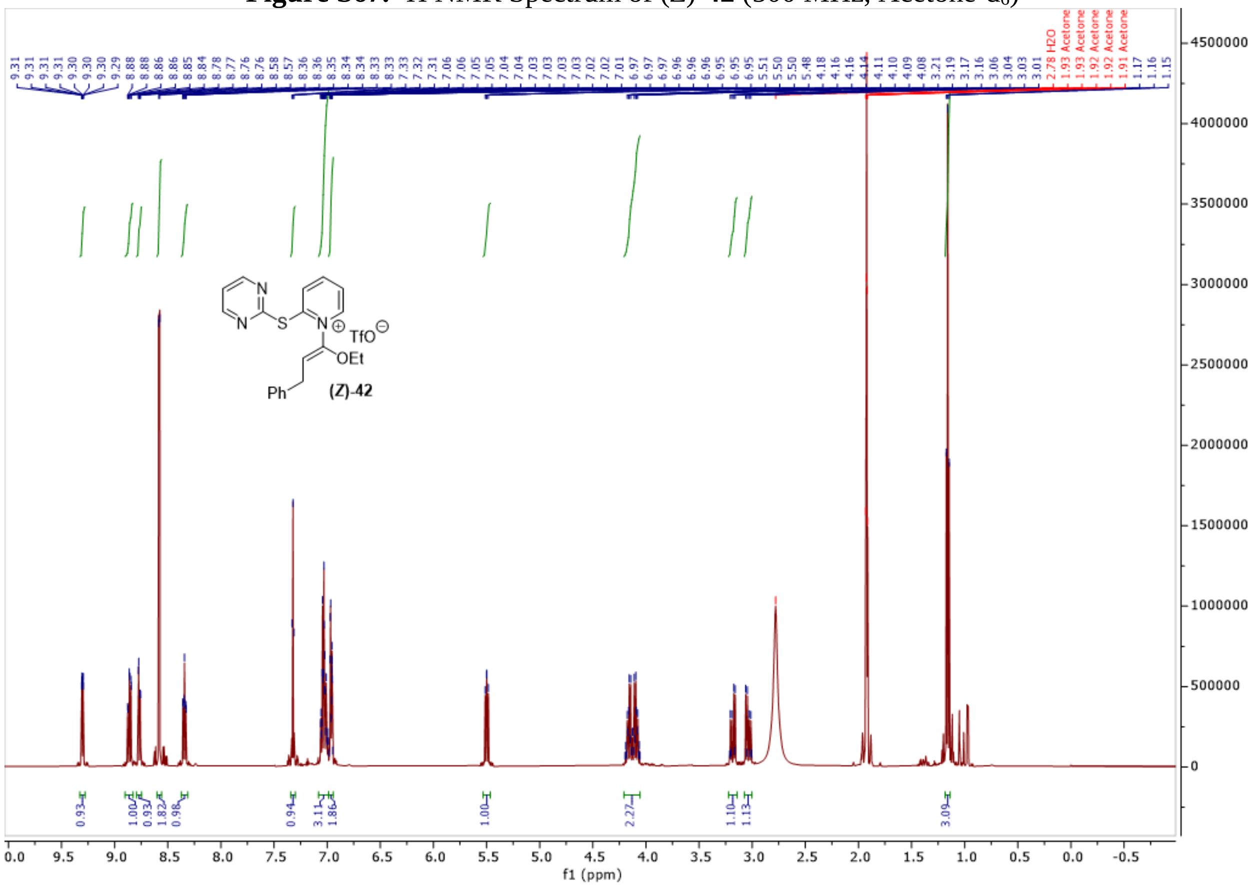

Figure S68. $^{13}C\{^{1}H\}$ NMR Spectrum of (Z)-**42** (126 MHz, Acetone-d_6)

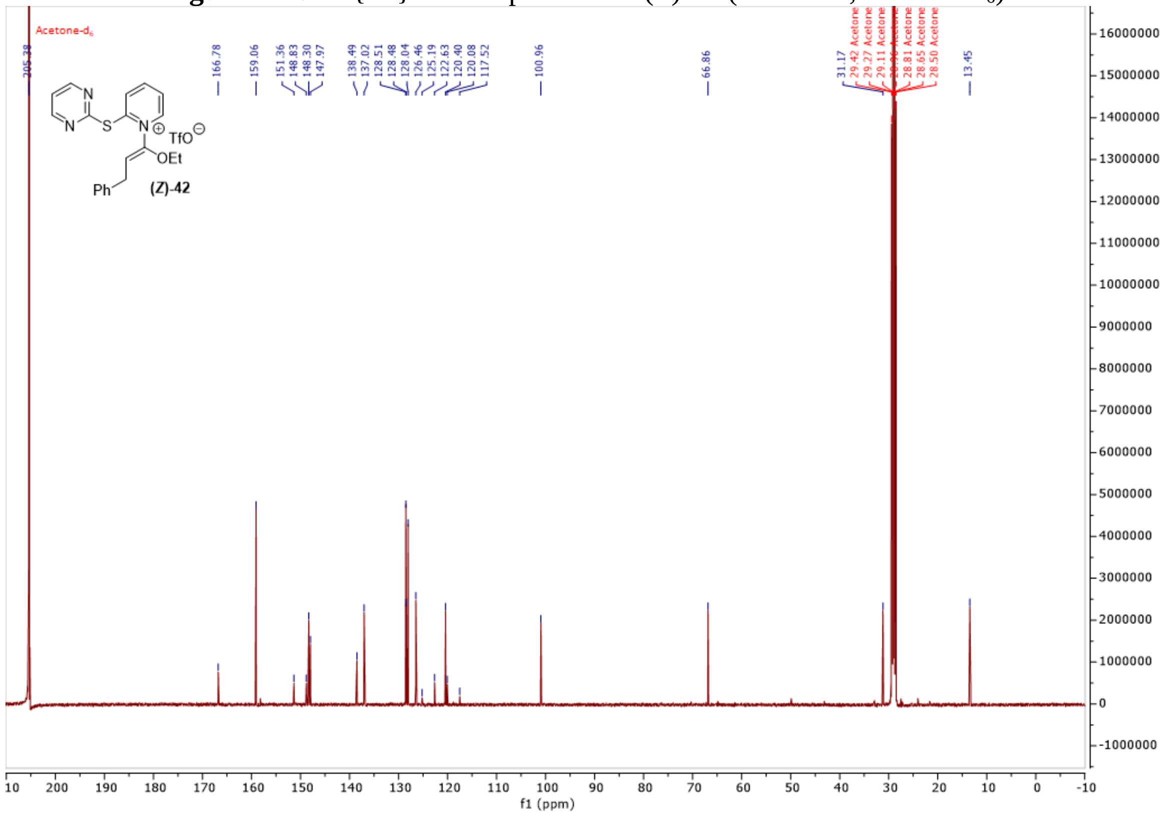

Figure S69. ^{1}H NMR Spectrum of **43** (500 MHz, Acetone-d_6)

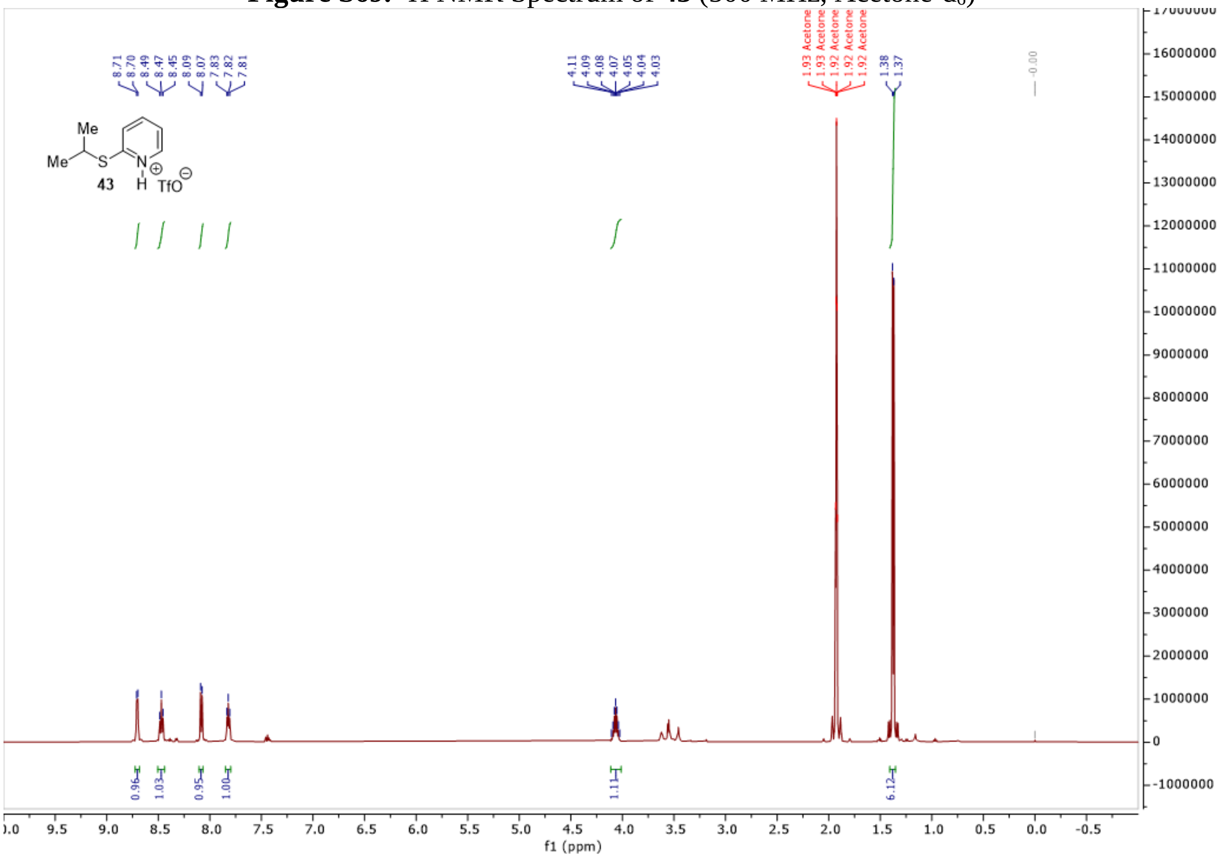

Figure S70. ^{13}C{^{1}H} NMR Spectrum of **43** (126 MHz, Acetone-d_6)

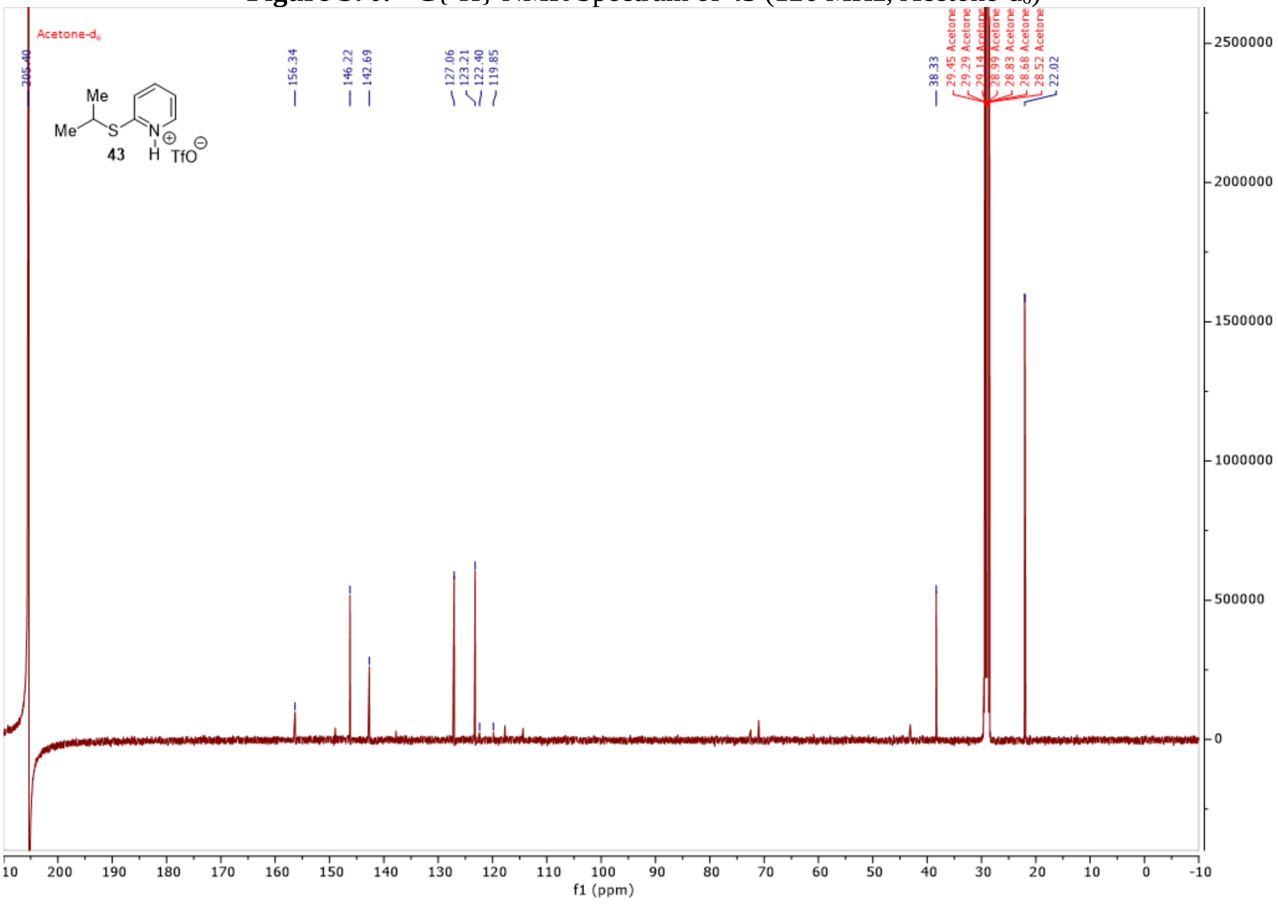

Figure S71.^{1}H NMR Spectrum of **44** (500 MHz, Acetone-d_6)

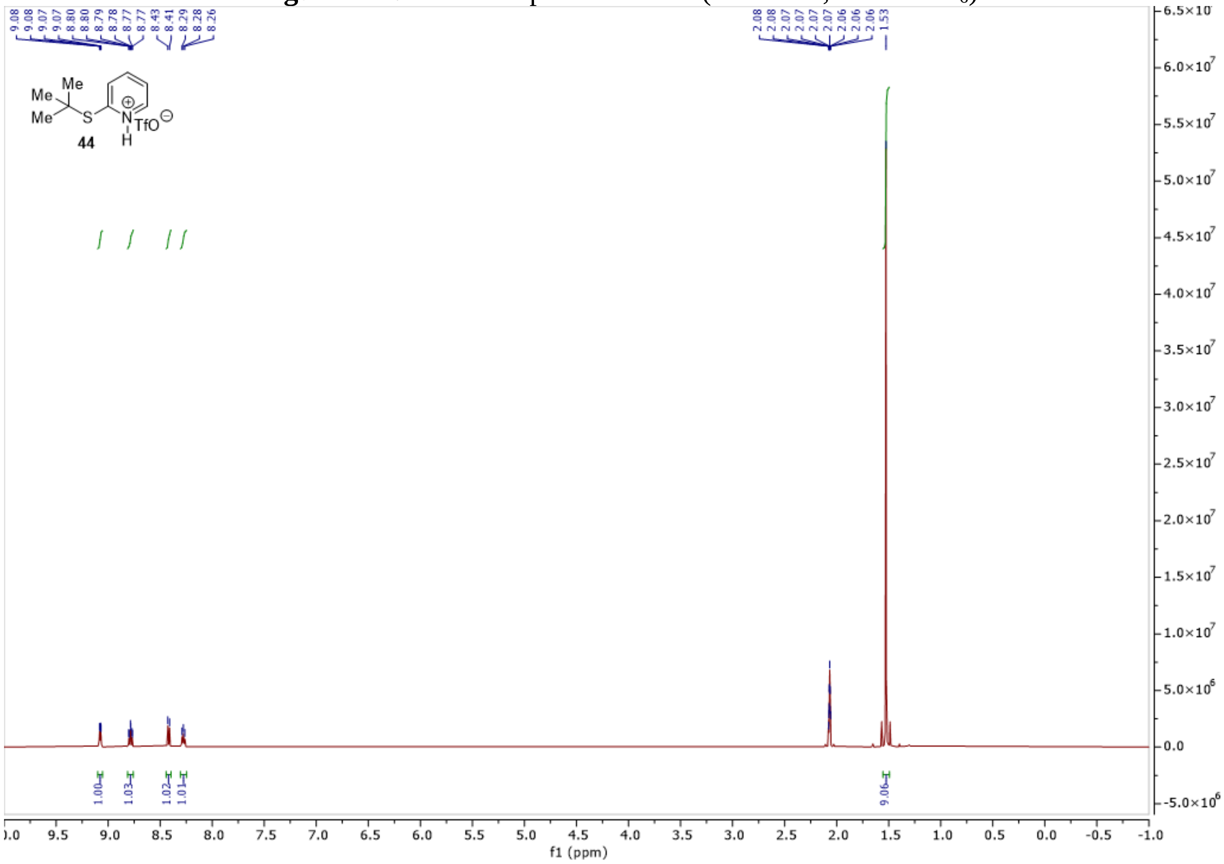

Figure S72. $^{13}C\{^1H\}$ NMR Spectrum of **44** (126 MHz, Acetone-d_6)

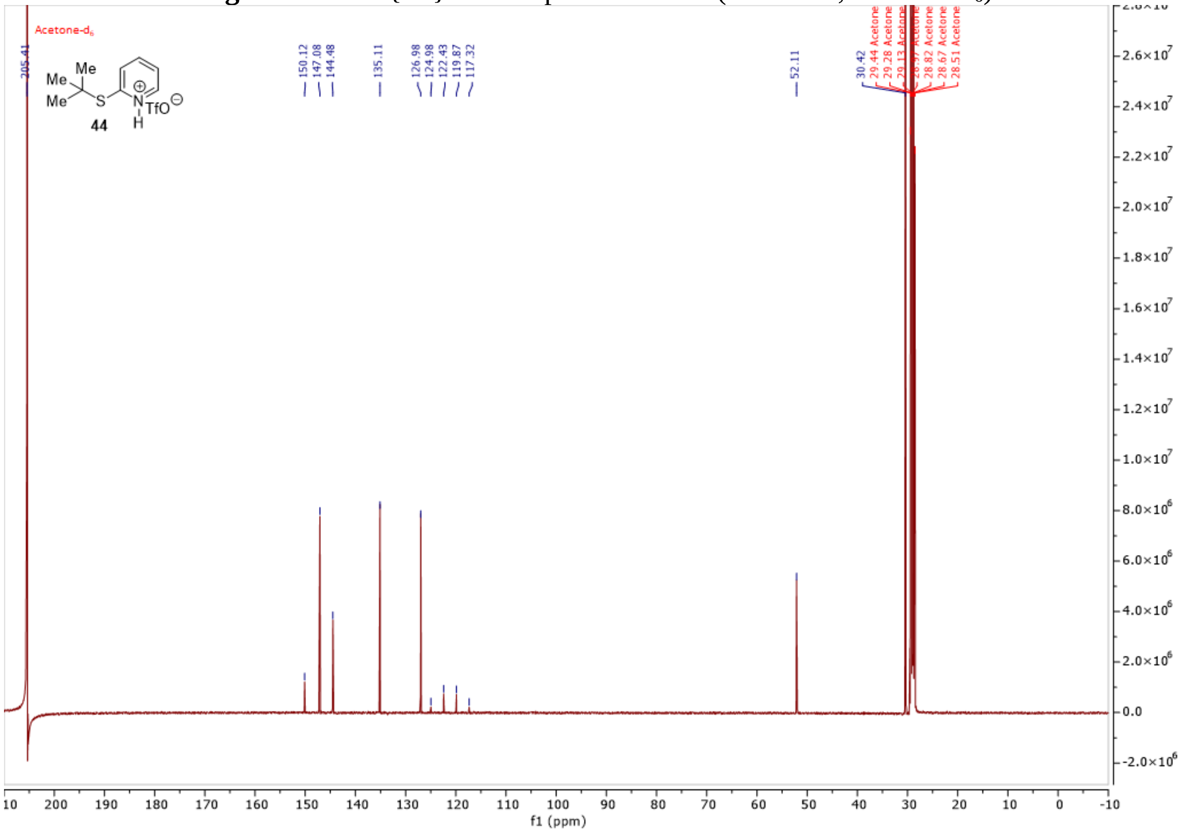

Figure S73. ^{1}H NMR Spectrum of **45** (500 MHz, Acetone-d_6)

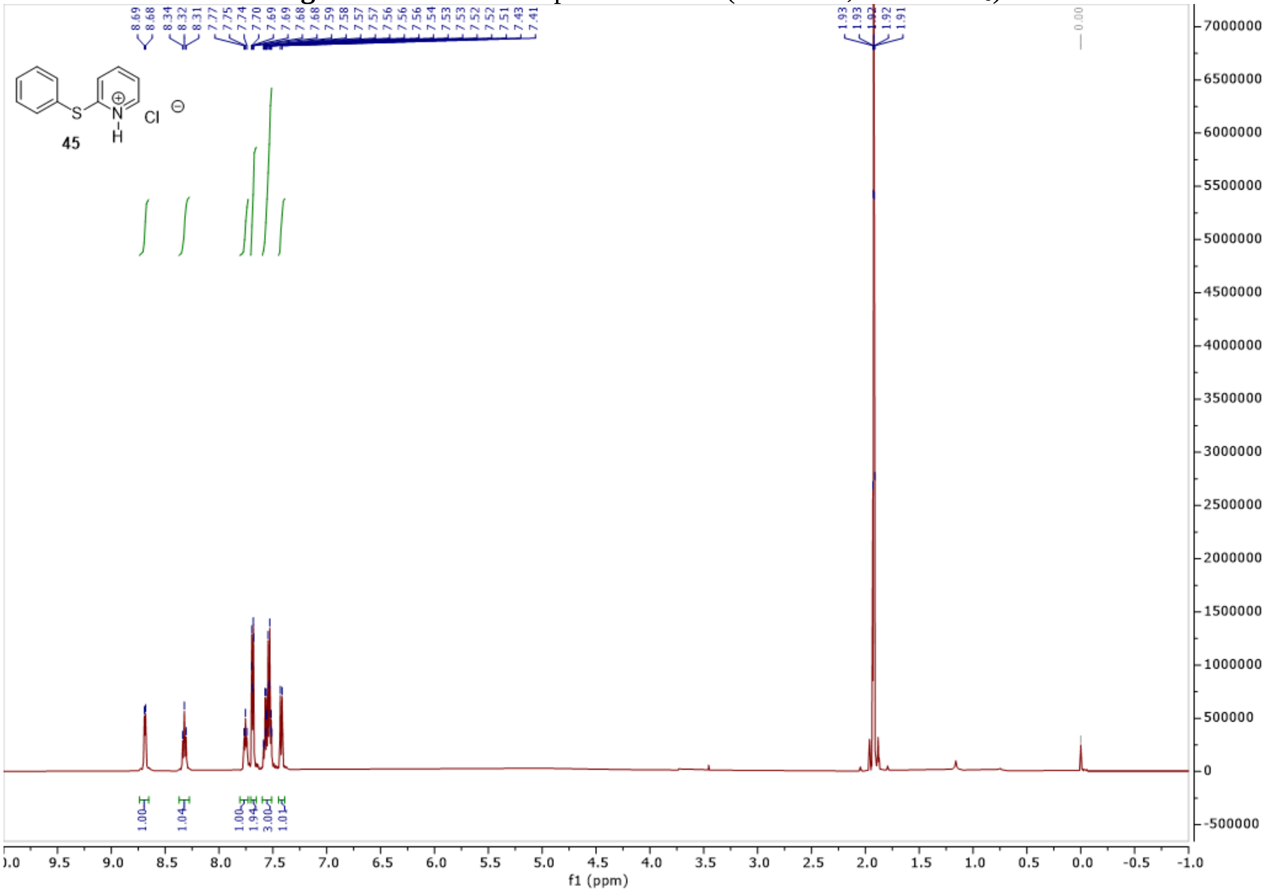

Figure S74. ^{13}C{^{1}H} NMR Spectrum of **45** (126 MHz, Acetone-d_6)

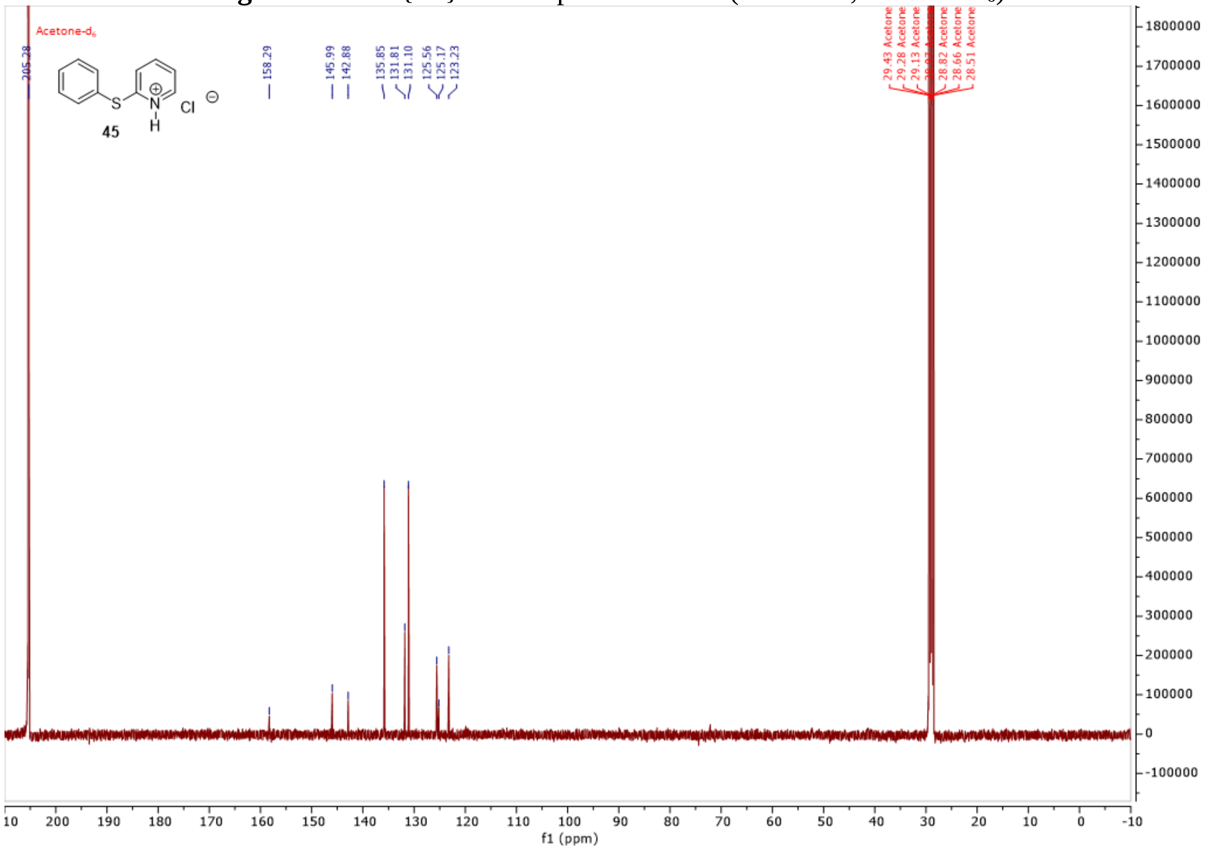

Figure S75. ^{1}H NMR Spectrum of **46** (500 MHz, Acetone-d_6)

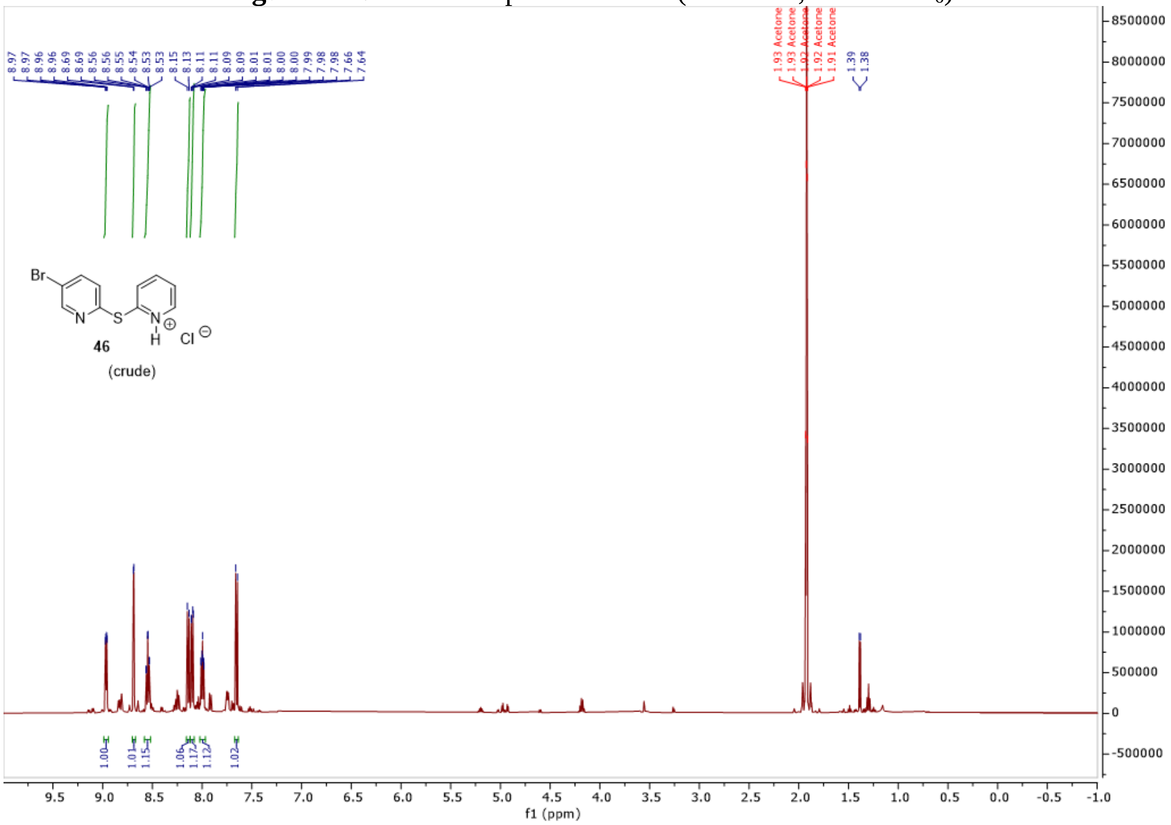

Figure S76. ^{13}C{^{1}H} NMR Spectrum of **46** (126 MHz, Acetone-d_6)

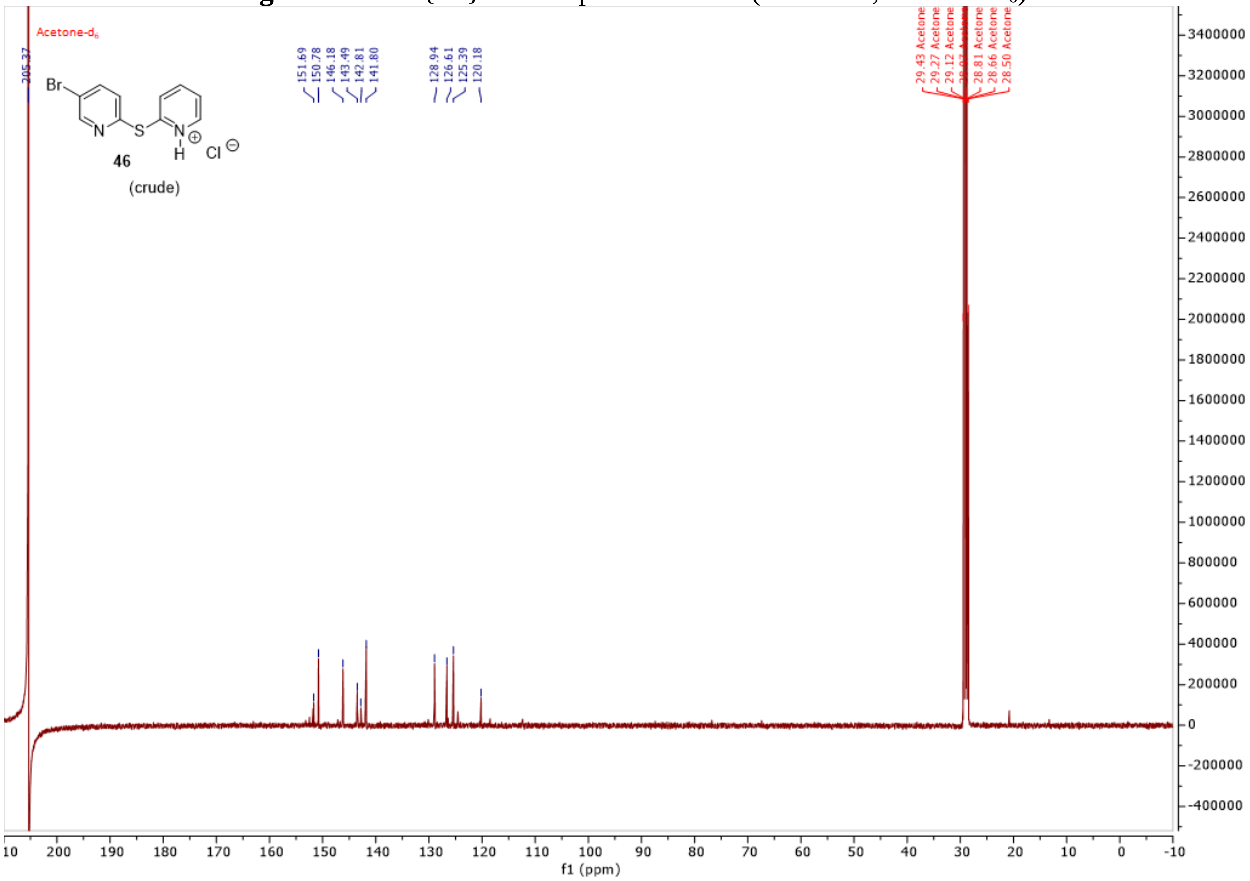

Figure S77. ^{1}H NMR Spectrum of **47** (500 MHz, Acetone-d_6)

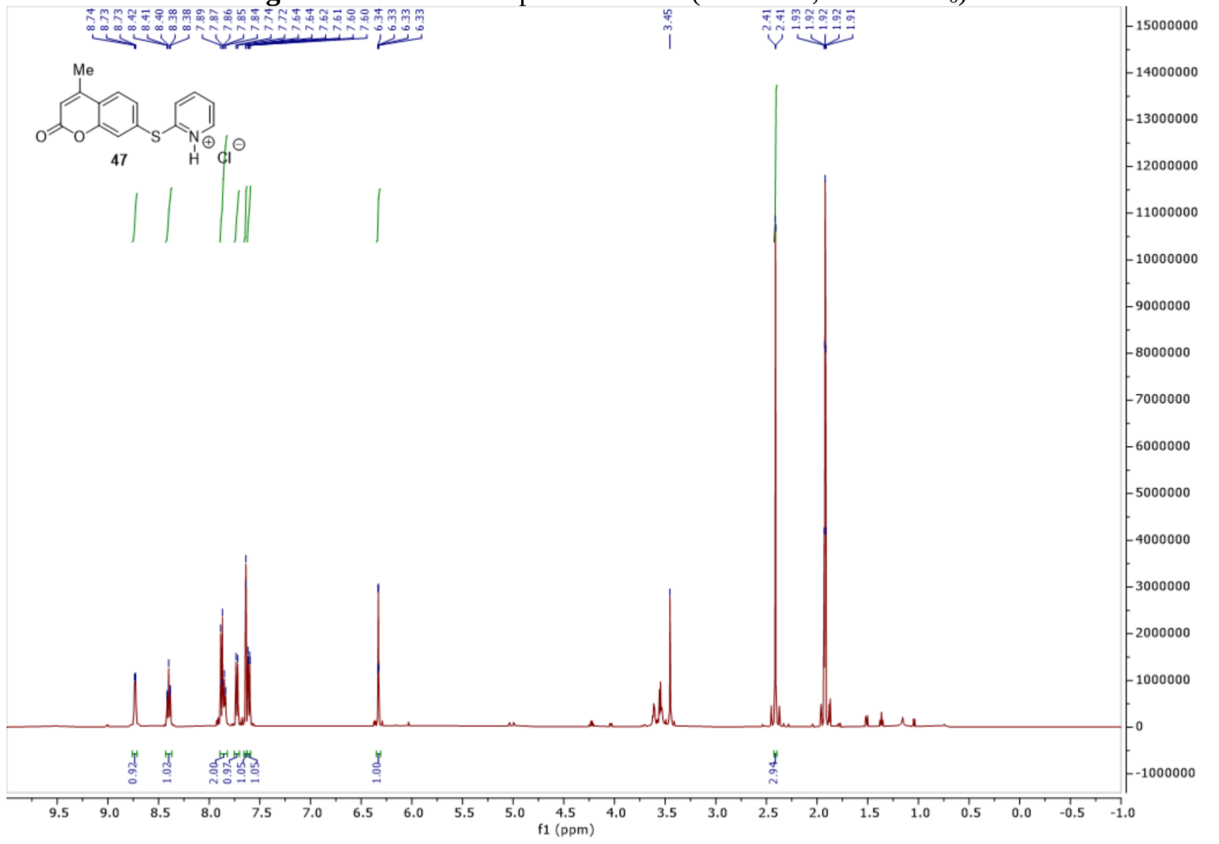

Figure S78. ^{13}C{^{1}H} NMR Spectrum of **47** (126 MHz, Acetone-d_6)

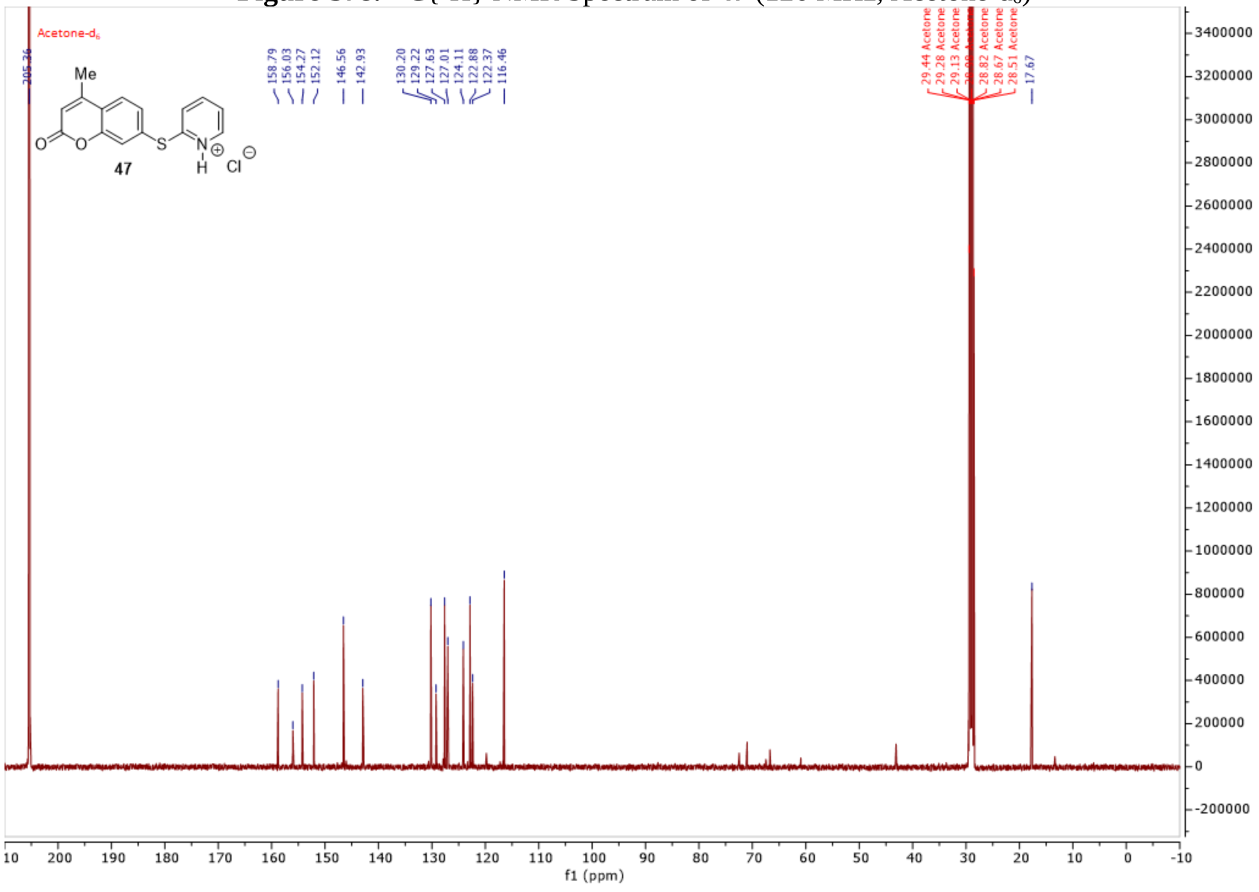

Figure S79. ^{1}H NMR Spectrum of **48** (500 MHz, Acetone-d_6)

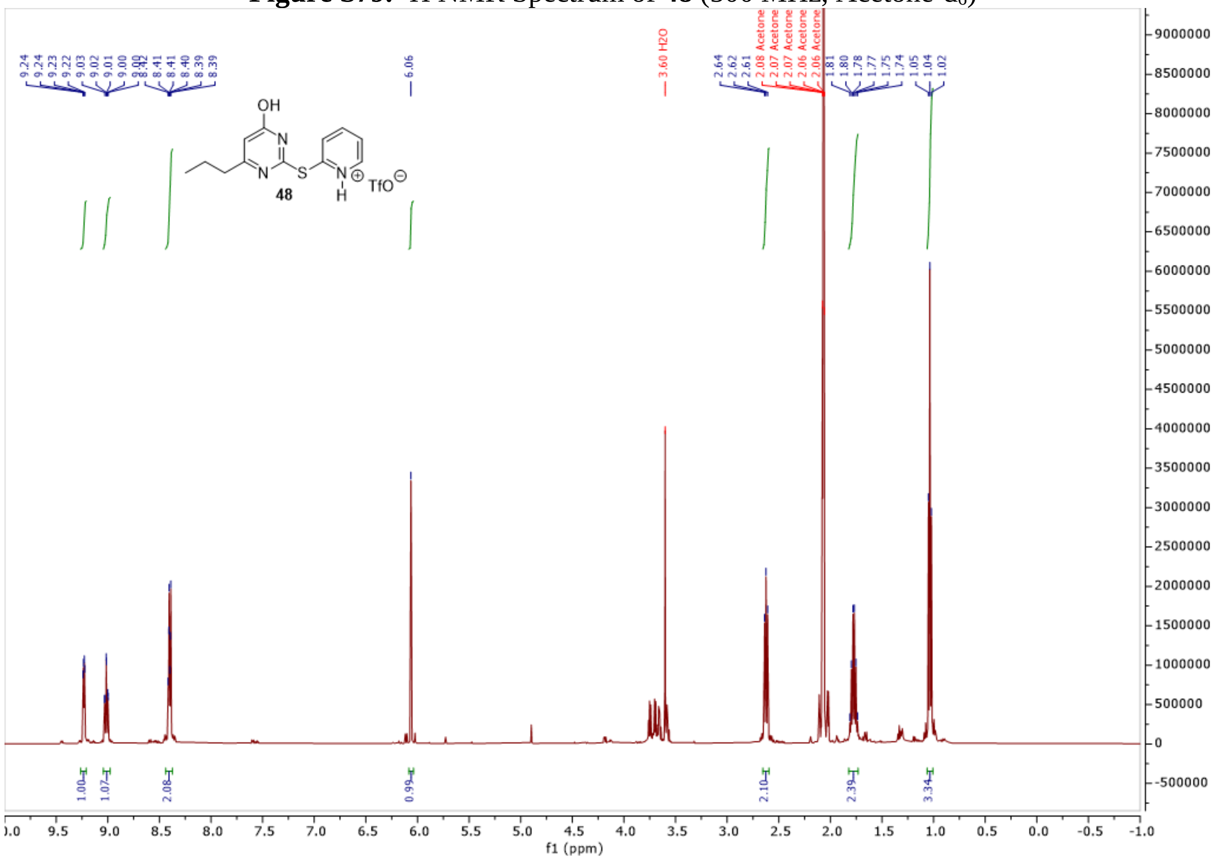

Figure S80. ^{13}C{^{1}H} NMR Spectrum of **48** (126 MHz, Acetone-d_6)

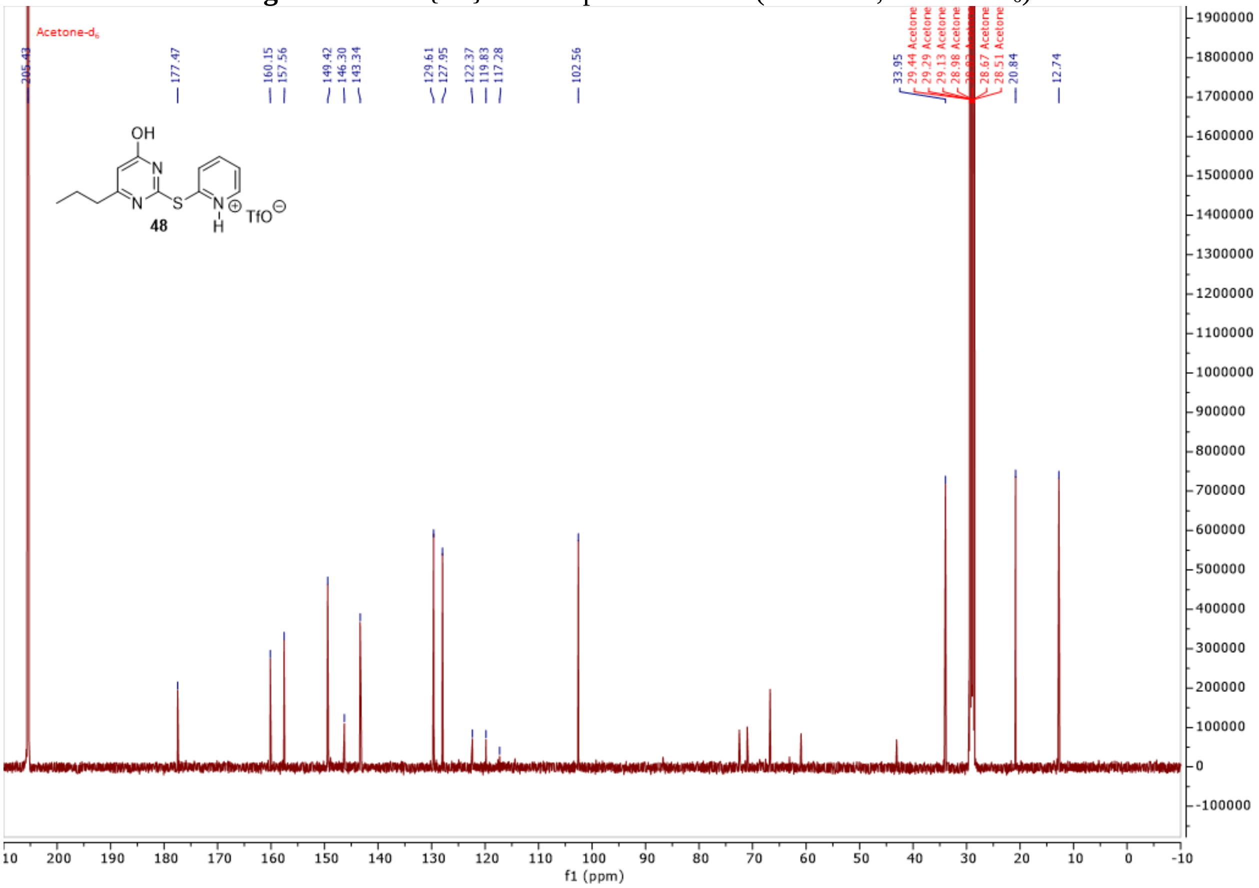

Figure S81. ^{1}H NMR Spectrum of **49** (500 MHz, Acetone-d_6)

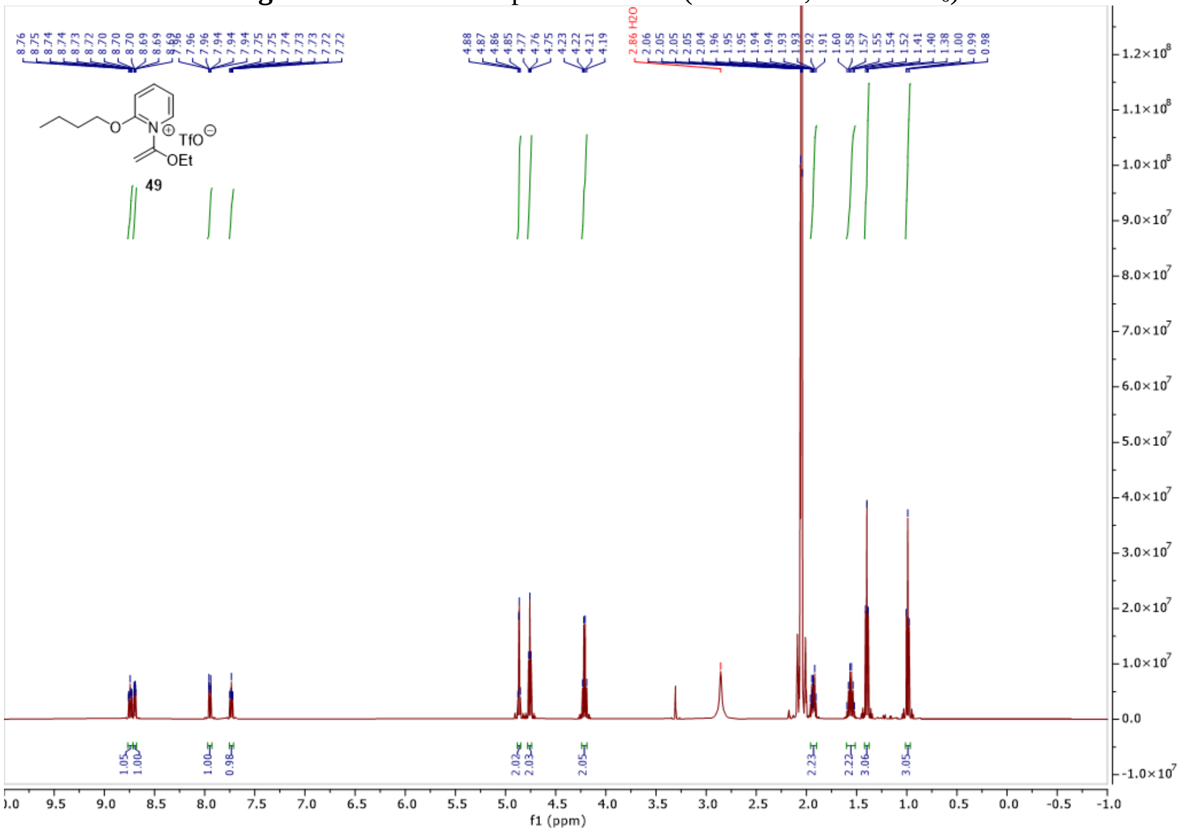

Figure S82. ^{13}C{^{1}H} NMR Spectrum of **49** (126 MHz, Acetone-d_6)

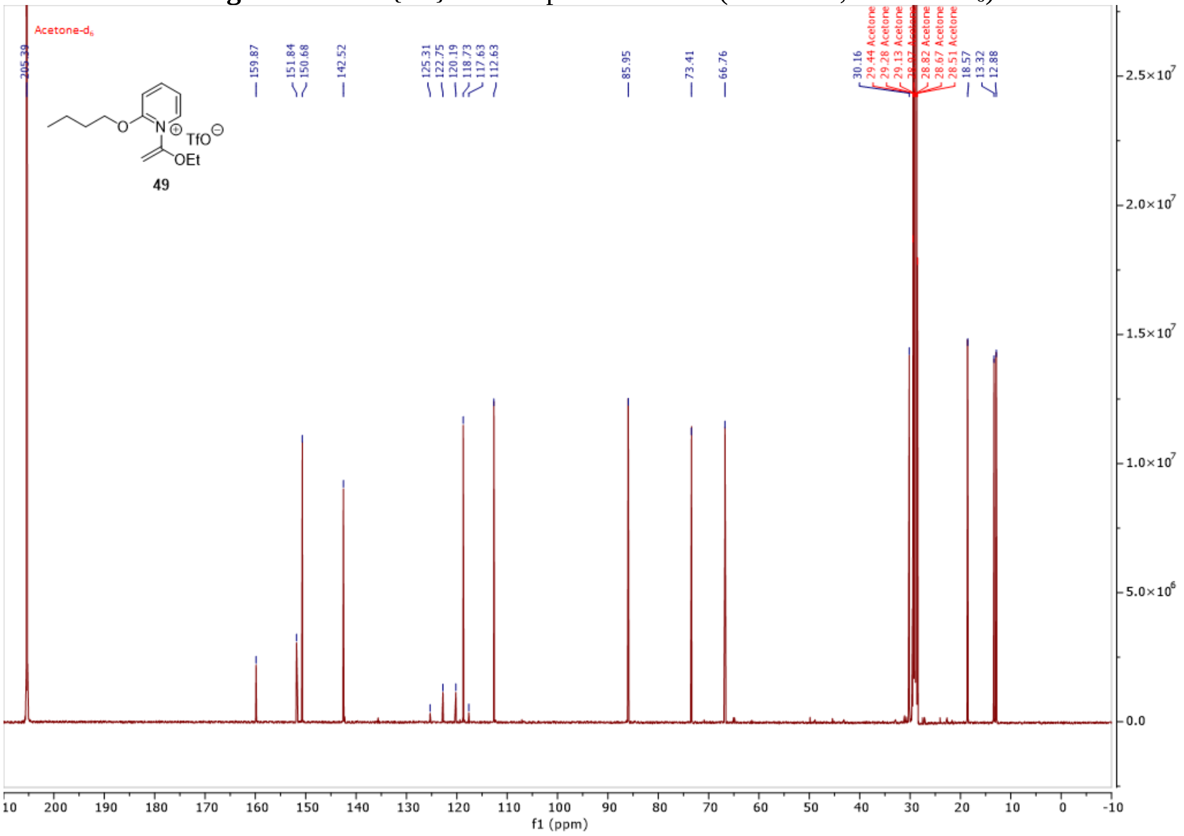

Figure S83. ^{1}H NMR Spectrum of **50** (500 MHz, Acetone-d_6)

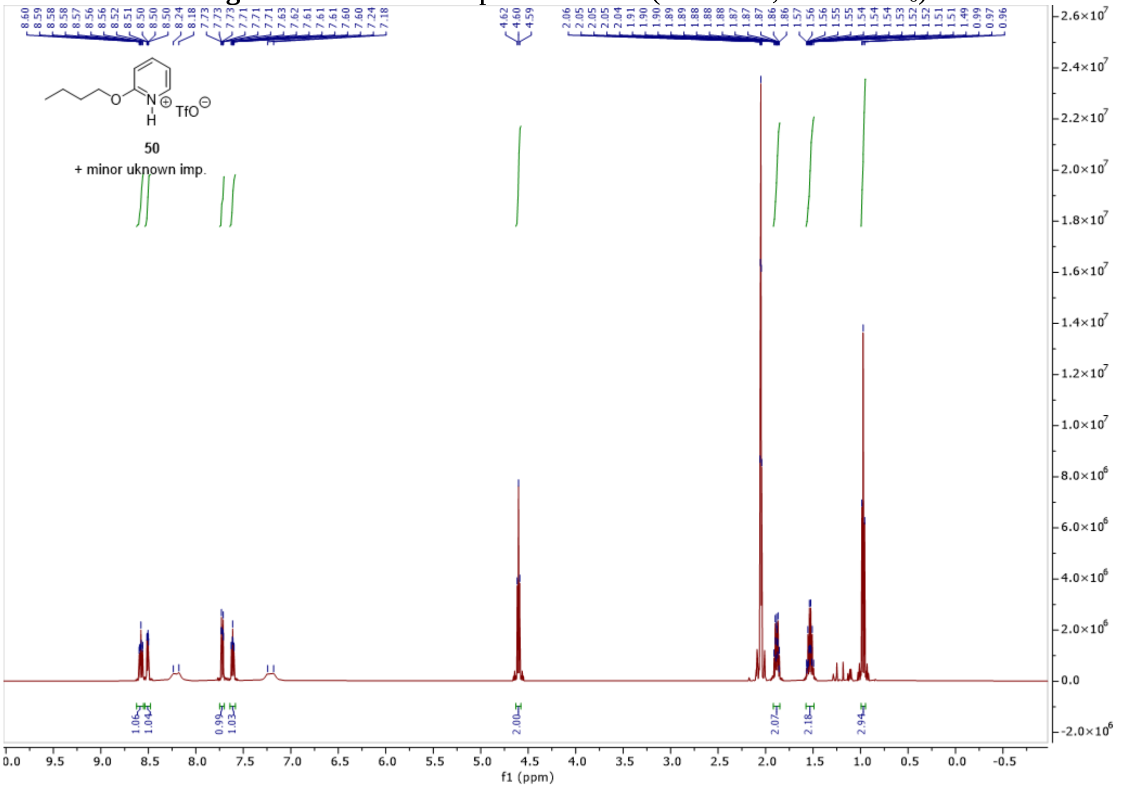

Figure S84. ^{13}C{^{1}H} NMR Spectrum of **50** (126 MHz, Acetone-d_6)

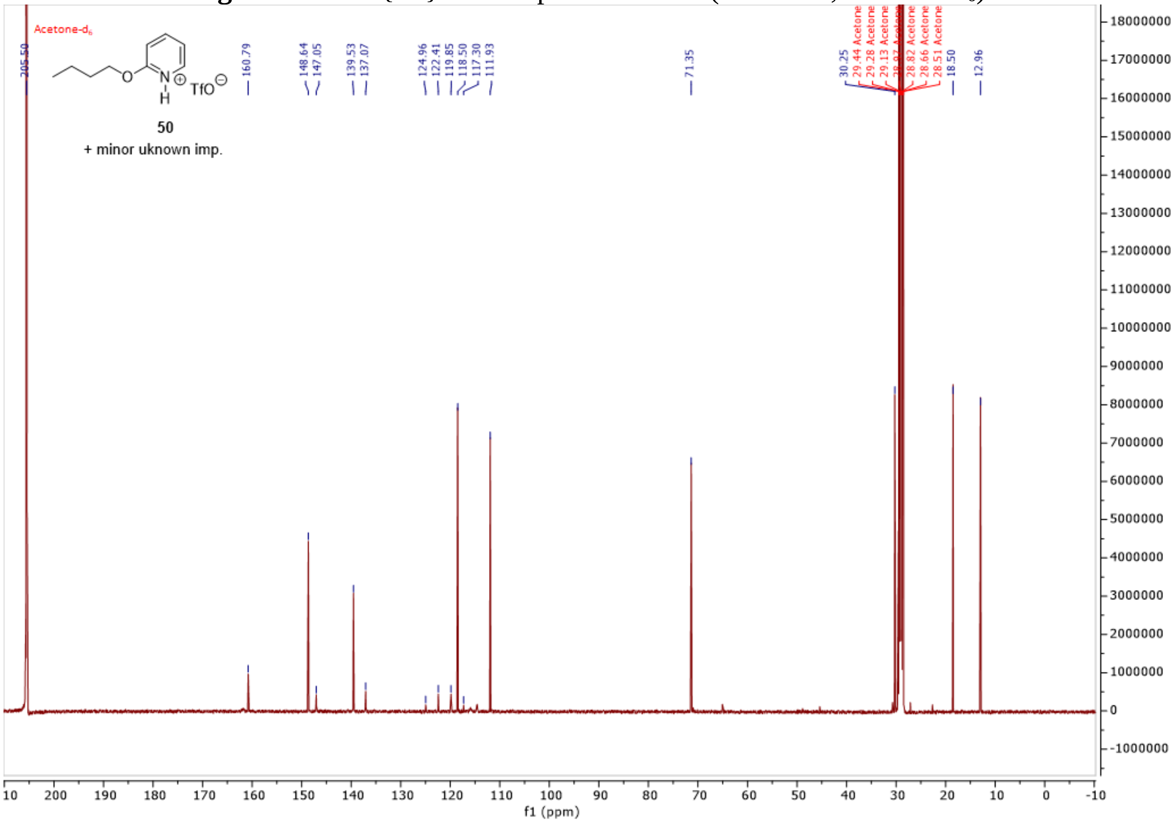

Supplement: Supplementary file 1 [file ao5c07504_si_001.pdf]
